# Supplementary material for: Novel Brassinosteroid Analogues with 3,6 Dioxo Function, 24-Nor-22(S)-Hydroxy Side Chain and p-Substituted Benzoate Function at C-23—Synthesis and Evaluation of Plant Growth Effects
Source: Int J Mol Sci. 2024 Jul 9;25(14):7515. doi: 10.3390/ijms25147515 (PMC11276741; doi:10.3390/ijms25147515)
Supplement: Supplementary file 1 [file ijms-25-07515-s001.zip › ijms-3047106-supplementary.pdf]

International Journal of
Molecular Sciences

MDPI

Article

Novel Brassinosteroid Analogues with 3,6 Dioxo Function, 24-Nor-22(S)-Hydroxy Side Chain and p-Substituted Benzoate Function at C-23—Synthesis and Evaluation of Plant Growth Effects

Sebastián Jorquera 1, Mauricio Soto 1 , Katy Díaz 1 , María Nuñez 1, Mauricio A. Cuellar 2,3, Andrés F. Olea 4,* and Luis Espinoza-Catalán 1,*

1 Departamento de Química, Universidad Técnica Federico Santa María, Avenida España 1680, Valparaíso 2340000, Chile; sebastian.jorquera@sansano.usm.cl (S.J.); mauricio.sotoc@usm.cl (M.S.); katy.diaz@usm.cl (K.D.); maria.nunezg@sansano.usm.cl (M.N.)

2 Facultad de Farmacia, Escuela de Química y Farmacia, Universidad de Valparaíso, Av. Gran Bretaña 1093, Valparaíso 2340000, Chile; mauricio.cuellar@uv.cl

3 Centro de Investigación, Desarrollo e Innovación de Productos Bioactivos (CINBIO), Universidad de Valparaíso, Valparaíso 2340000, Chile

4 Grupo QBAB, Instituto de Ciencias Químicas Aplicadas, Facultad de Ingeniería, Universidad Autónoma de Chile, El Llano Subercaseaux 2801, Santiago 8900000, Chile

* Correspondence: andres.olea@uautonoma.cl (A.F.O.); luis.espinozac@usm.cl (L.E.-C.); Tel.: +56-32-2654425 (L.E.-C.)

Index **pag.**

S1. ^{1}H NMR spectrum compound **42**.....4

S2. ^{13}C NMR spectrum compound **42**.....5

S3. ^{13}C DEPT-135 NMR spectrum compound **42**.....6

S4. 2D HSQC spectrum compound **42**.....7

S5. 2D HMBC spectrum compound **42**.....8

S6. ^{1}H NMR spectrum compound **43**.....9

S7. ^{13}C NMR spectrum compound **43**.....10

S8. ^{13}C DEPT-135 NMR spectrum compound **43**.....11

S9. 2D HSQC spectrum compound **43**.....12

S10. 2D HMBC spectrum compound **43**.....13

S11. ^{1}H NMR spectrum compound **44**.....14

S12. ^{13}C NMR spectrum compound **44**.....15

S13. ^{13}C DEPT-135 NMR spectrum compound **44**.....16

S14. 2D HSQC spectrum compound **44**.....17

S15. 2D HMBC spectrum compound **44**.....18

S16. ^{1}H NMR spectrum mixture **40/40a**……………………………………………………19

S17. ^{1}H NMR spectrum expansion and proportion determination for **40**:**40a** = 4:1 epimers mixture……………………20

S18. ^{13}C NMR spectrum mixture **40/40a**……………………………………………………21

S19. ^{13}C DEPT-135 NMR spectrum mixture **40/40a**……………………………………………………22

S20. 2D HSQC spectrum mixture **40/40a**……………………………………………………23

S21. 2D HMBC spectrum mixture **40/40a**……………………………………………………24

S22. ^{1}H NMR spectrum compound **40**……………………………………………………25

S23. ^{13}C NMR spectrum compound **40**……………………………………………………26

S24. ^{13}C DEPT-135 NMR spectrum compound **40**……………………………………………………27

S25. 2D HSQC spectrum compound **40**……………………………………………………28

S26. 2D HMBC spectrum compound **40**……………………………………………………29

S27. ^{1}H NMR spectrum compound **41a**……………………………………………………30

S28. ^{13}C NMR spectrum compound **41a**……………………………………………………31

S29. ^{13}C DEPT-135 NMR spectrum compound **41a**……………………………………………………32

S30. 2D HSQC spectrum compound **41a**……………………………………………………33

S31. 2D HMBC spectrum compound **41a**……………………………………………………34

S32. ^{1}H NMR spectrum compound **41b**……………………………………………………35

S33. ^{13}C NMR spectrum compound **41b**……………………………………………………36

S34. ^{13}C DEPT-135 NMR spectrum compound **41b**……………………………………………………37

S35. 2D HSQC spectrum compound **41b**……………………………………………………38

S36. 2D HMBC spectrum compound **41b**……………………………………………………39

S37. ^{1}H NMR spectrum compound **41c**……………………………………………………40

S38. ^{13}C NMR spectrum compound **41c**……………………………………………………41

S39. ^{13}C DEPT-135 NMR spectrum compound **41c**……………………………………………………42

S40. 2D HSQC spectrum compound **41c**……………………………………………………43

S41. 2D HMBC spectrum compound **41c**……………………………………………………44

S42. ^{1}H NMR spectrum compound **41d**……………………………………………………45

S43. ^{13}C NMR spectrum compound **41d**……………………………………………………46

S44. ^{13}C DEPT-135 NMR spectrum compound **41d**……47

S45. 2D HSQC spectrum compound **41d**……48

S46. 2D HMBC spectrum compound **41d**……49

S47. ^{1}H NMR spectrum compound **41e**……50

S48. ^{13}C NMR spectrum compound **41e**……51

S49. ^{13}C DEPT-135 NMR spectrum compound **41e**……52

S50. 2D HSQC spectrum compound **41e**……53

S51. 2D HMBC spectrum compound **41e**……54

S52. ^{1}H NMR spectrum compound **41f**……55

S53. ^{13}C NMR spectrum compound **41f**……56

S54. ^{13}C DEPT-135 NMR spectrum compound **41f**……57

S55. 2D HSQC spectrum compound **41f**……58

S56. 2D HMBC spectrum compound **41f**……59

S57. HRSM-ESI spectra of compound **40**……60

S58. HRSM-ESI spectra of compound **41a**……60

S59. HRSM-ESI spectra of compound **41b**……61

S60. HRSM-ESI spectra of compound **41c**……61

S61. HRSM-ESI spectra of compound **41d**……62

S62. HRSM-ESI spectra of compound **41e**……62

S63. HRSM-ESI spectra of compound **41f**……63

S64. Effect of BRs Analogs on the Rice Lamina Inclination of compounds **1**, **40** and **41a-41f** ……64

S65. Effect of BRs Analogs on Elongation Bean Second-Internode Bioassay of compounds **1**, **40** and **41a-41f**……65

S66. Predicted binding mode of compound **1** and **41f** ……65

S67. Table 1. Pose analysis of docked brassinolide (**1**) and synthetic analog **41f**……66

S68. Table 2. Docked compounds-heterodimer protein of **1** and synthetic analog **41f** ……67

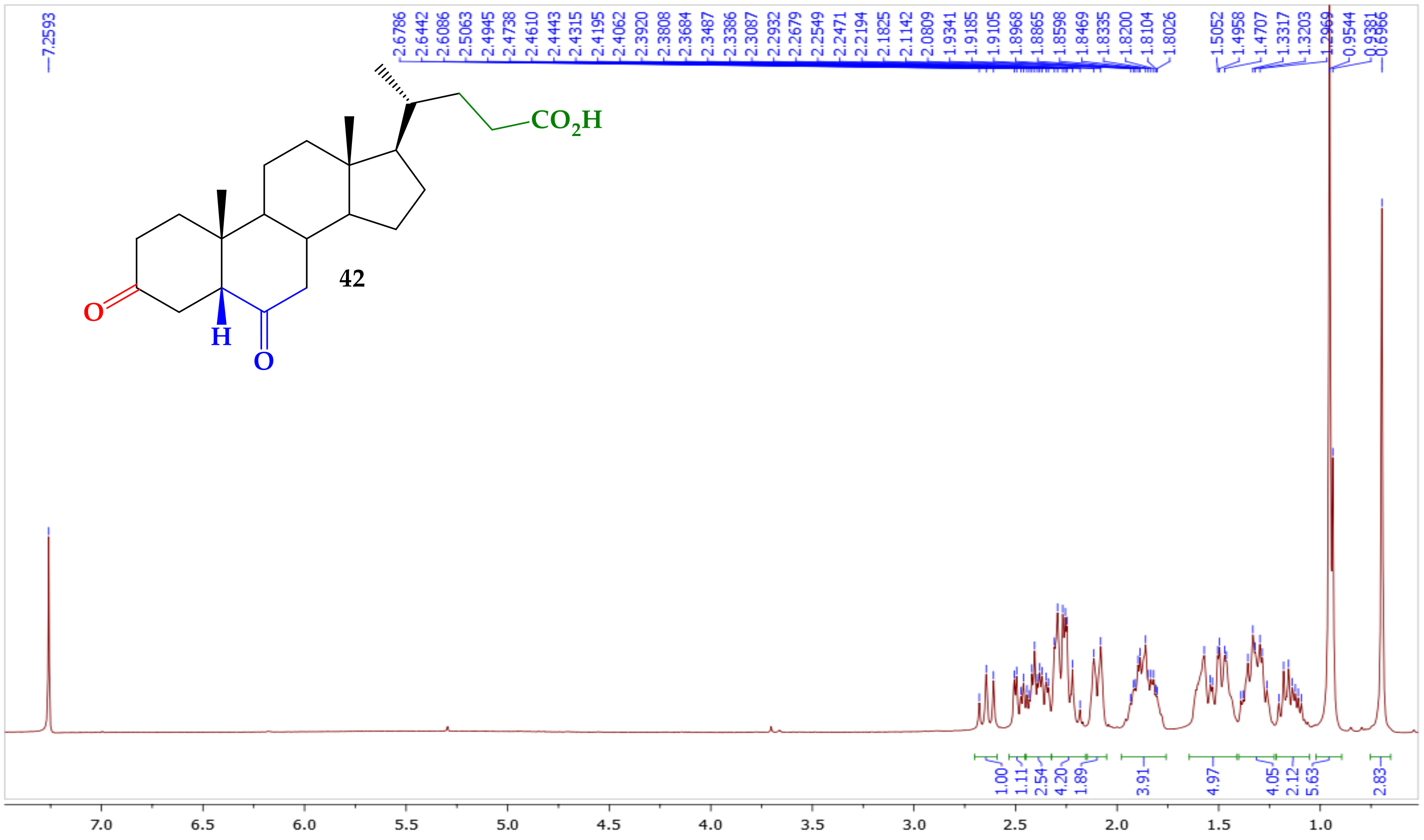

S1. ^{1}H NMR spectrum of *3,6-dioxo-5β-cholan-24-oic acid* (**42**)

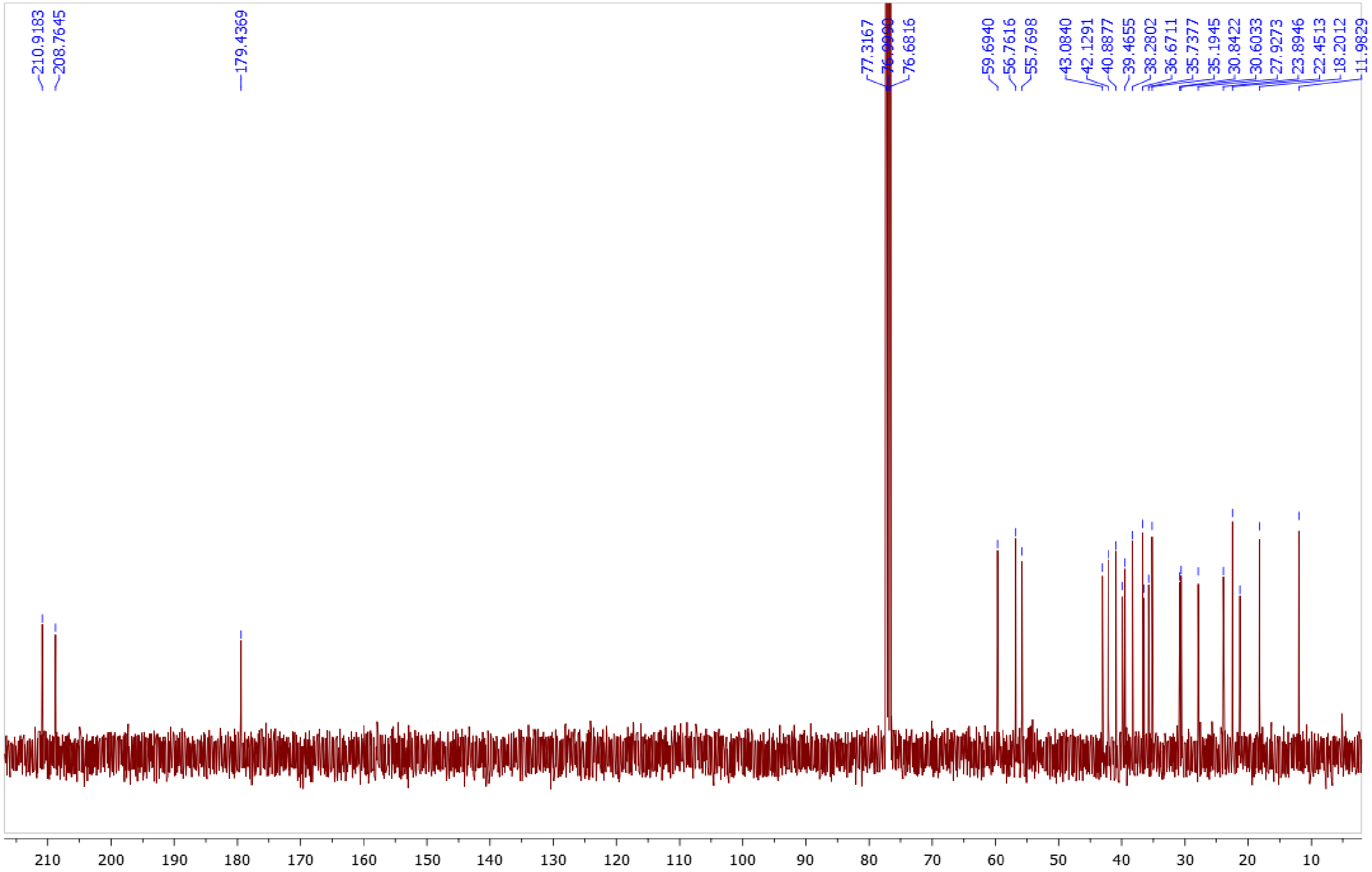

S2. ^{13}C NMR spectrum of *3,6-dioxo-5β-cholan-24-oic acid* (**42**)

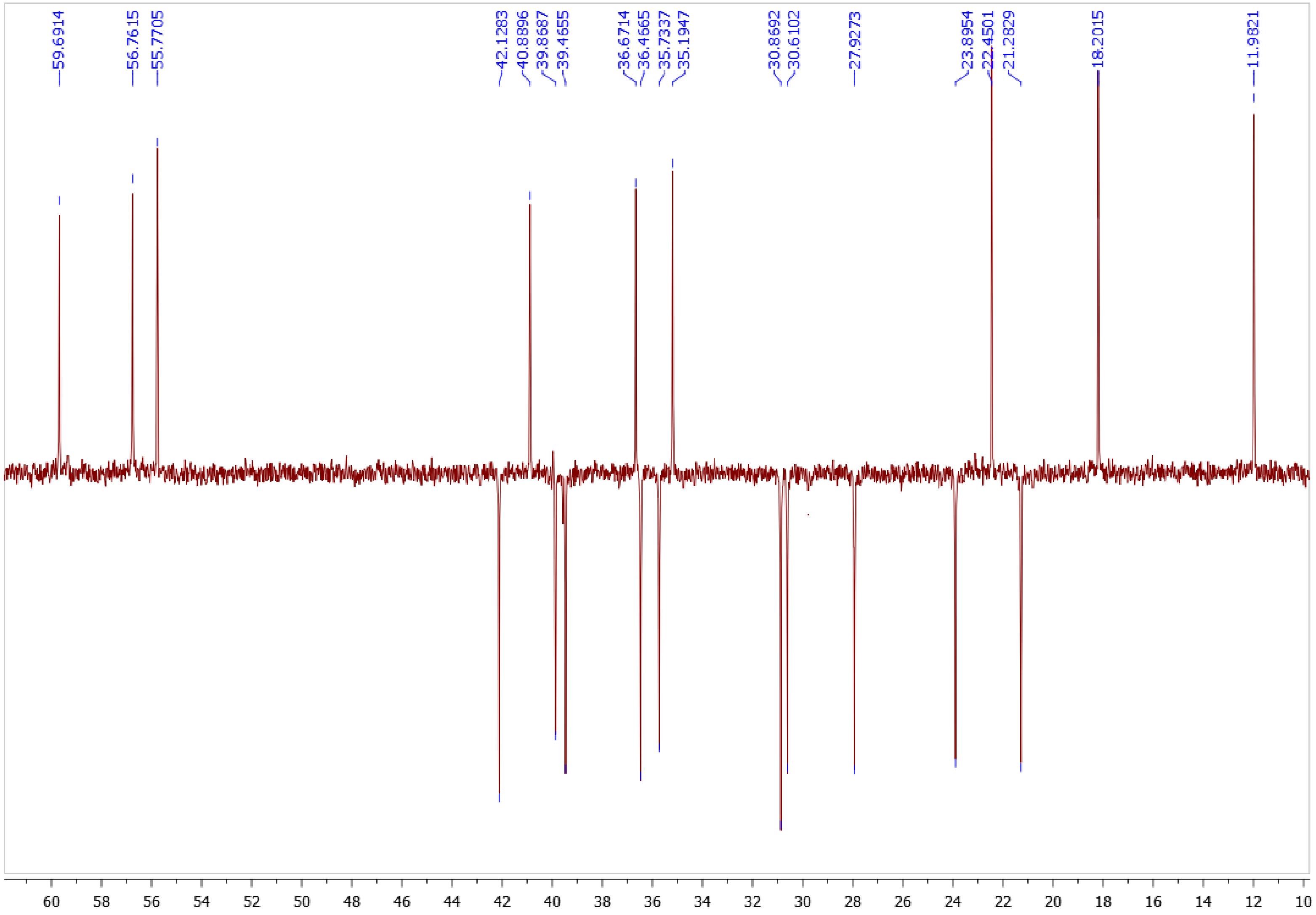

S3. ^{13}C DEPT-135 NMR spectrum of *3,6-dioxo-5β-cholan-24-oic acid* (**42**)

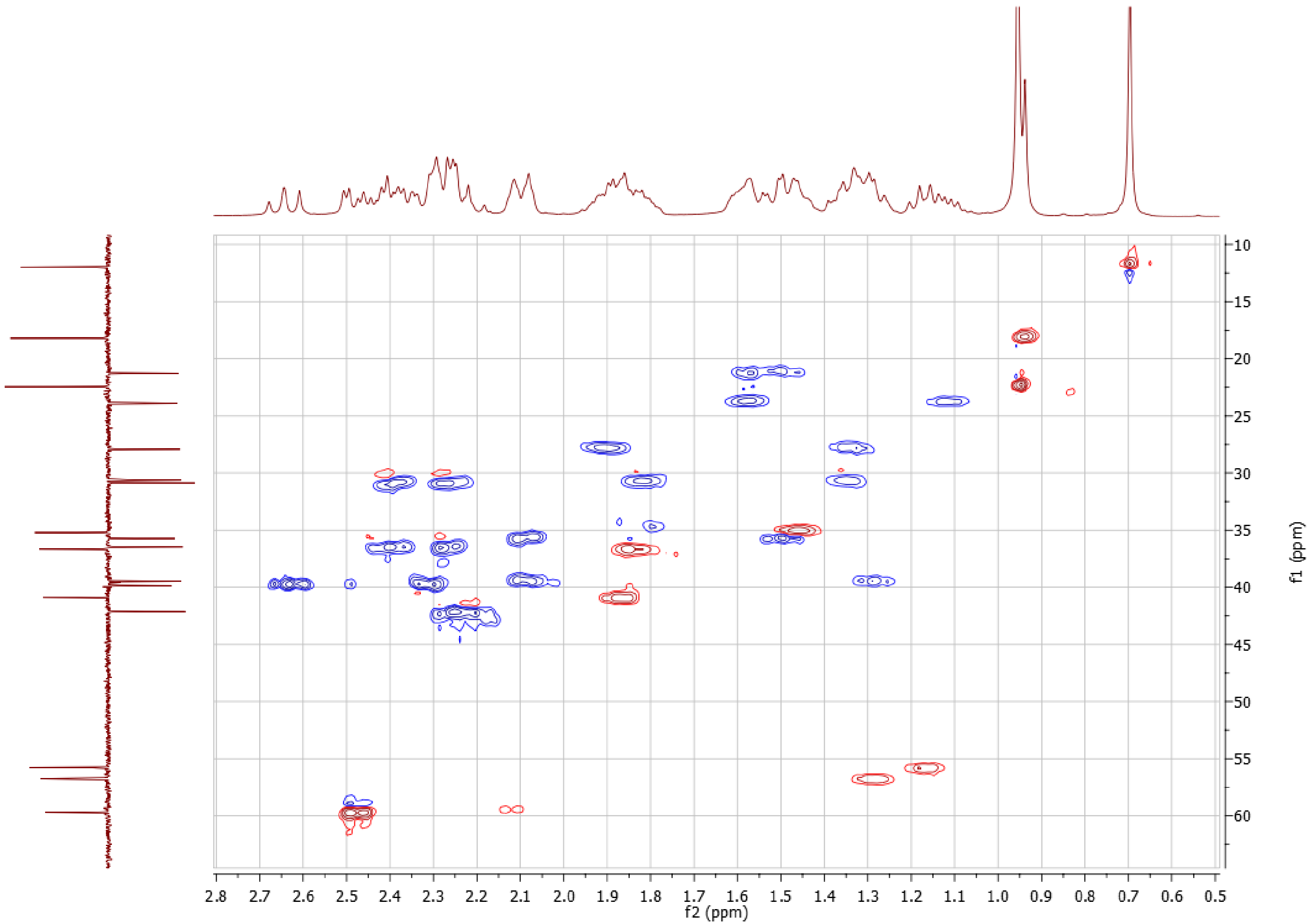

S4. 2D HSQC spectrum of *3,6-dioxo-5β-cholan-24-oic acid* (**42**)

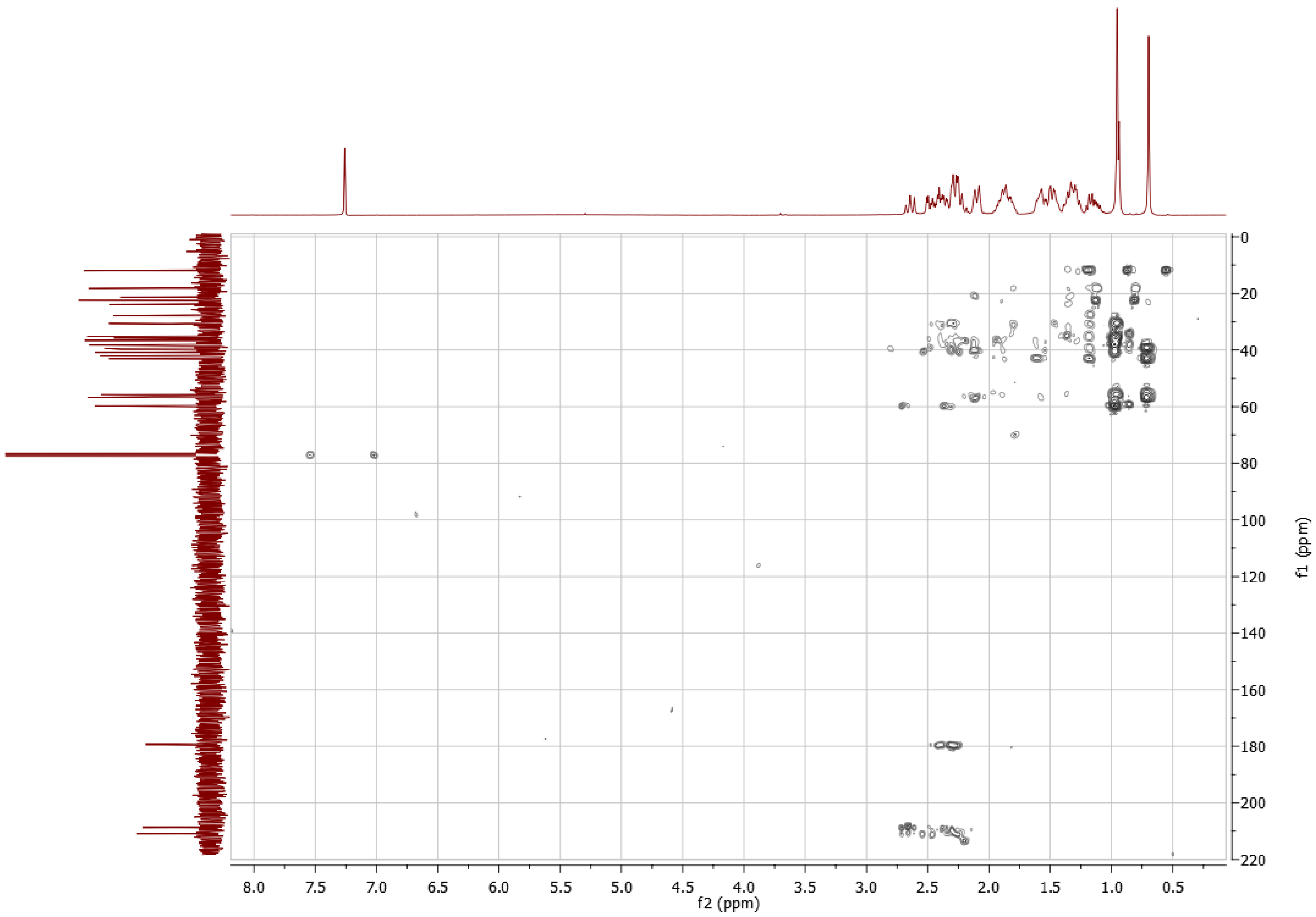

S5. 2D HMBC spectrum of *3,6-dioxo-5β-cholan-24-oic acid* (**42**)

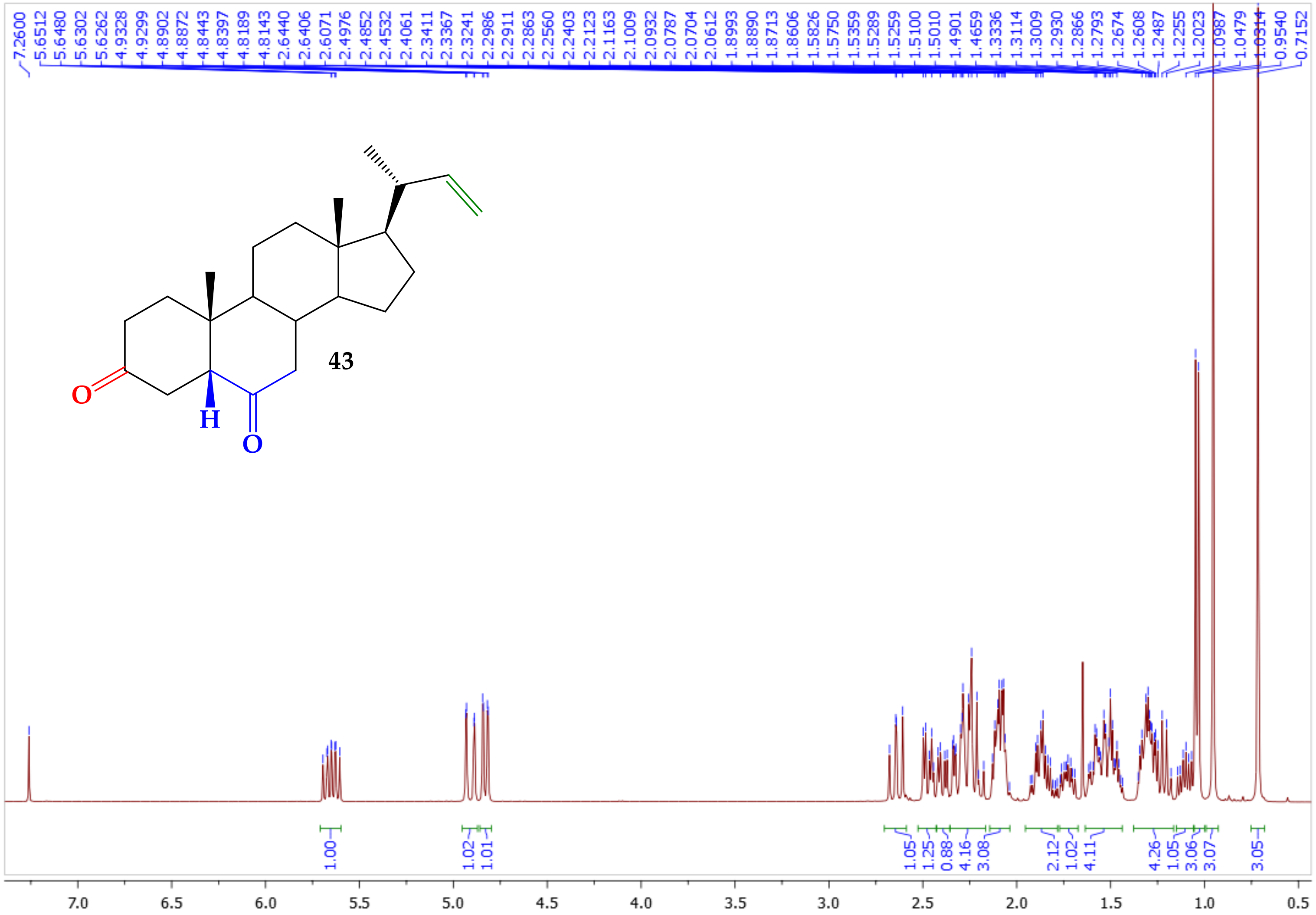

S6. ^{1}H NMR spectrum of *24-nor-5β-chol-22-ene-3,6-dione* (**43**)

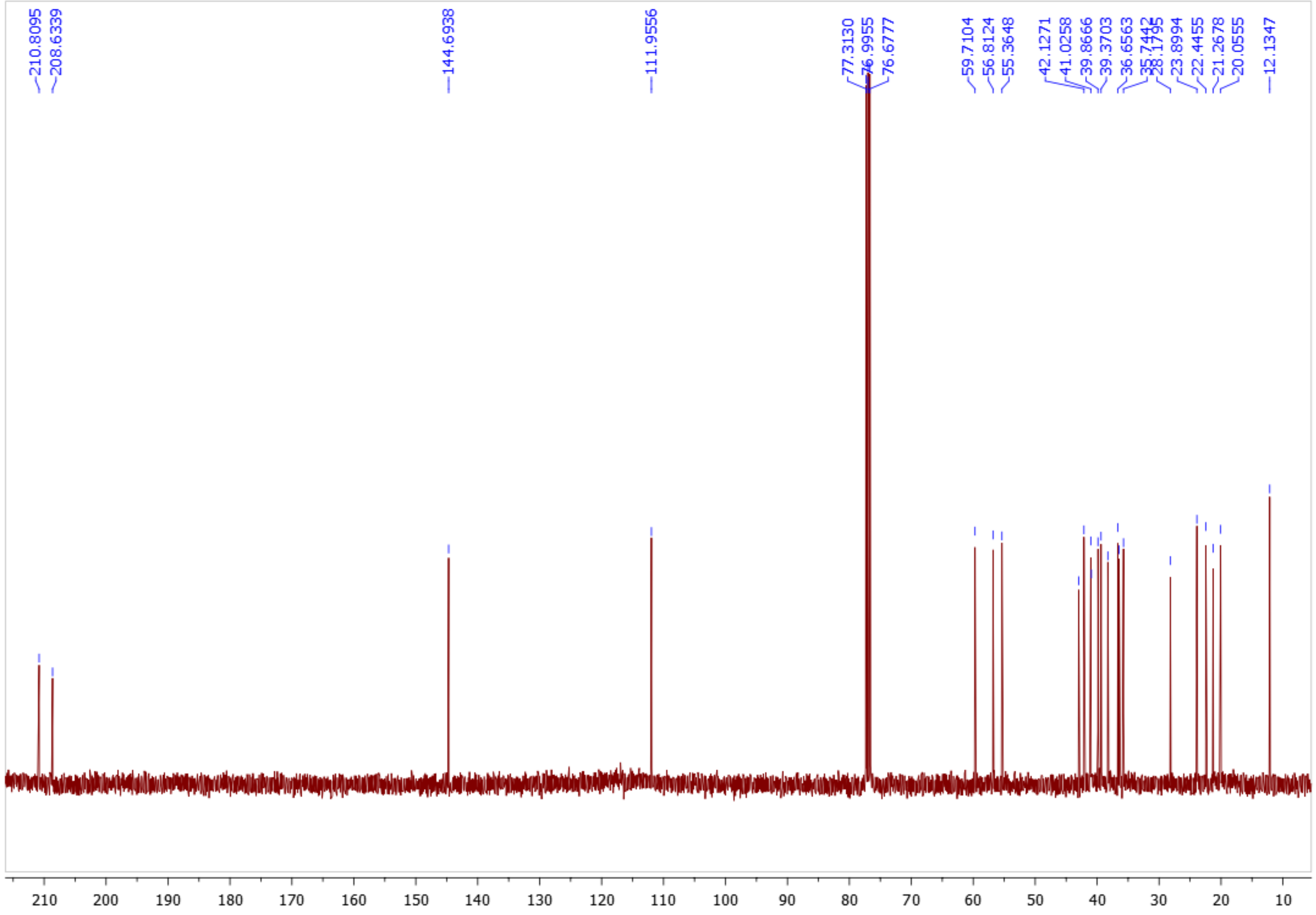

S7. ^{13}C NMR spectrum of *24-nor-5β-chol-22-ene-3,6-dione* (**43**)

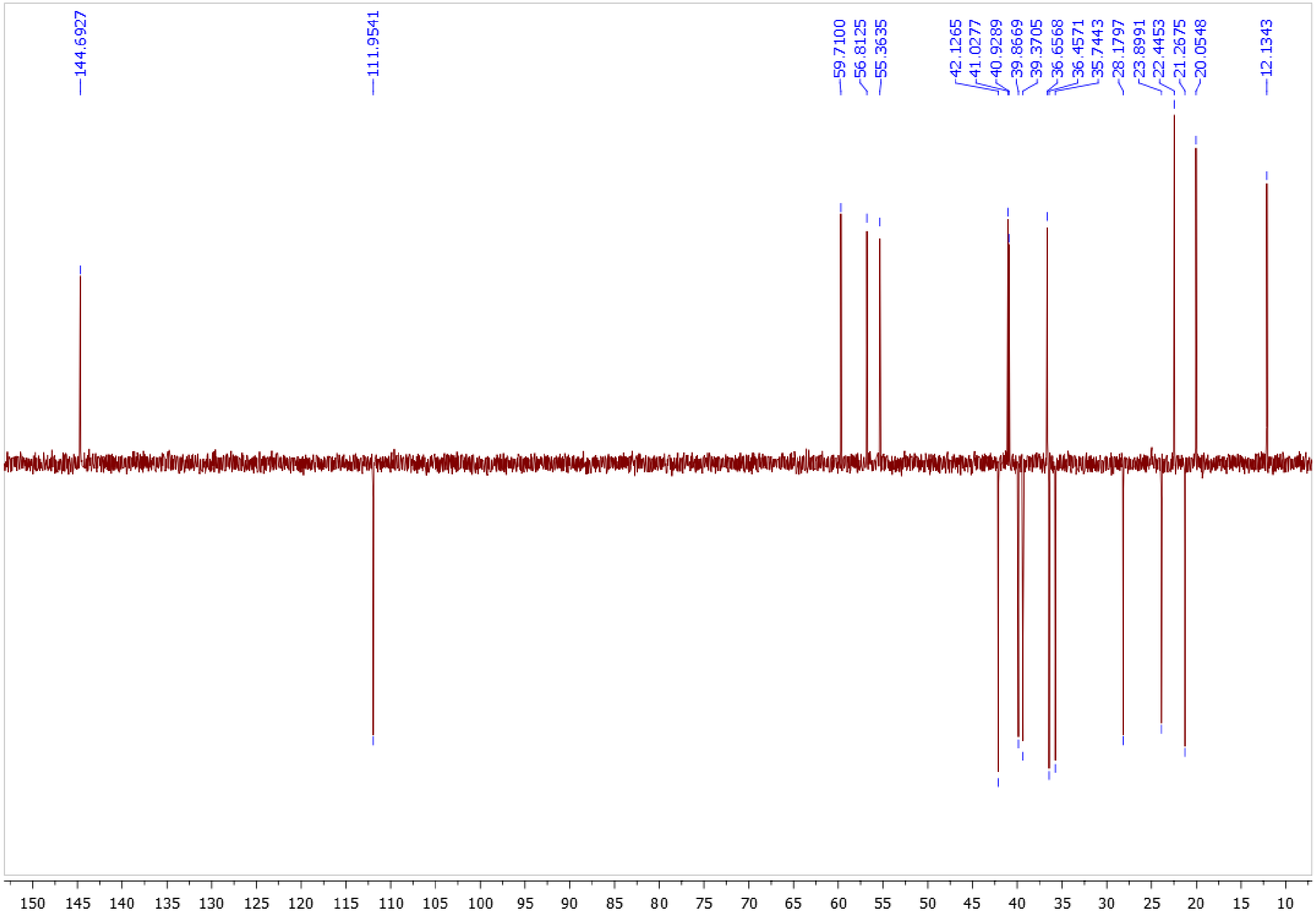

S8. ^{13}*C* DEPT-135 NMR spectrum of *24-nor-5β-chol-22-ene-3,6-dione* (***43***)

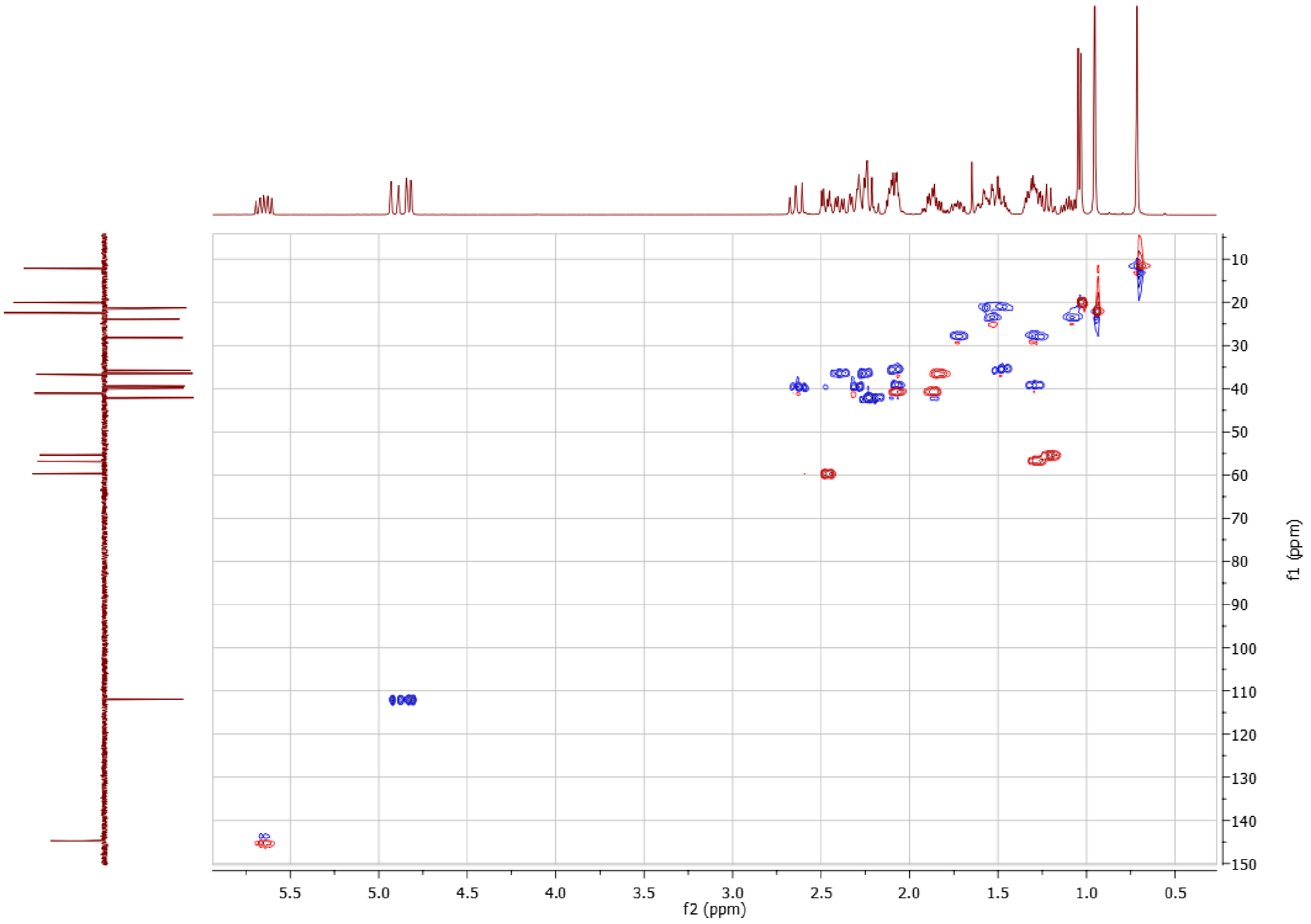

S9. 2D HSQC spectrum of *24-nor-5β-chol-22-ene-3,6-dione* (**43**)

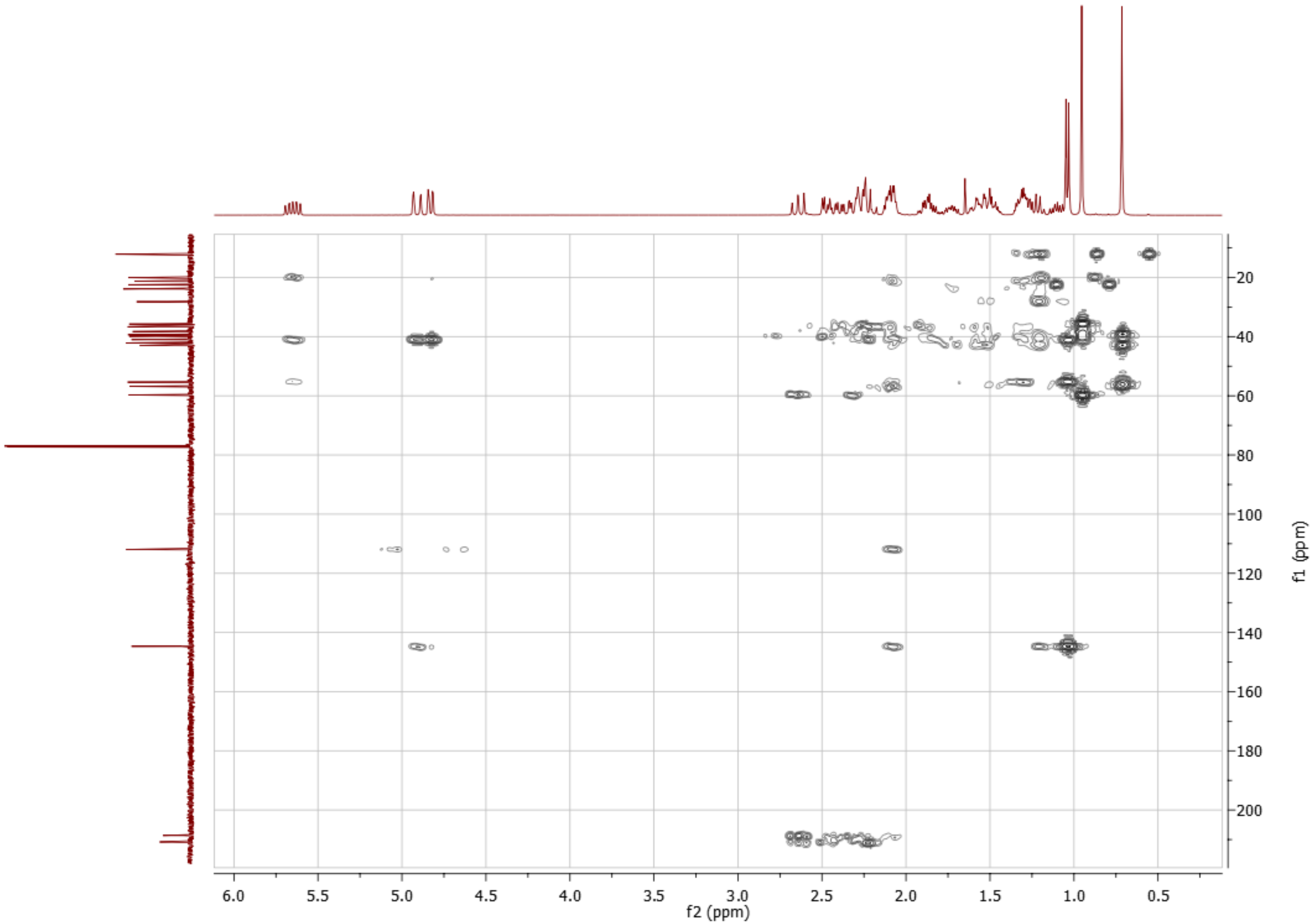

S10. 2D HMBC spectrum of *24-nor-5β-chol-22-ene-3,6-dione* (**43**)

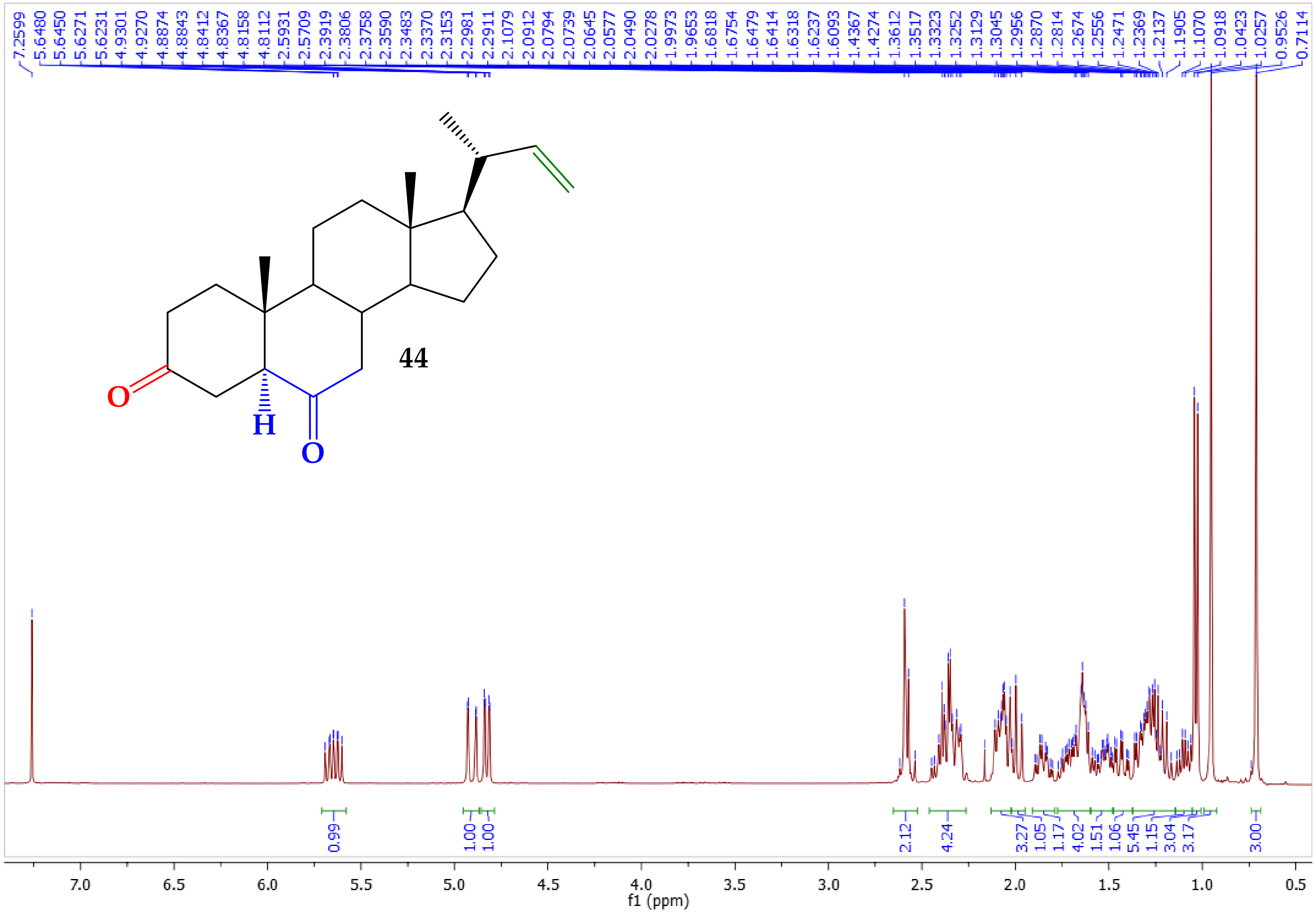

S11. ^{1}H NMR spectrum of *24-nor-5α-chol-22-ene-3,6-dione* (***44***)

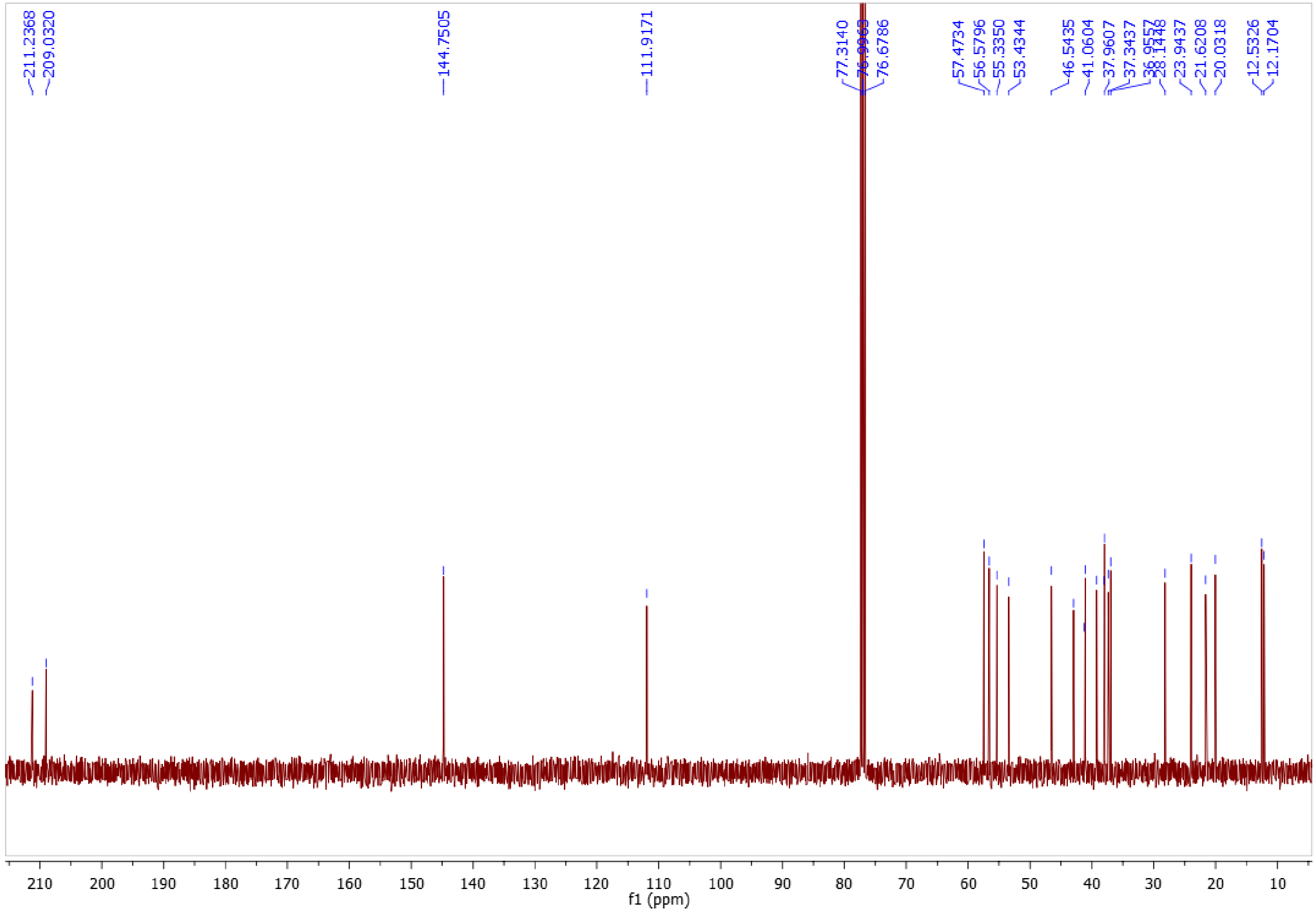

S12. ^{13}C NMR spectrum of *24-nor-5α-chol-22-ene-3,6-dione* (***44***)

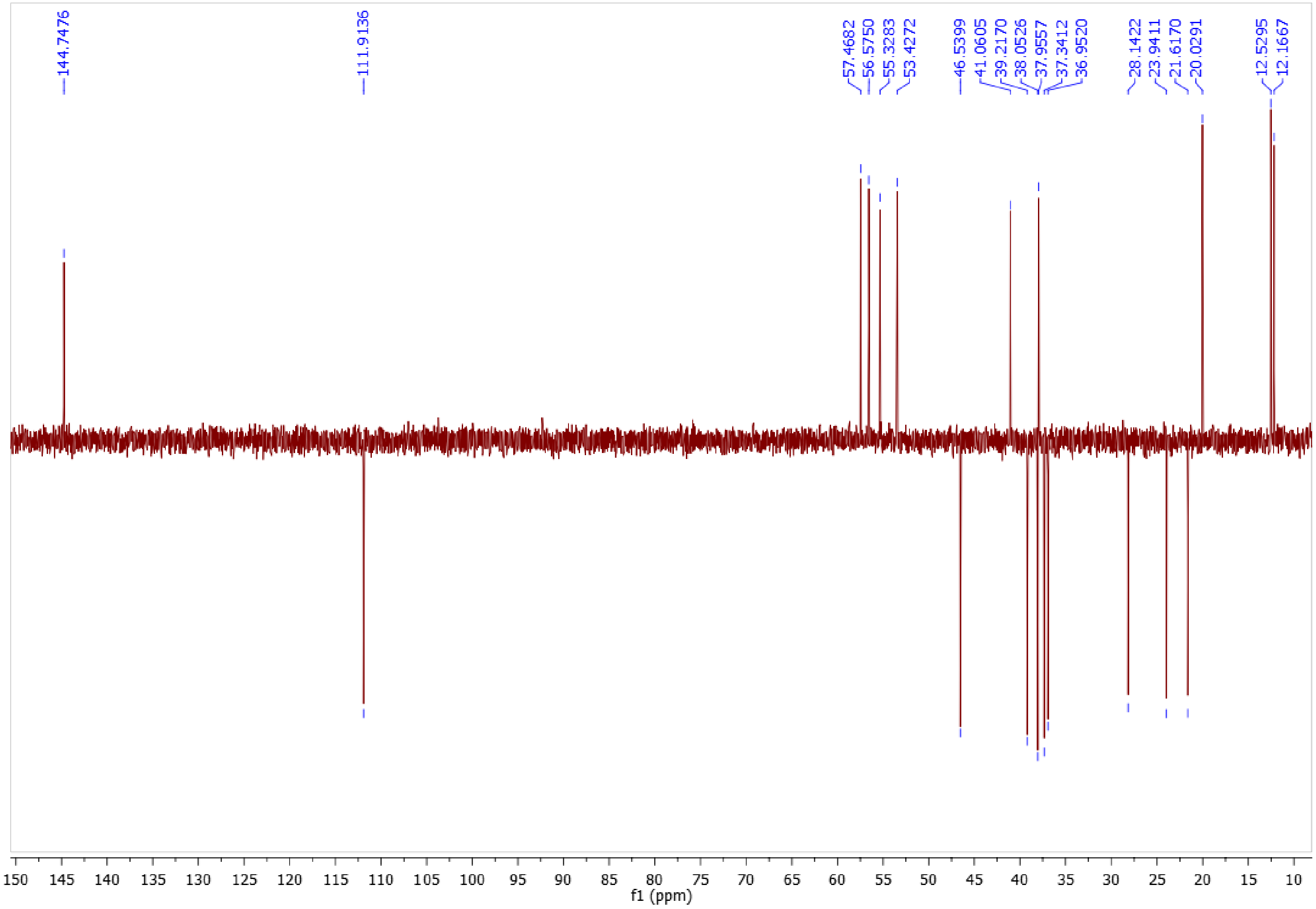

S13. ^{13}C DEPT-135 NMR spectrum of *24-nor-5α-chol-22-ene-3,6-dione* (***44***)

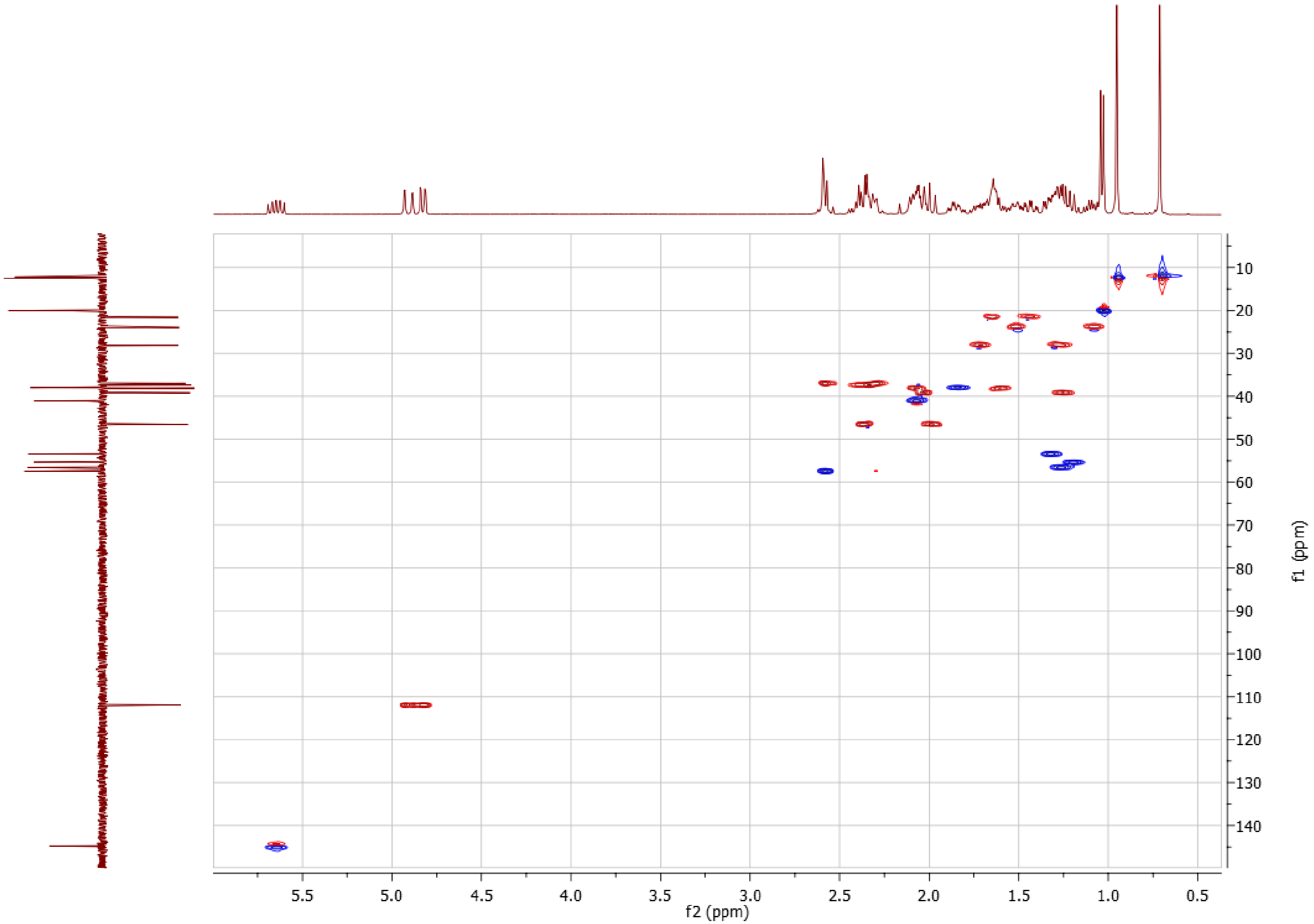

S14. 2D HSQC NMR spectrum of *24-nor-5α-chol-22-ene-3,6-dione* (***44***)

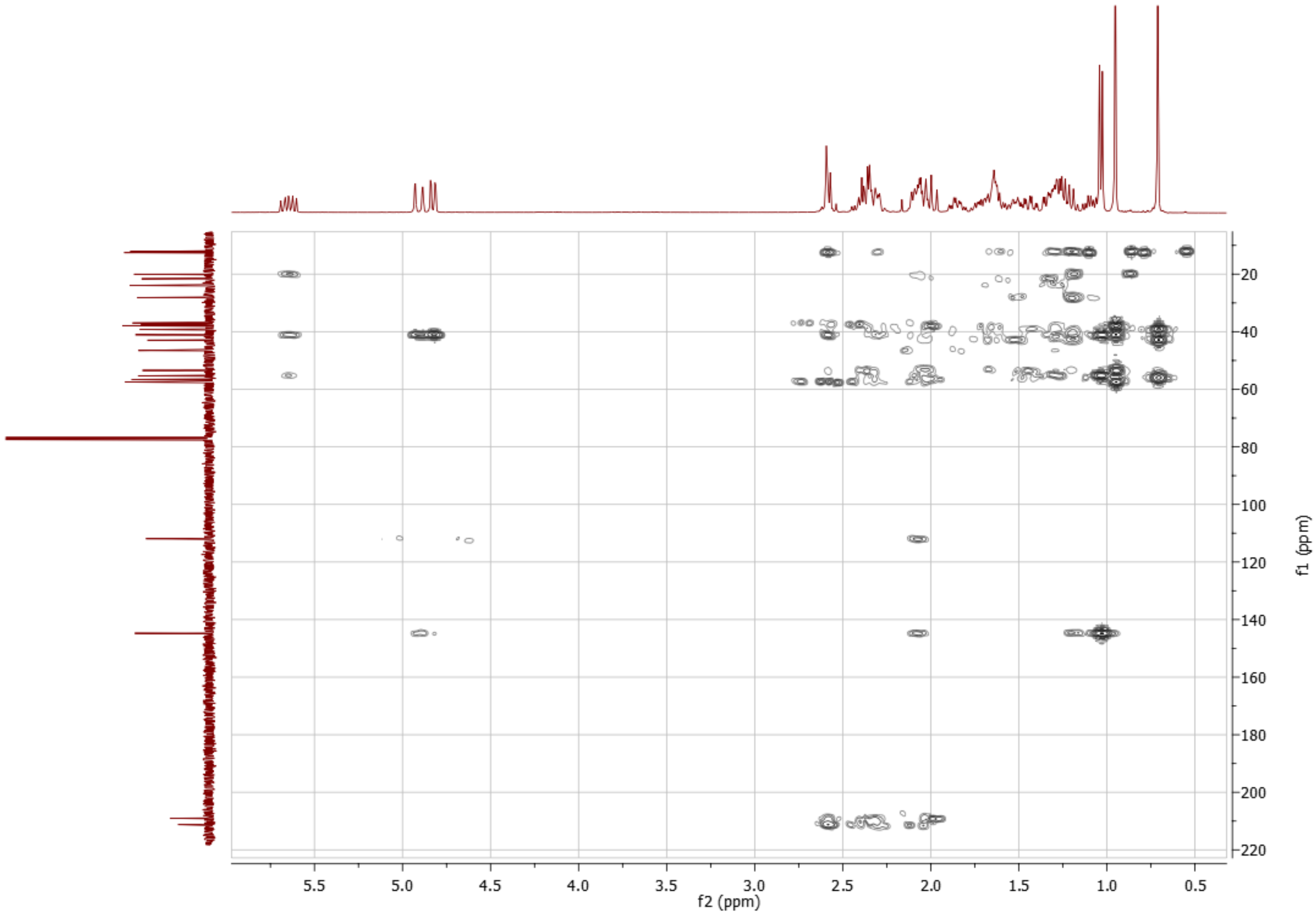

S15. 2D HMBC NMR spectrum of *24-nor-5α-chol-22-ene-3,6-dione* (***44***)

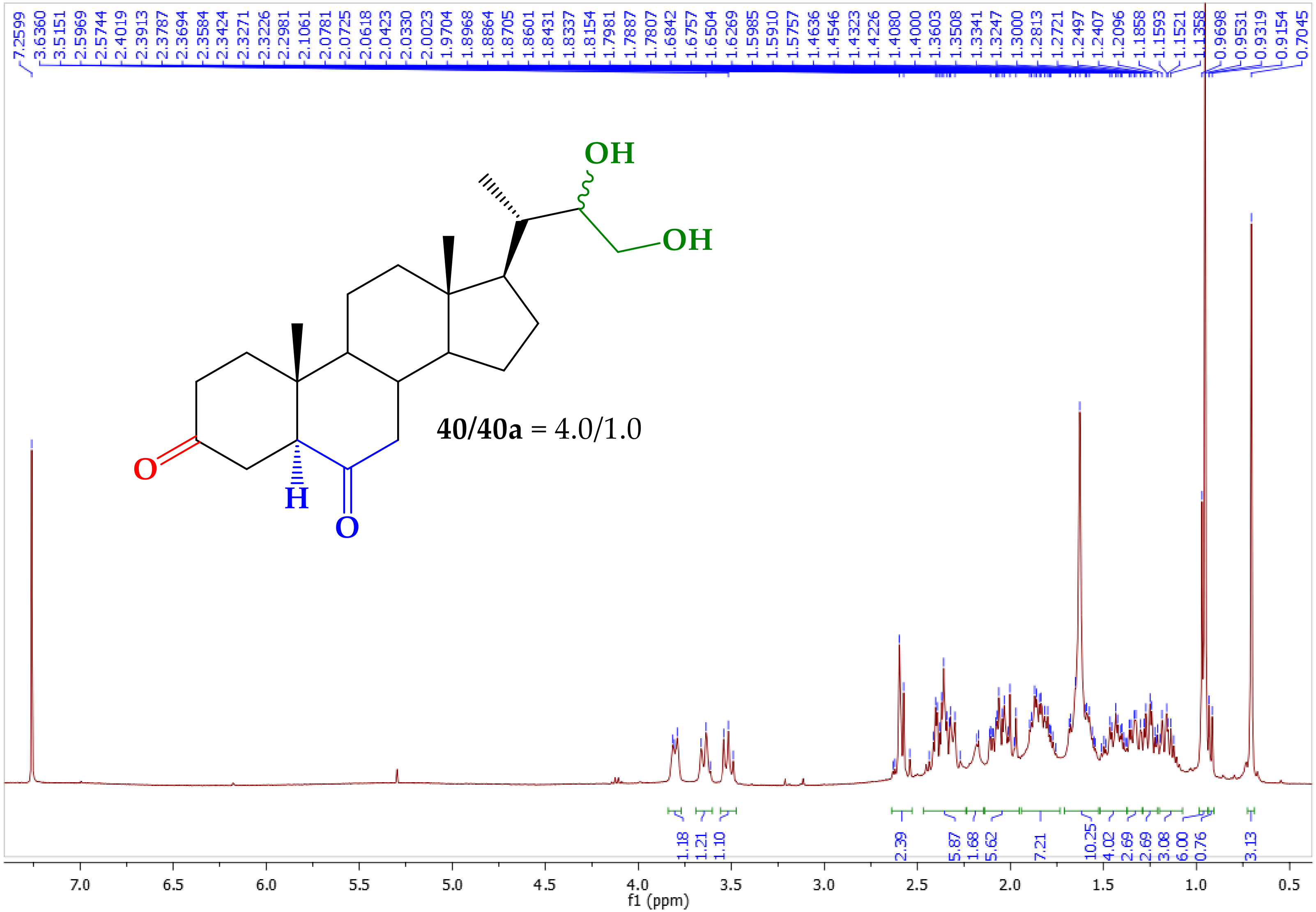

S16. ^{1}H NMR spectrum of *22(S), 23-dihidroxy-24-nor-5α-cholan-3,6-dione* (**40**) and *22(R), 23-dihidroxy-24-nor-5α-cholan-3,6-dione* (***40a***)

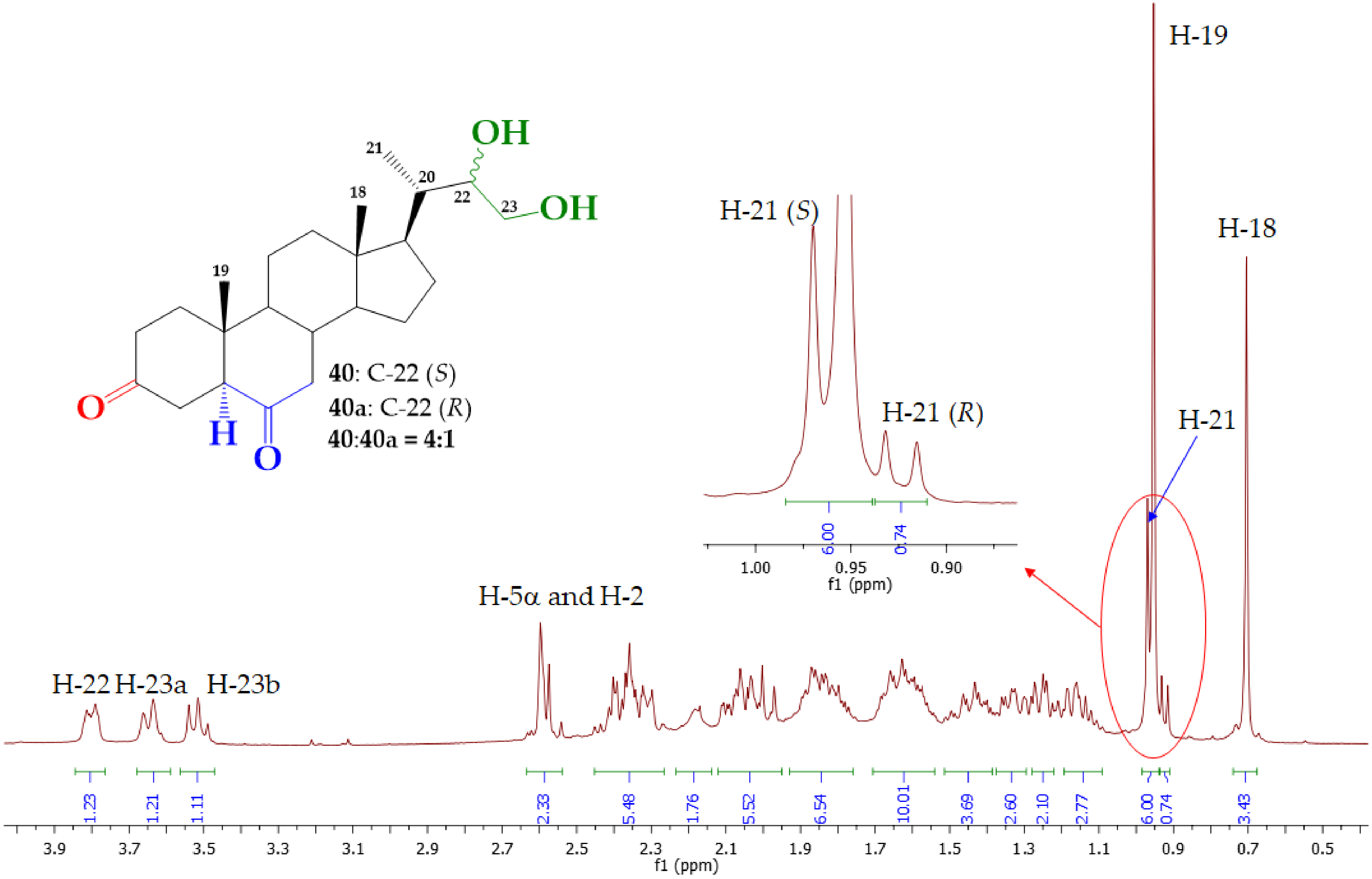

S17. ^{1}H NMR spectrum expansion and proportion determination for ***40**:**40a*** = *4:1* epimers mixture.

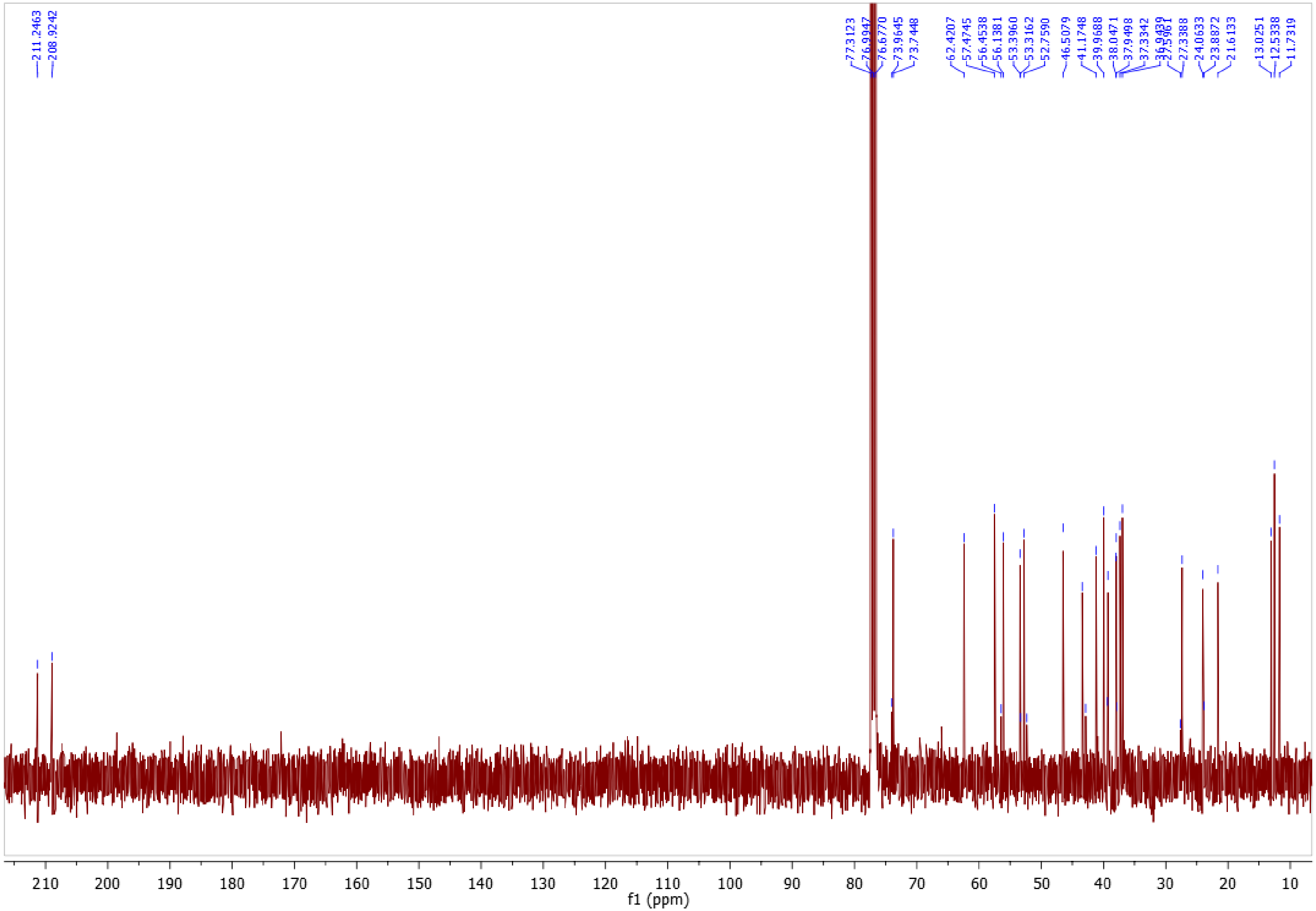

S18. ^{13}C NMR spectrum of *22(S), 23-dihidroxy-24-nor-5α-cholan-3,6-dione* (***40***) and *22(R), 23-dihidroxy-24-nor-5α-cholan-3,6-dione* (***40a***)

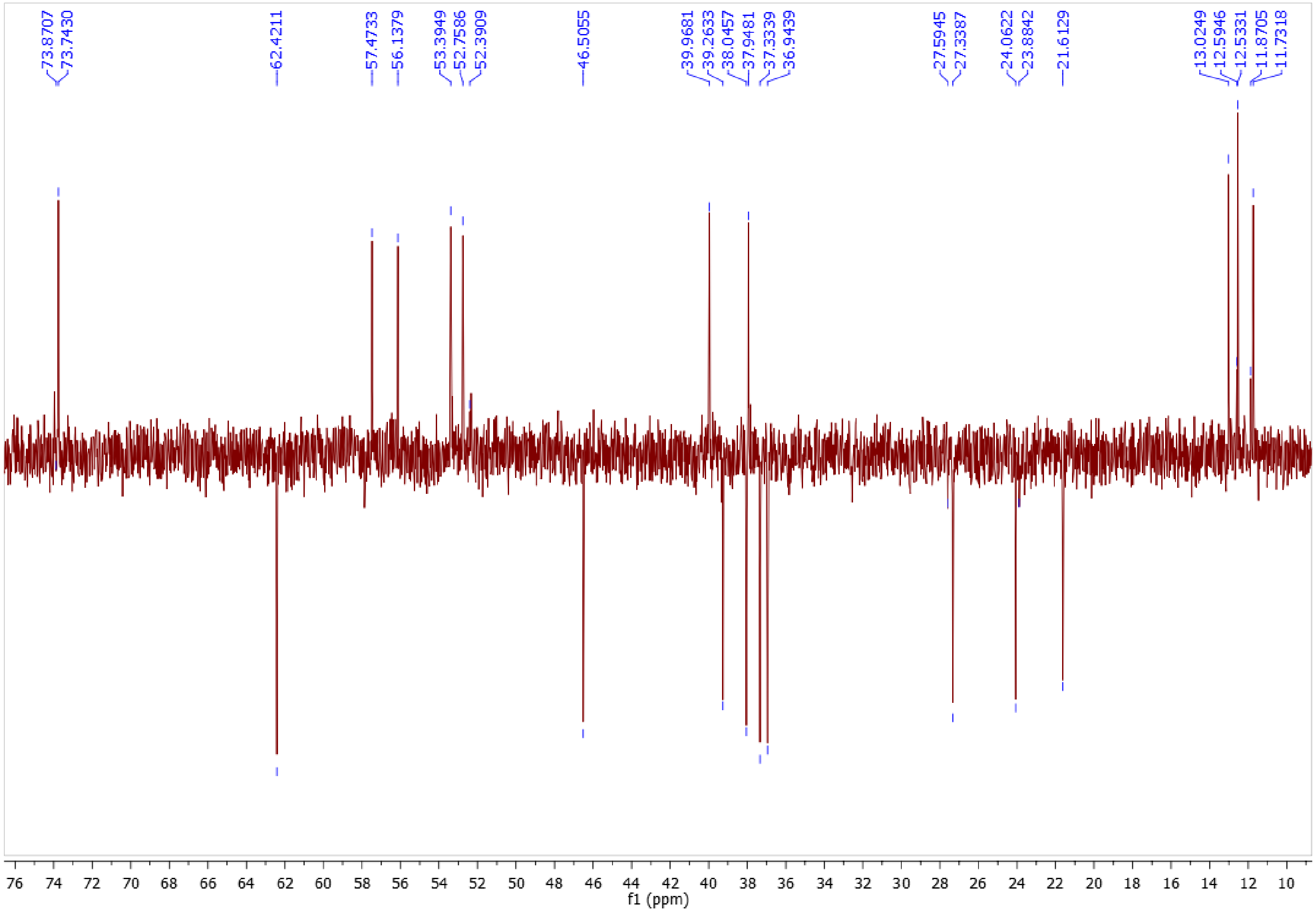

S19. ^{13}C DEPT-135 NMR spectrum of *22(S), 23-dihidroxy-24-nor-5α-cholan-3,6-dione* (**40**) and *22(R), 23-dihidroxy-24-nor-5α-cholan-3,6-dione (**40a**)*

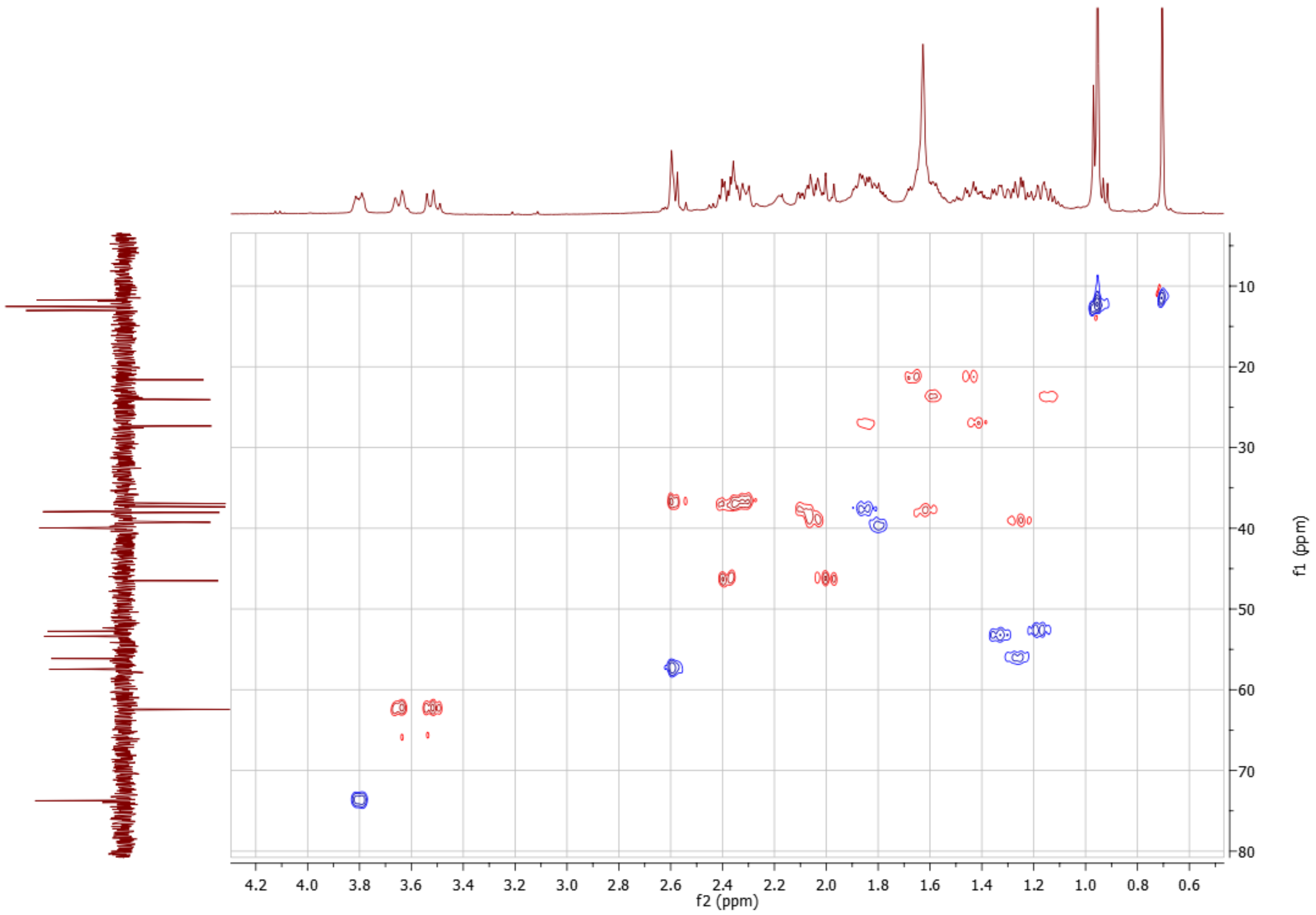

S20. 2D HSQC NMR spectrum of *22(S), 23-dihidroxy-24-nor-5α-cholan-3,6-dione* (**40**) and *22(R), 23-dihidroxy-24-nor-5α-cholan-3,6-dione* (***40a***)

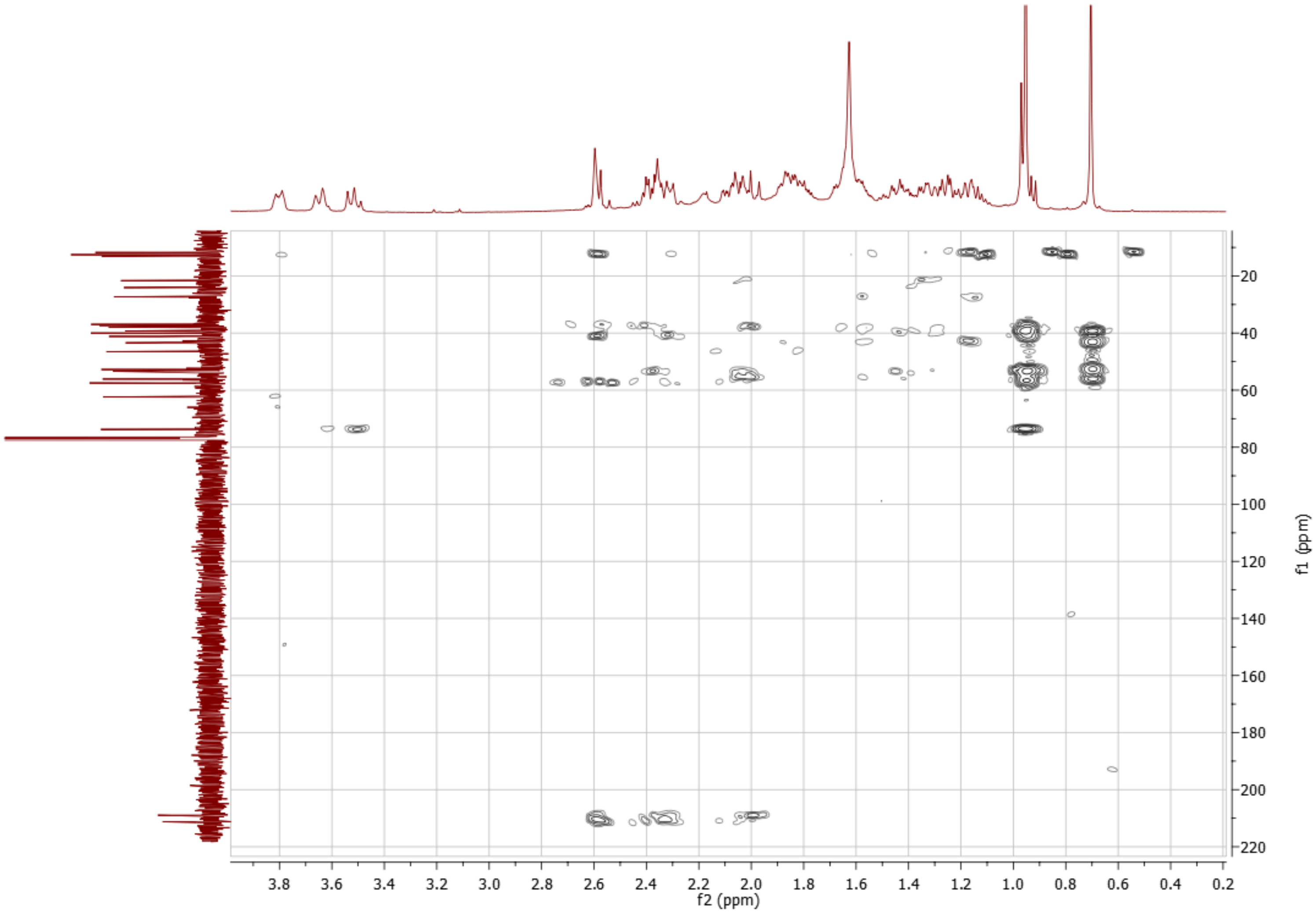

S21. 2D HMBC NMR spectrum of *22(S), 23-dihidroxy-24-nor-5α-cholan-3,6-dione* (**40**) and *22(R), 23-dihidroxy-24-nor-5α-cholan-3,6-dione* (***40a***)

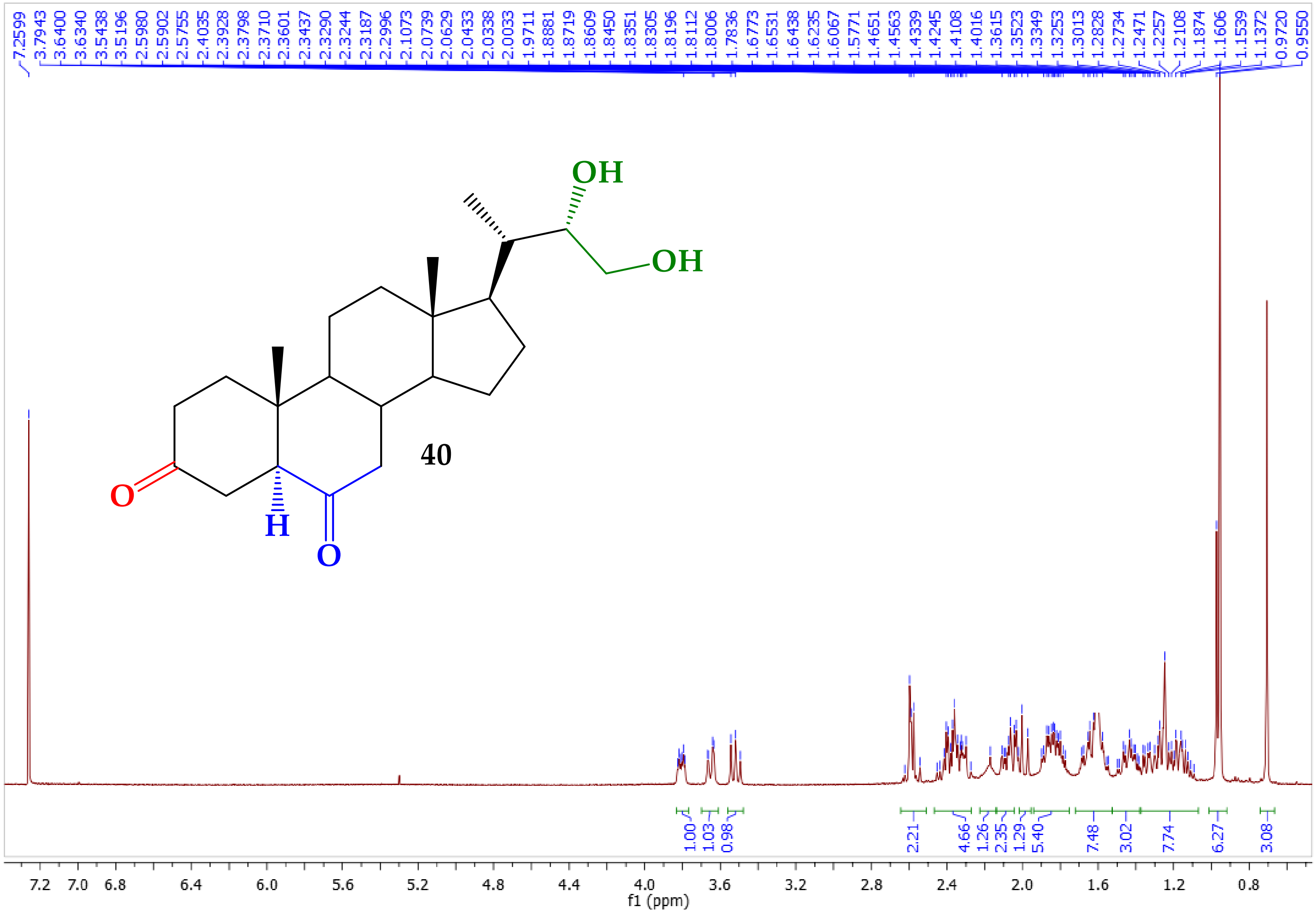

S22. ^{1}H NMR spectrum of *22(S), 23-dihidroxy-24-nor-5α-cholan-3,6-dione* (**40**)

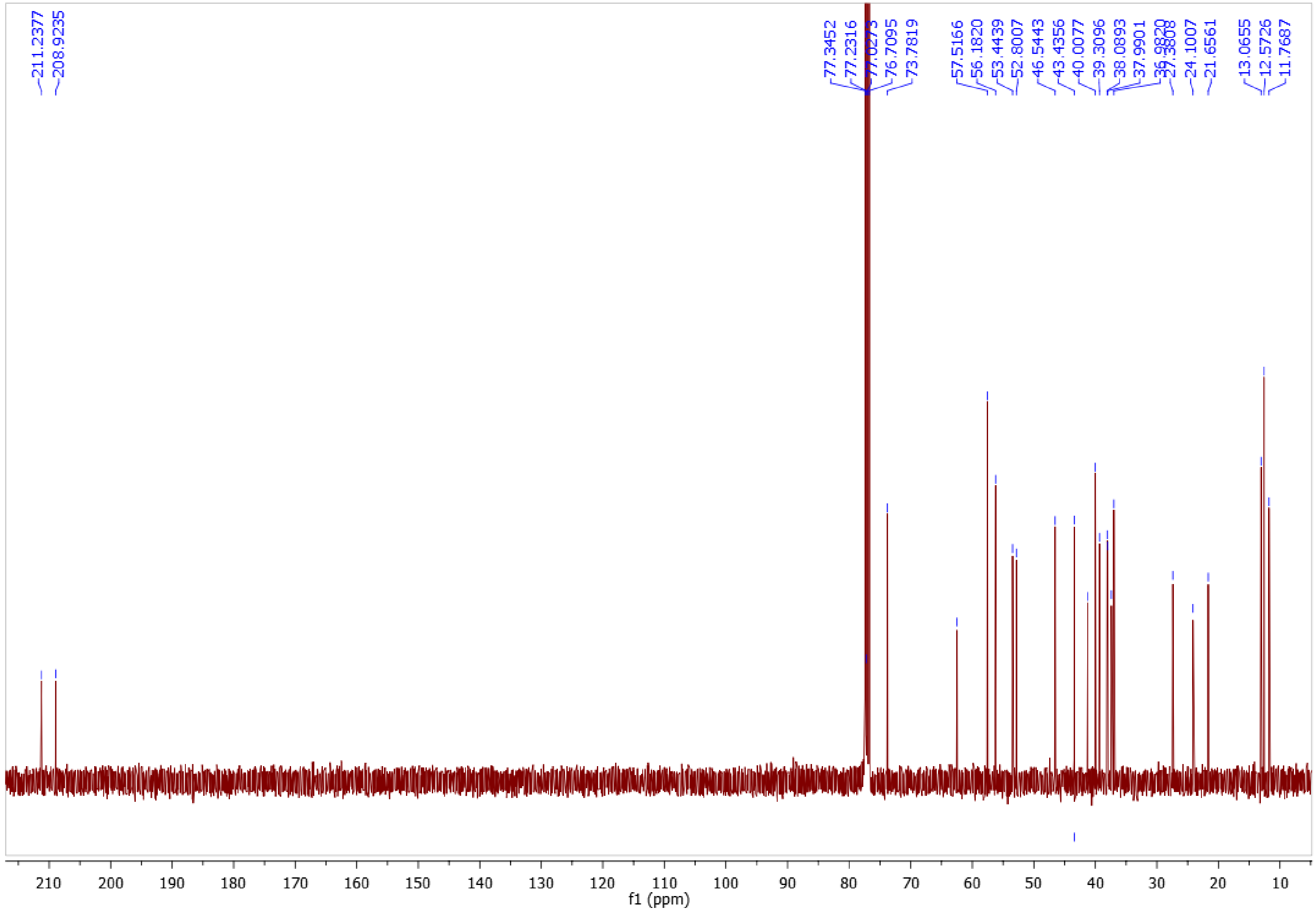

S23. ^{13}C NMR spectrum of *22(S), 23-dihidroxy-24-nor-5α-cholan-3,6-dione* (**40**)

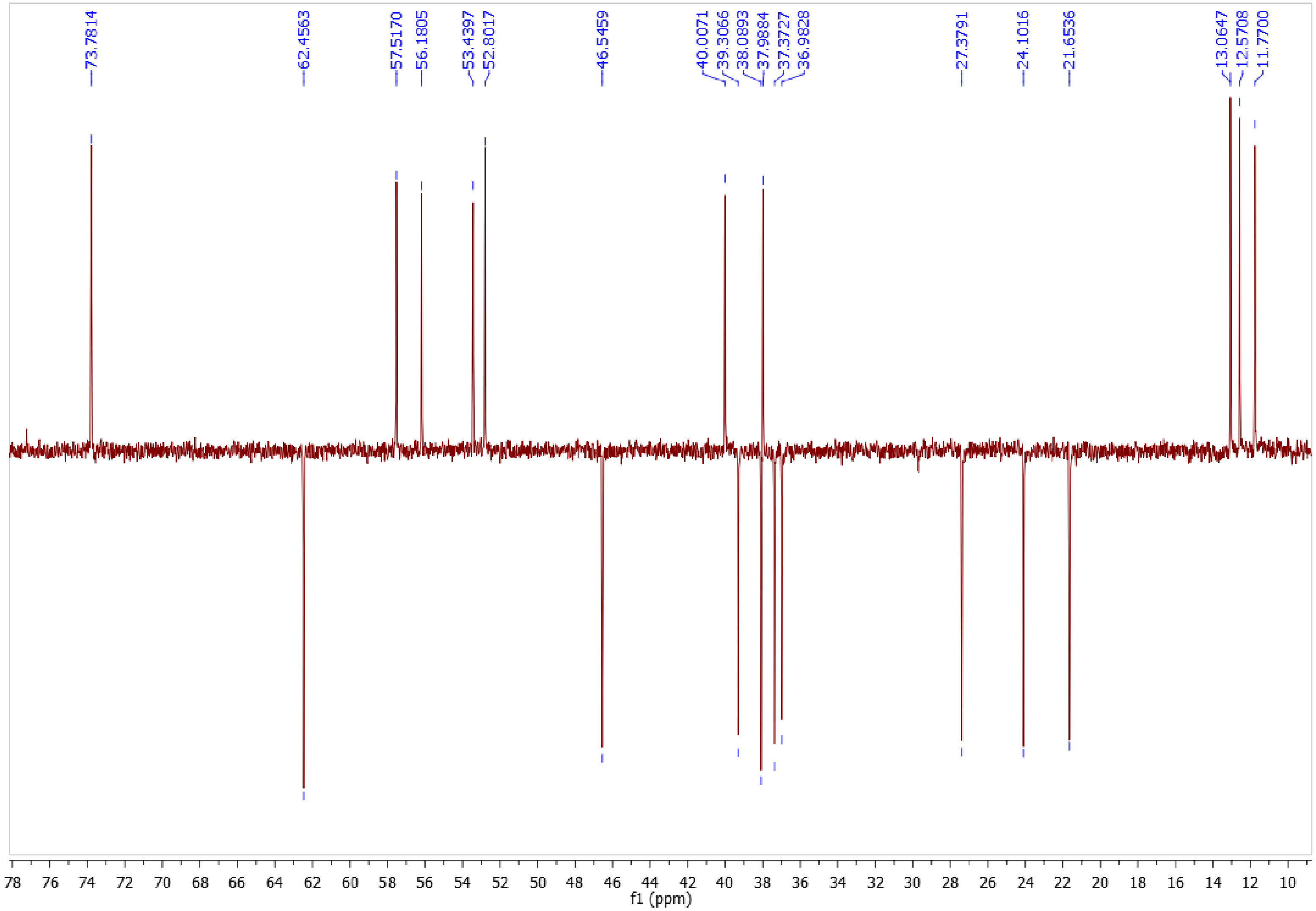

S24. ^{13}C DEPT-135 NMR spectrum of *22(S), 23-dihidroxy-24-nor-5α-cholan-3,6-dione* (**40**)

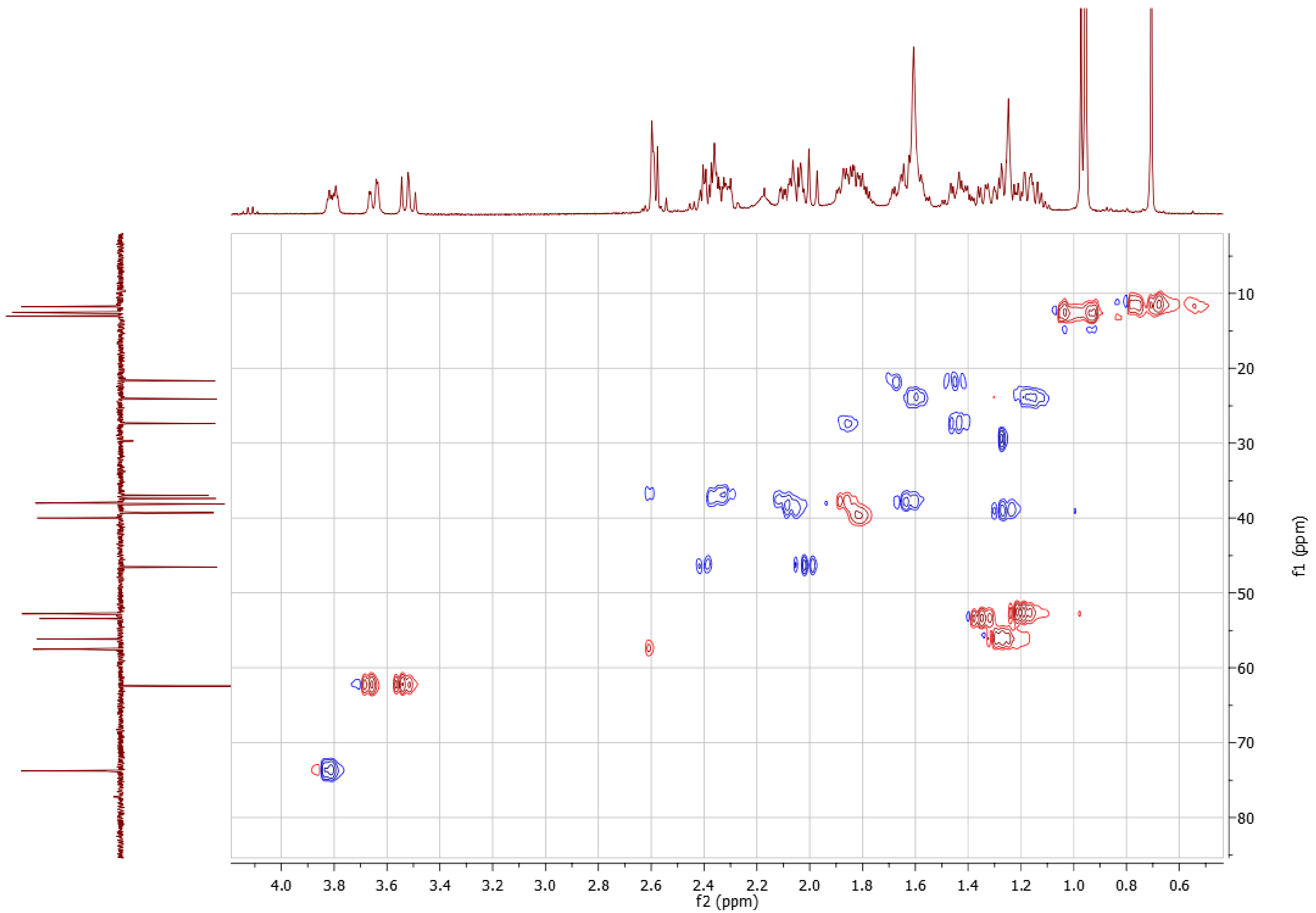

S25. 2D HSQC NMR spectrum of *22(S), 23-dihidroxy-24-nor-5α-cholan-3,6-dione* (***40***)

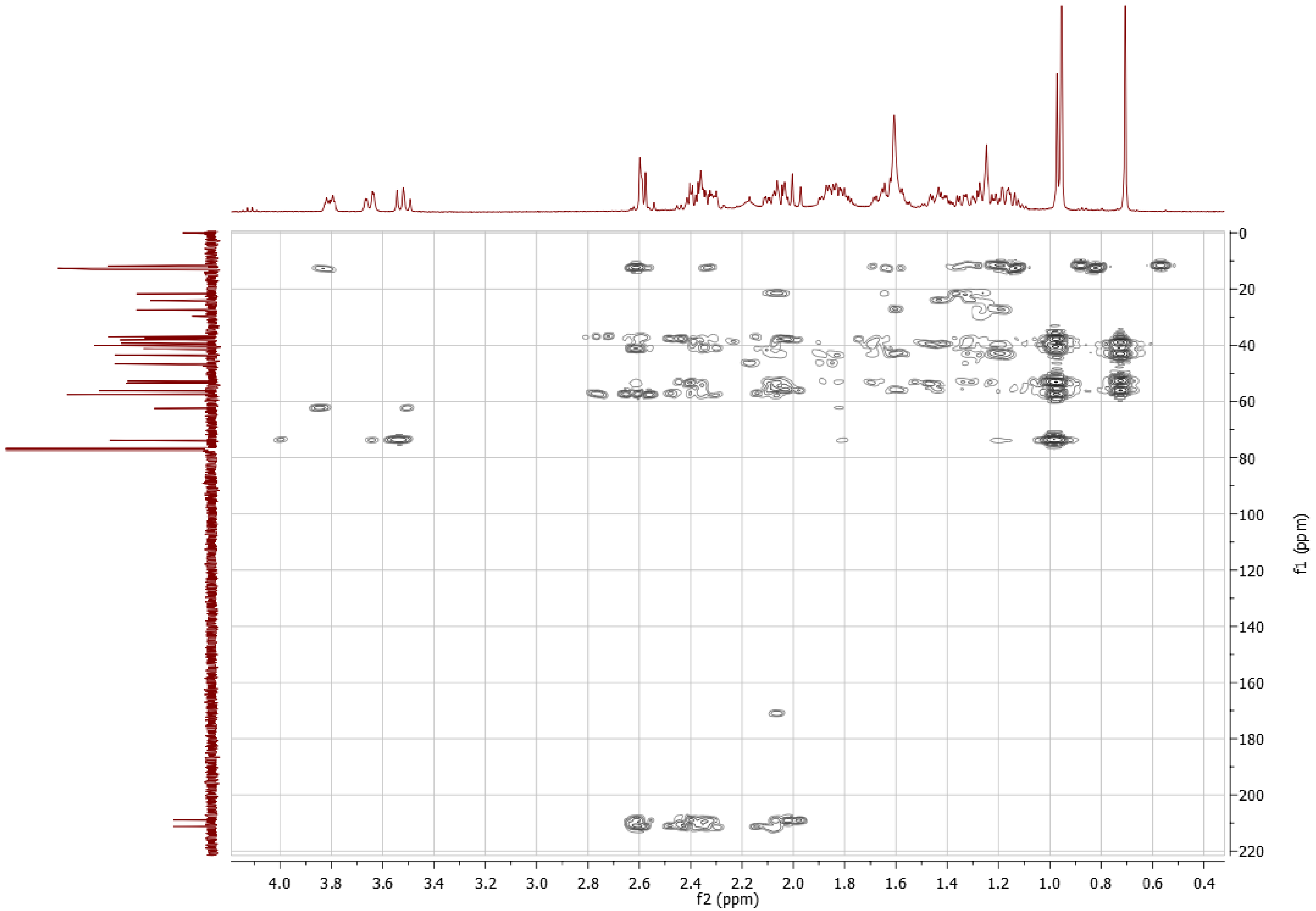

S26. 2D HMBC NMR spectrum of *22(S), 23-dihidroxy-24-nor-5α-cholan-3,6-dione* (**40**)

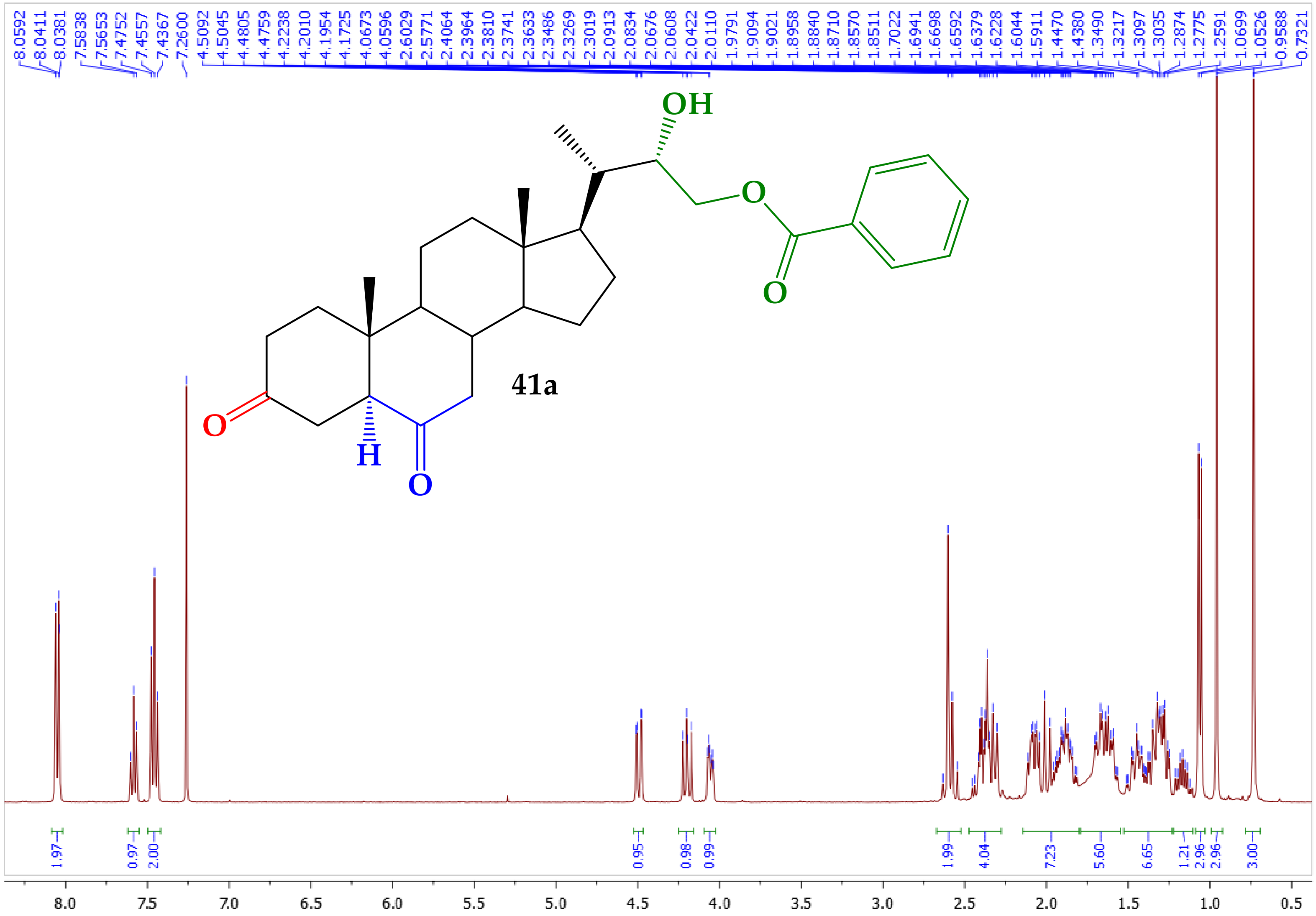

S27. ^{1}H NMR spectrum of *22(S)-hydroxy-24-nor-5α-cholan-3,6-dioxobenzoate-23-yl* (***41a***)

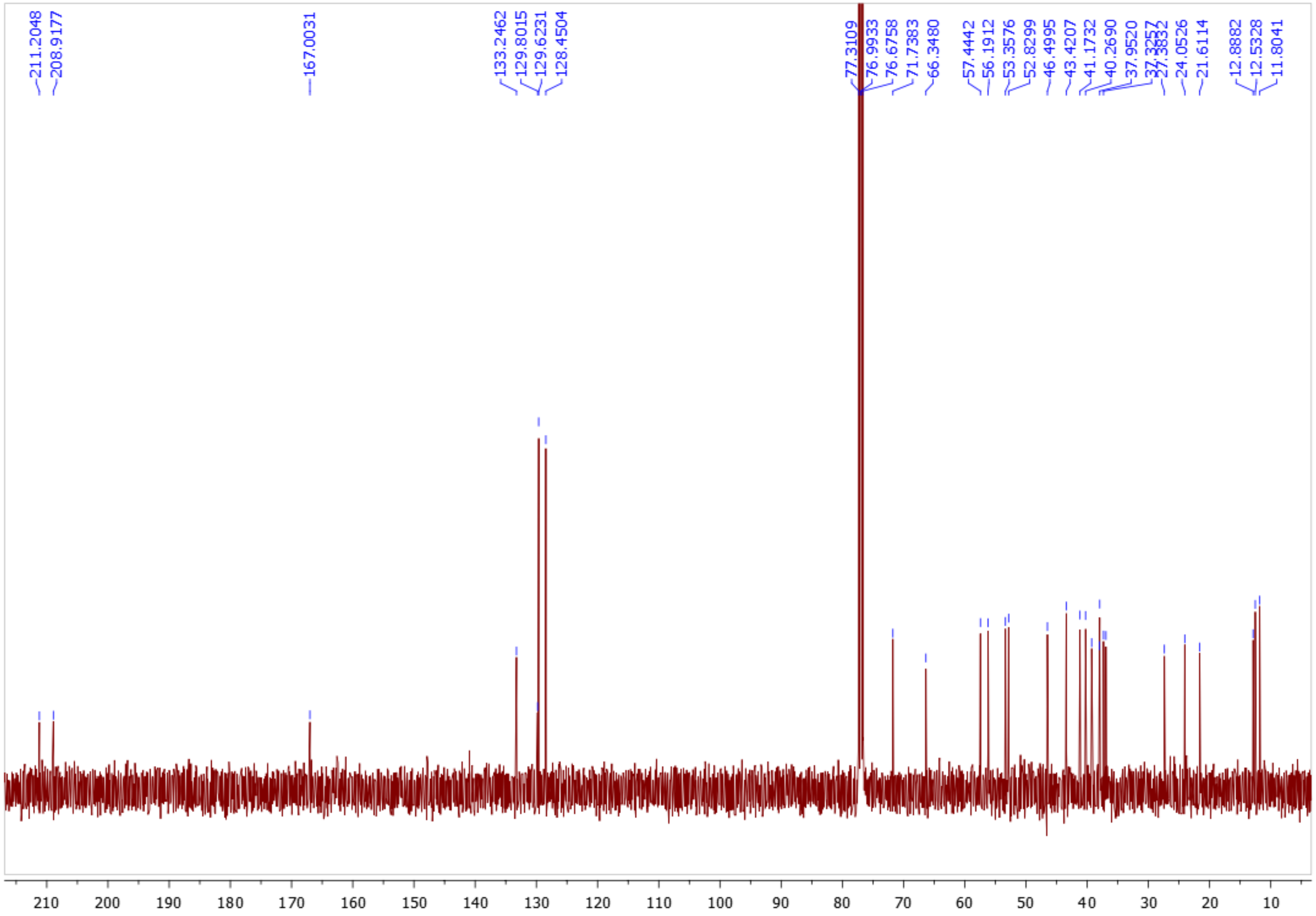

S28. ^{13}C NMR spectrum of *22(S)-hydroxy-24-nor-5α-cholan-3,6-dioxobenzoate-23-yl* (***41a***)

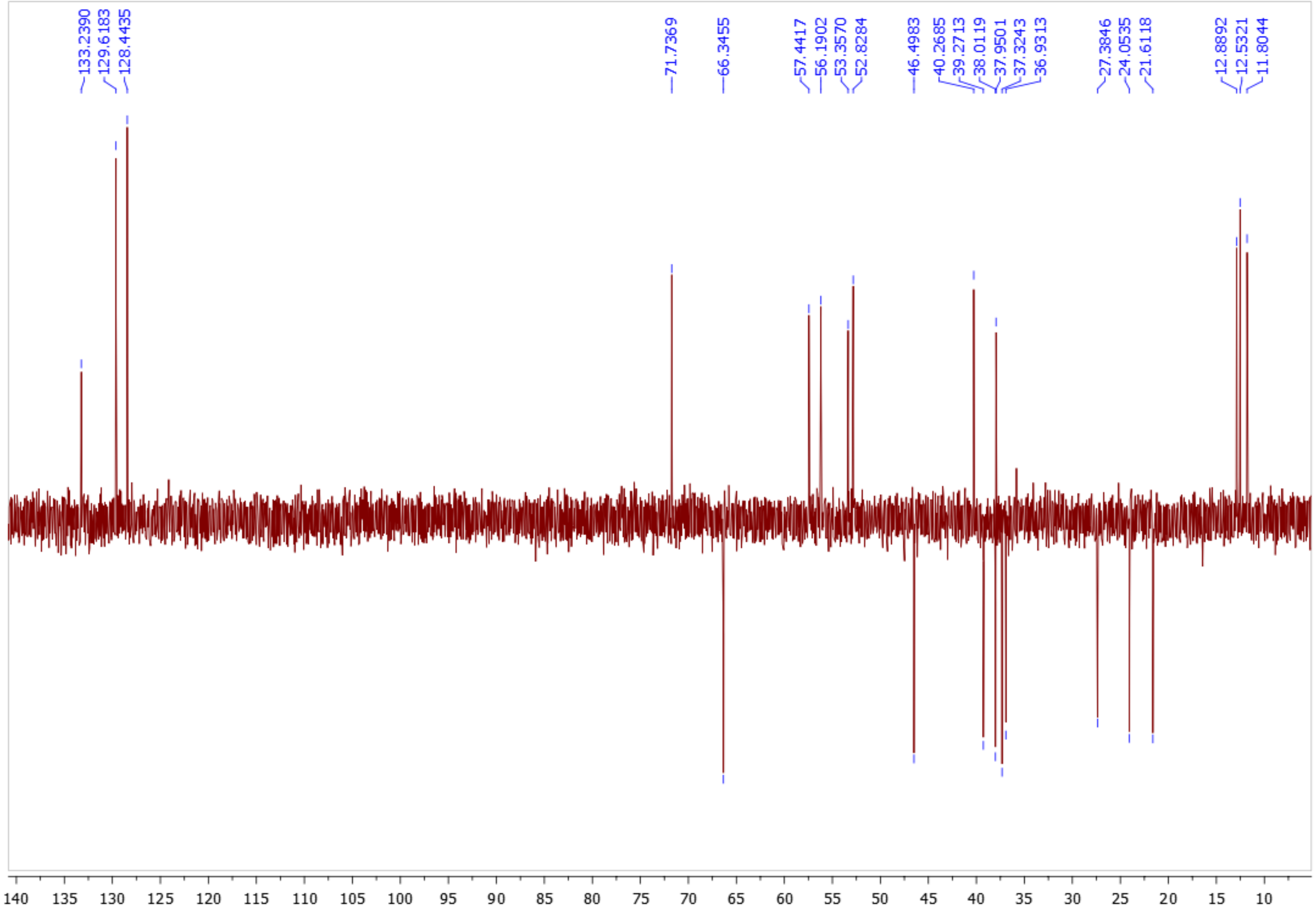

S29. ¹³C DEPT-135 NMR spectrum of *22(S)-hydroxy-24-nor-5α-cholan-3,6-dioxobenzoate-23-yl* (***41a***)

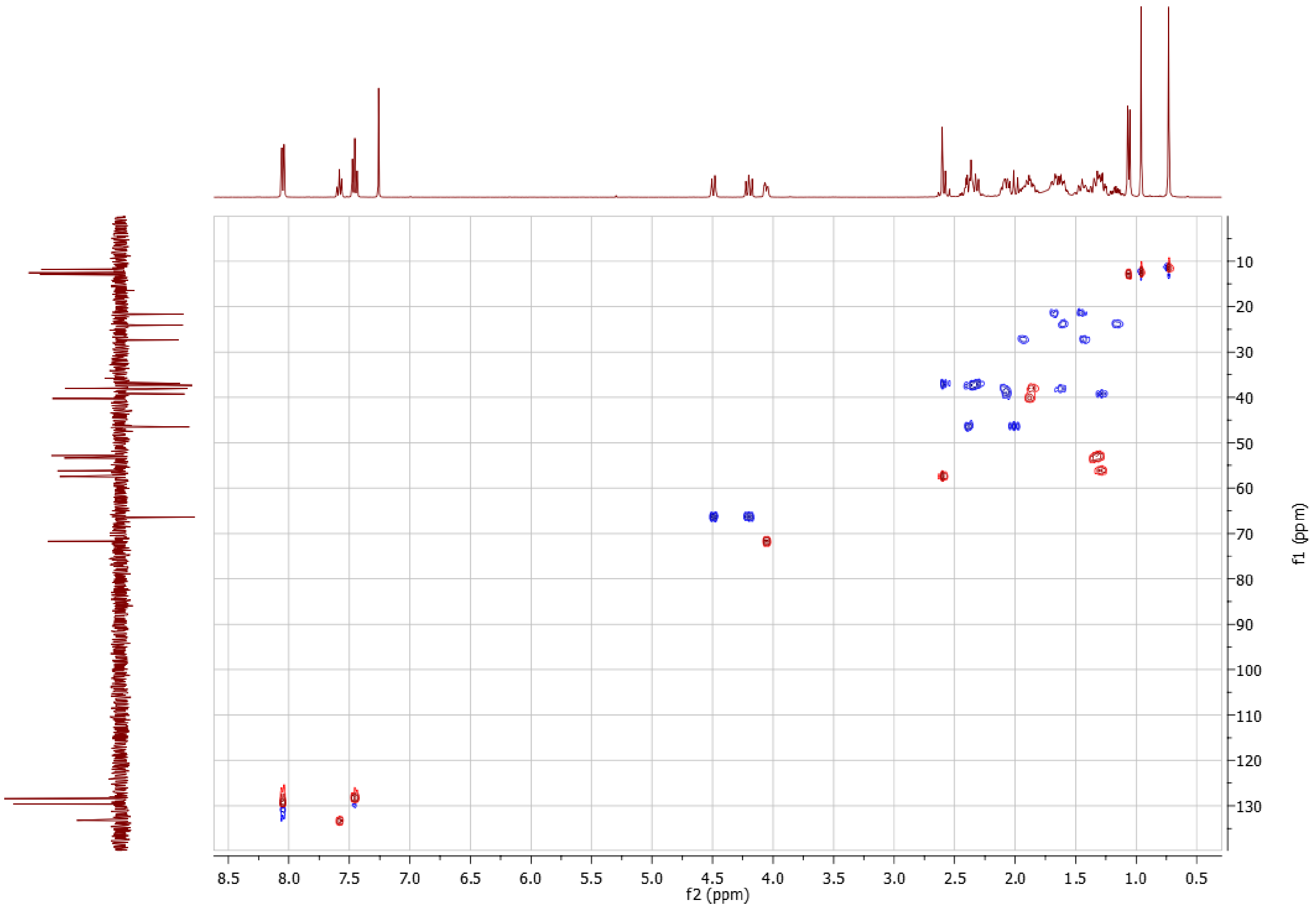

S30. 2D HSQC NMR spectrum of *22(S)-hydroxy-24-nor-5α-cholan-3,6-dioxobenzoate-23-yl* (***41a***)

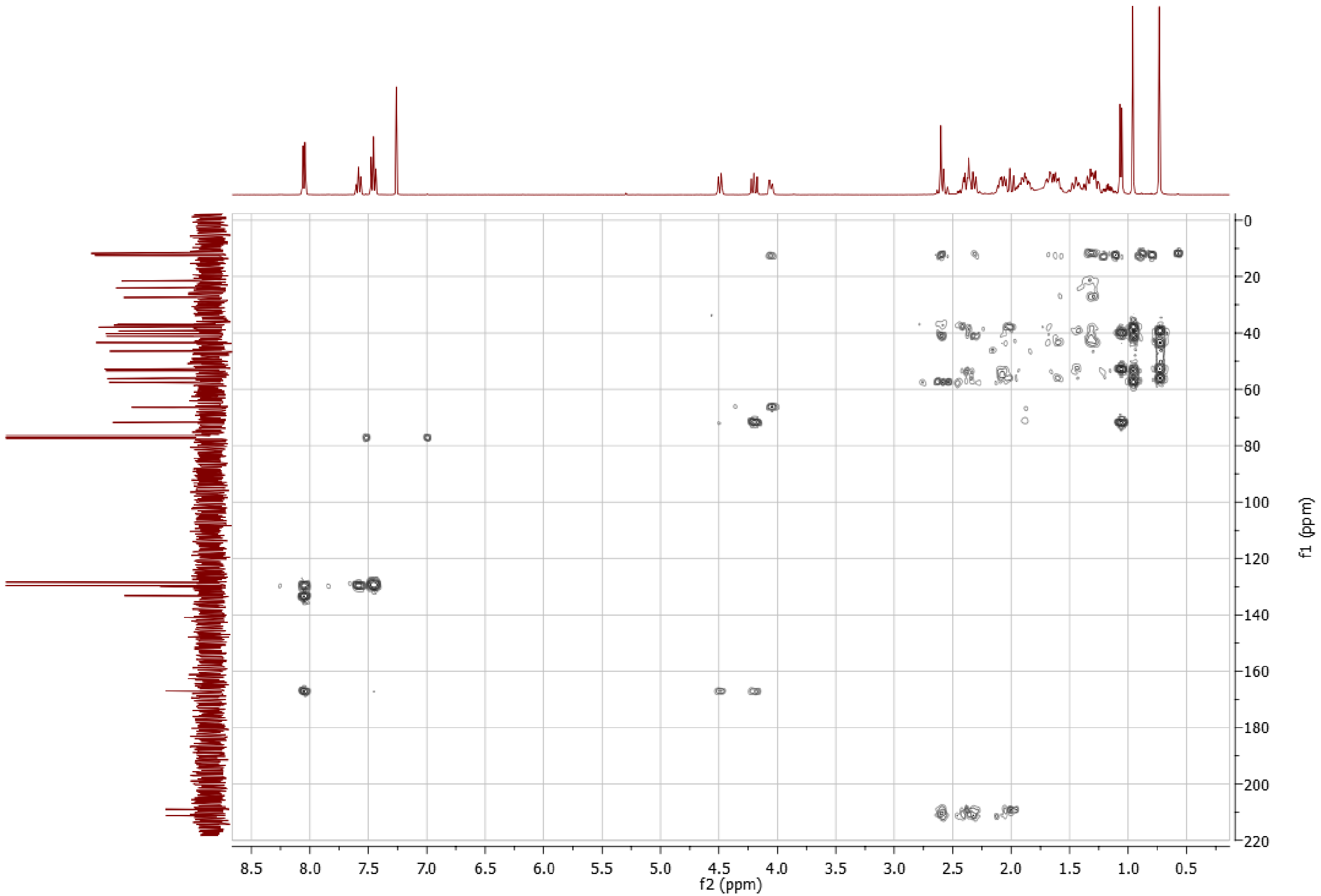

S31. 2D HMBC NMR spectrum of *22(S)-hydroxy-24-nor-5α-cholan-3,6-dioxobenzoate-23-yl* (***41a***)

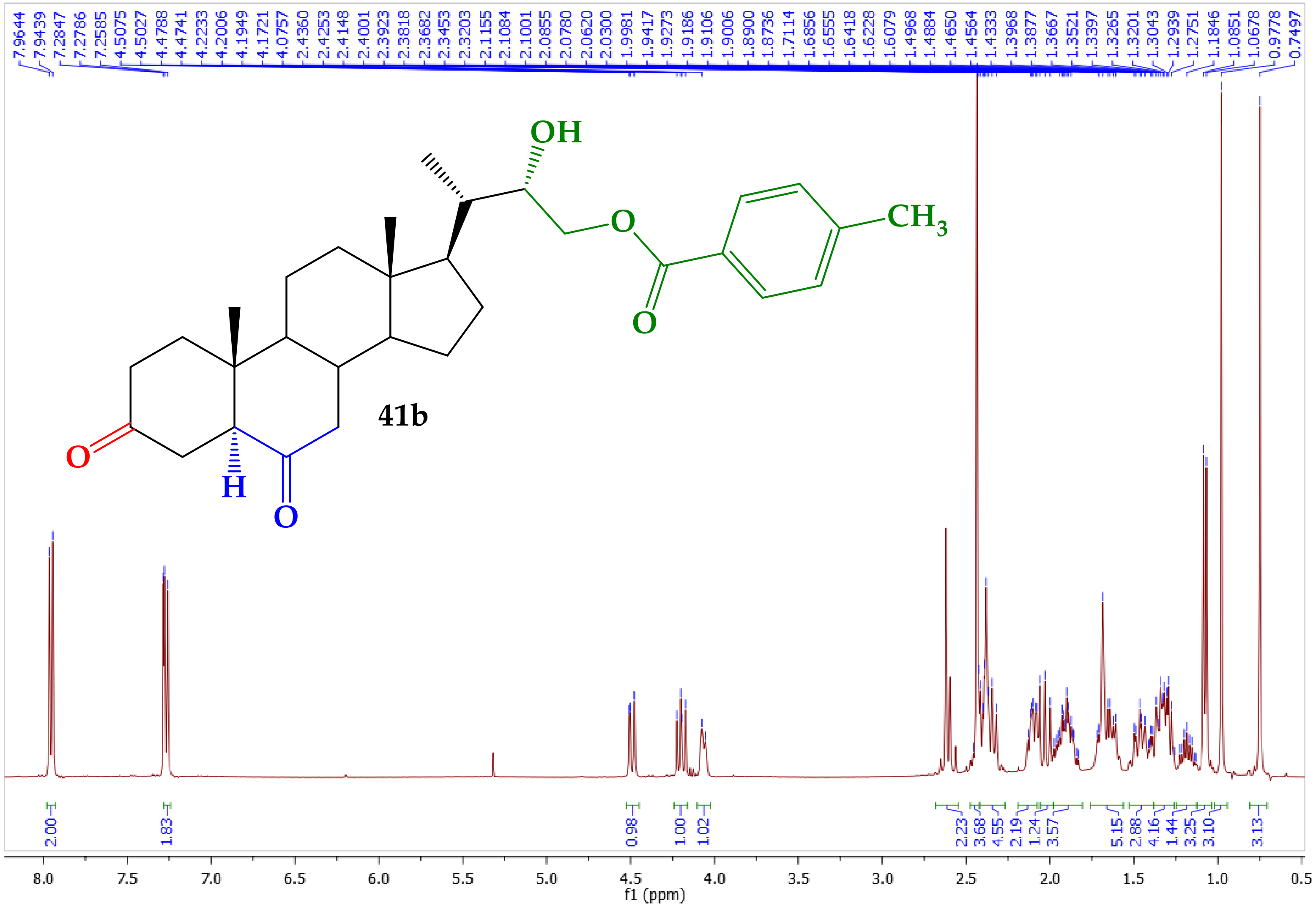

S32. ^{1}H NMR spectrum of *22(S)-hydroxy-24-nor-5α-cholan-3,6-dioxo-(4-methyl)-benzoate-23-yl* (***41b***)

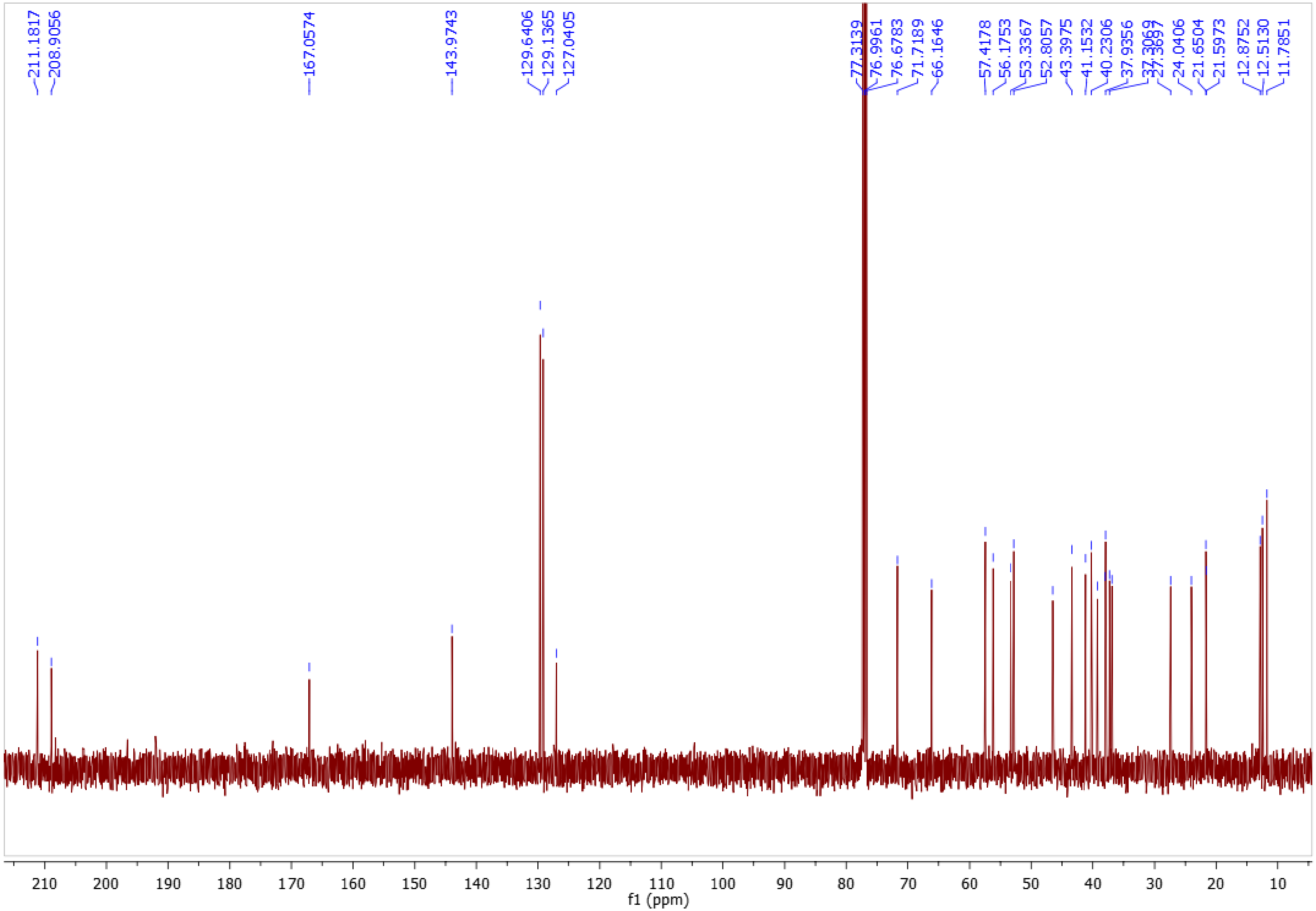

S33. ^{13}C NMR spectrum of *22(S)-hydroxy-24-nor-5α-cholan-3,6-dioxo-(4-methyl)-benzoate-23-yl* (***41b***)

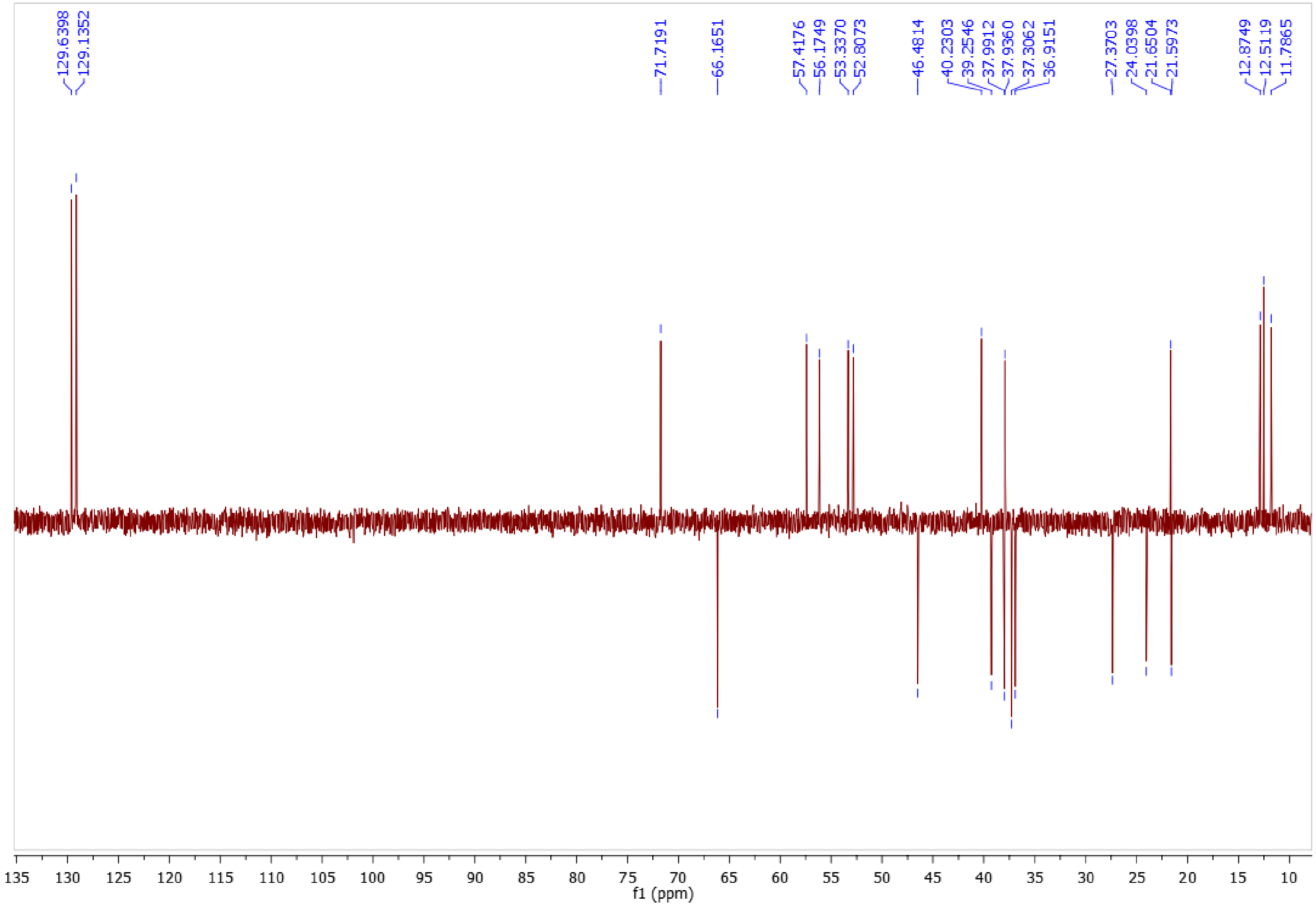

S34. ^{13}C DEPT-135 NMR spectrum of *22(S)-hydroxy-24-nor-5α-cholan-3,6-dioxo-(4-methyl)-benzoate-23-yl* (***41b***)

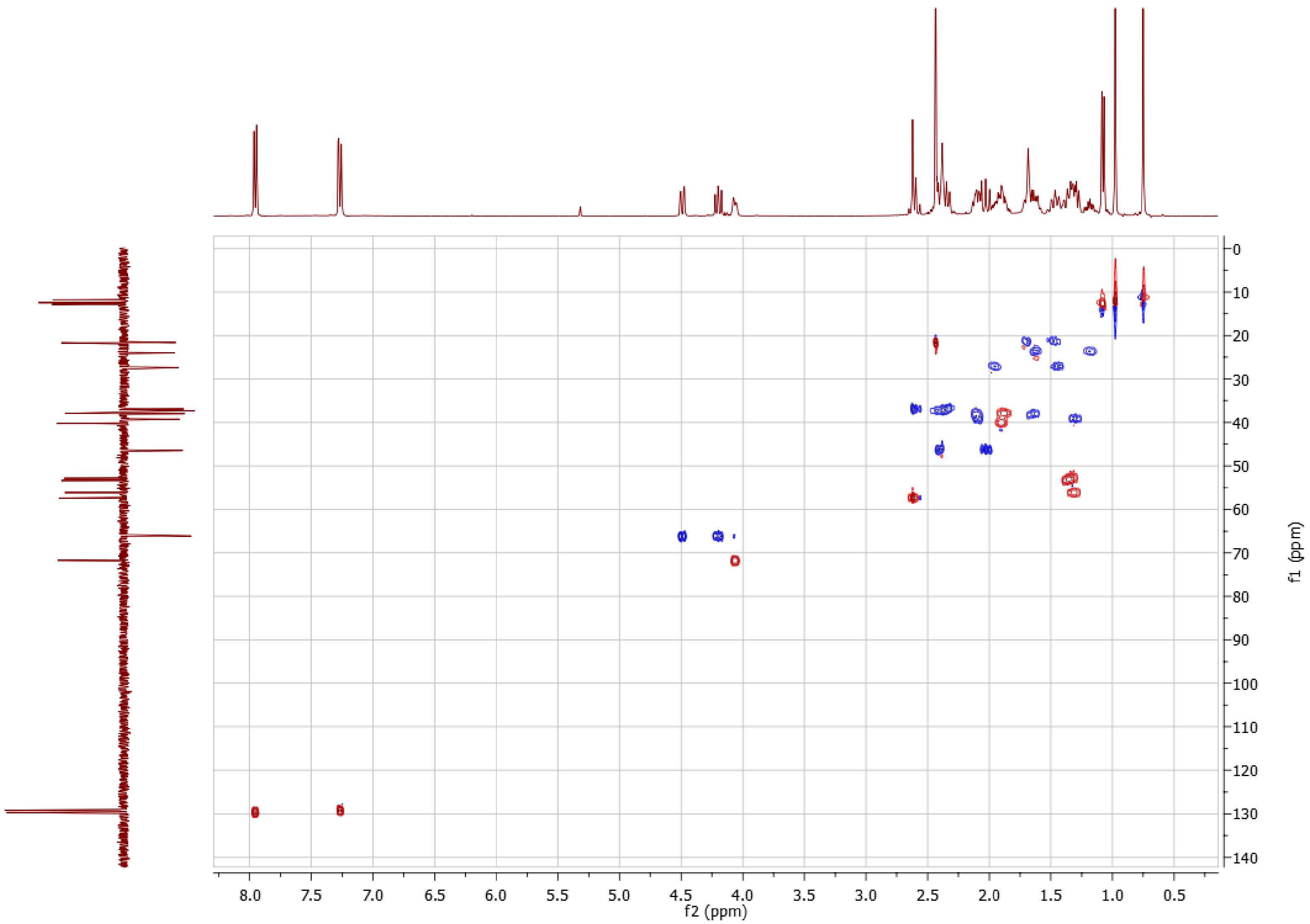

S35. 2D HSQC NMR spectrum of *22(S)-hydroxy-24-nor-5α-cholan-3,6-dioxo-(4-methyl)-benzoate-23-yl* (***41b***)

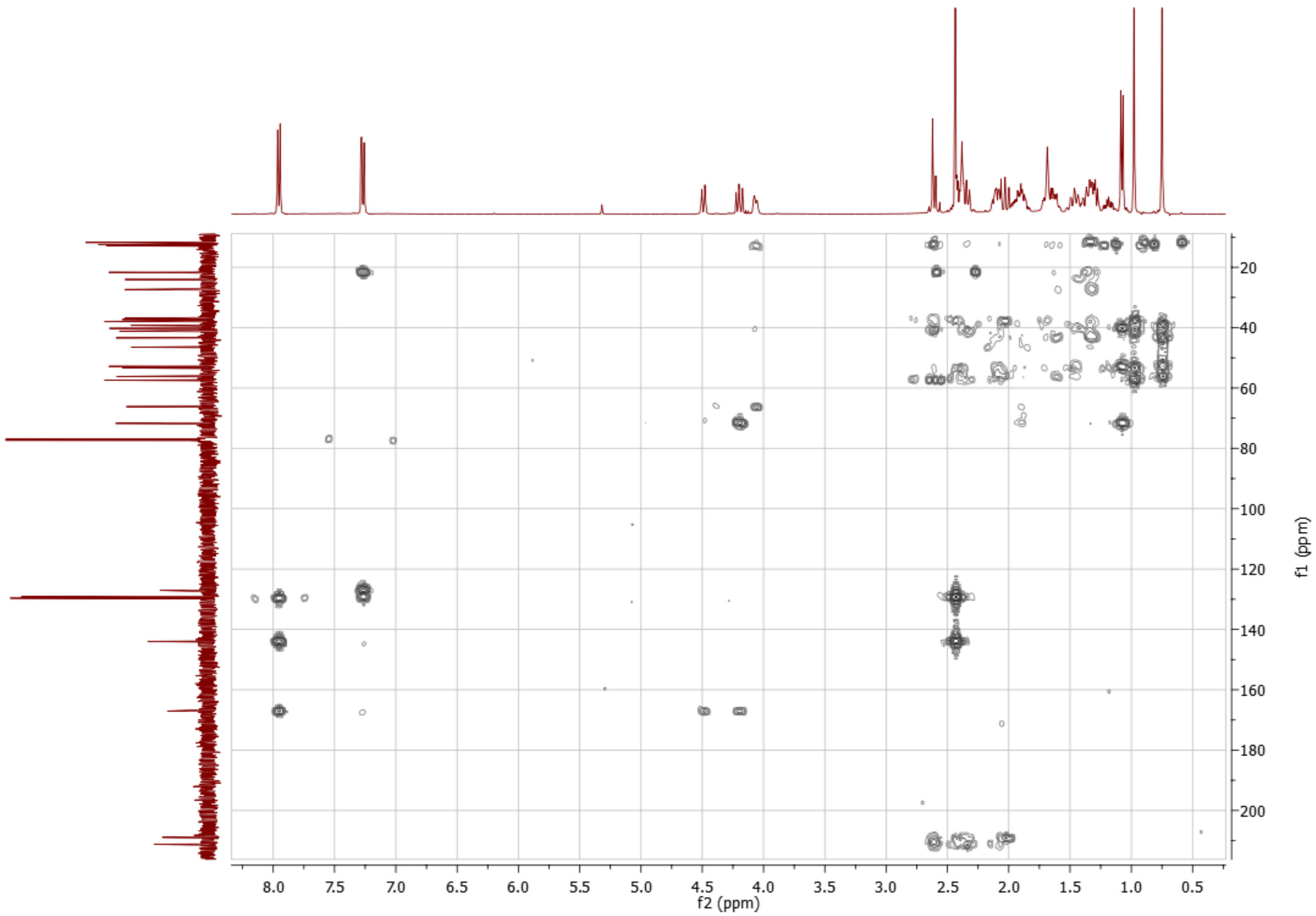

S36. 2D HMBC NMR spectrum of *22(S)-hydroxy-24-nor-5α-cholan-3,6-dioxo-(4-methyl)-benzoate-23-yl* (***41b***)

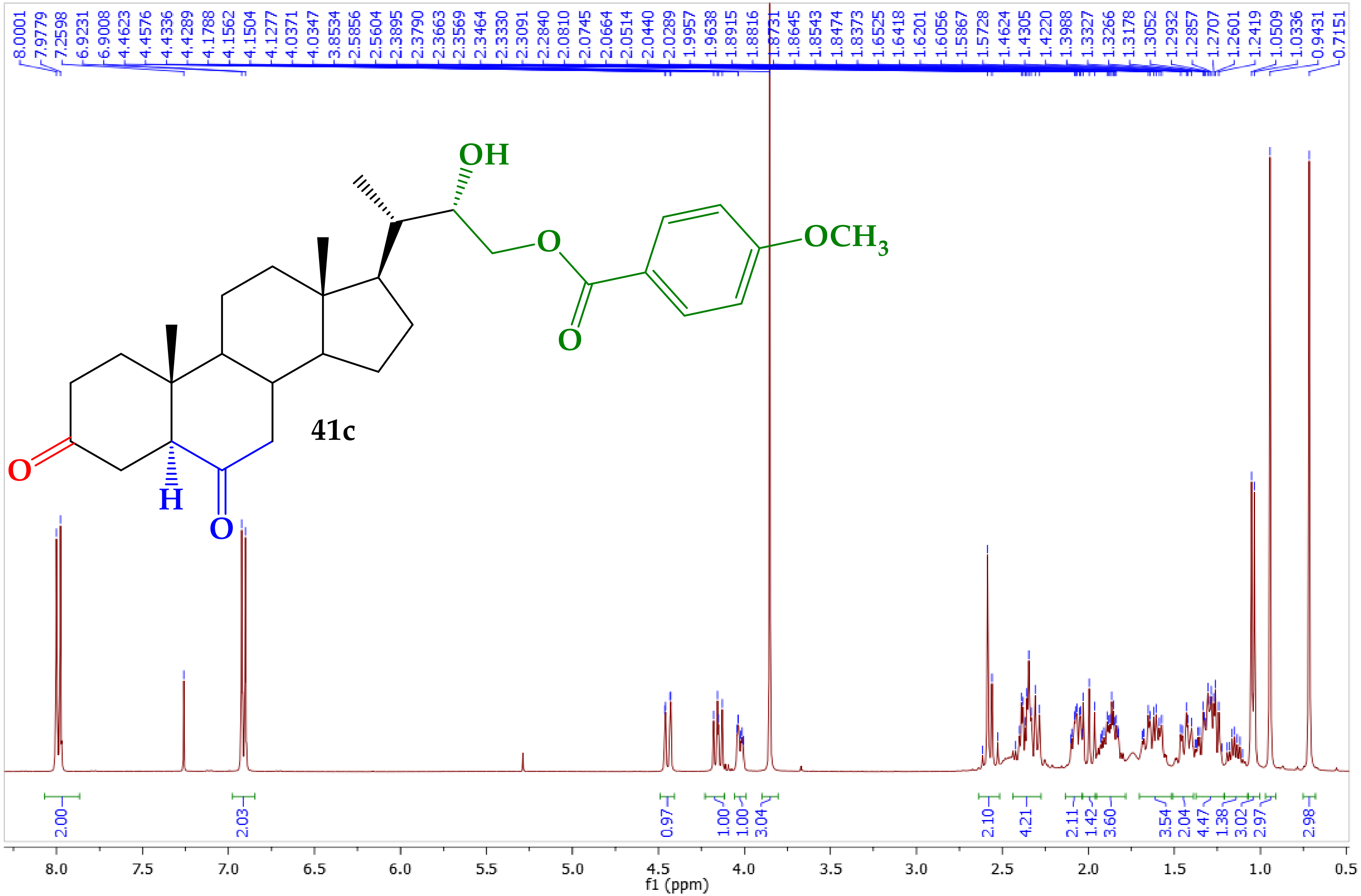

S37. ^{1}H NMR spectrum of *22(S)-hydroxy-24-nor-5α-cholan-3,6-dioxo-(4-metoxy)-benzoate-23-yl* (***41c***)

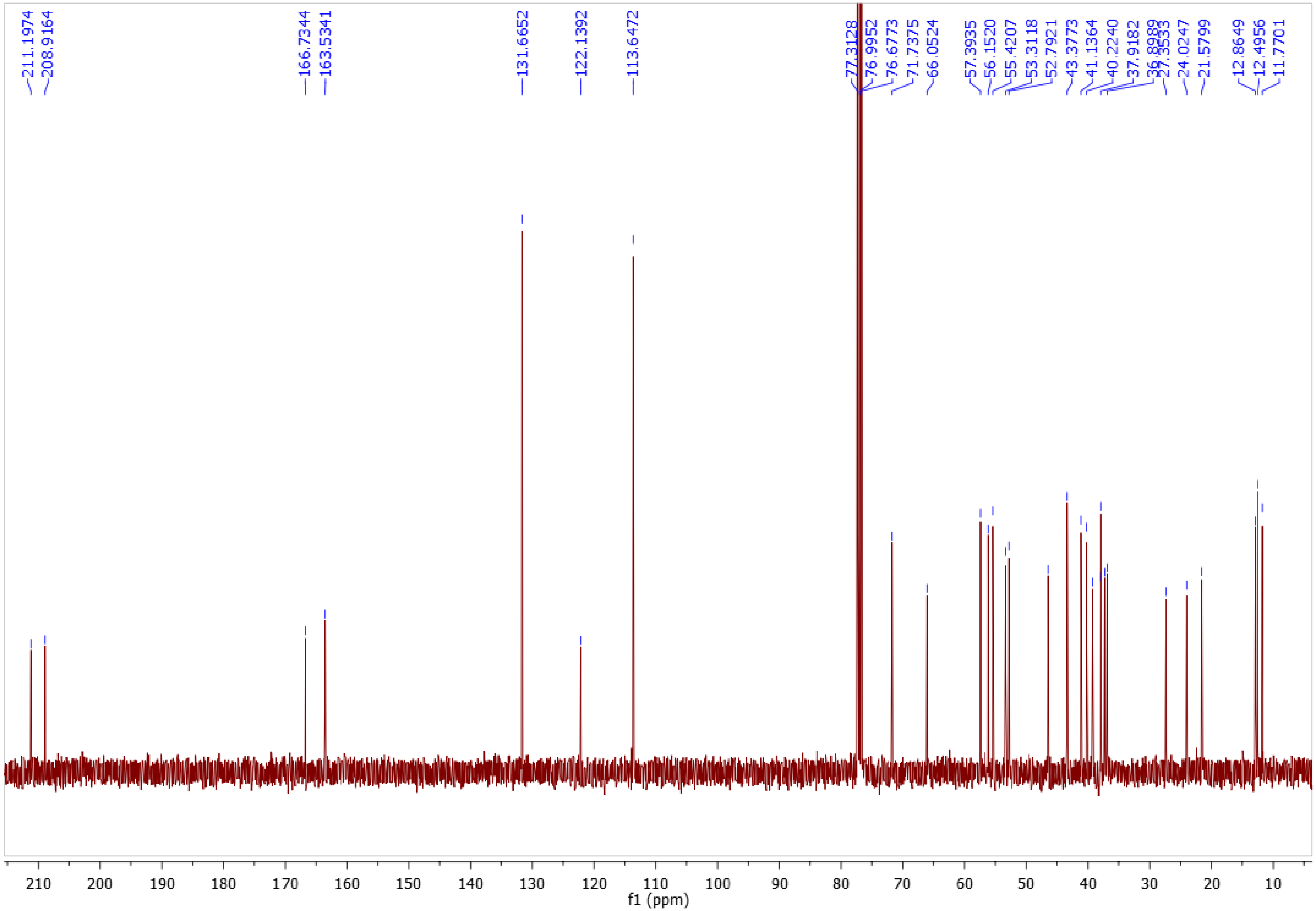

S38. ^{13}C NMR spectrum of *22(S)-hydroxy-24-nor-5α-cholan-3,6-dioxo-(4-metoxy)-benzoate-23-yl* (***41c***)

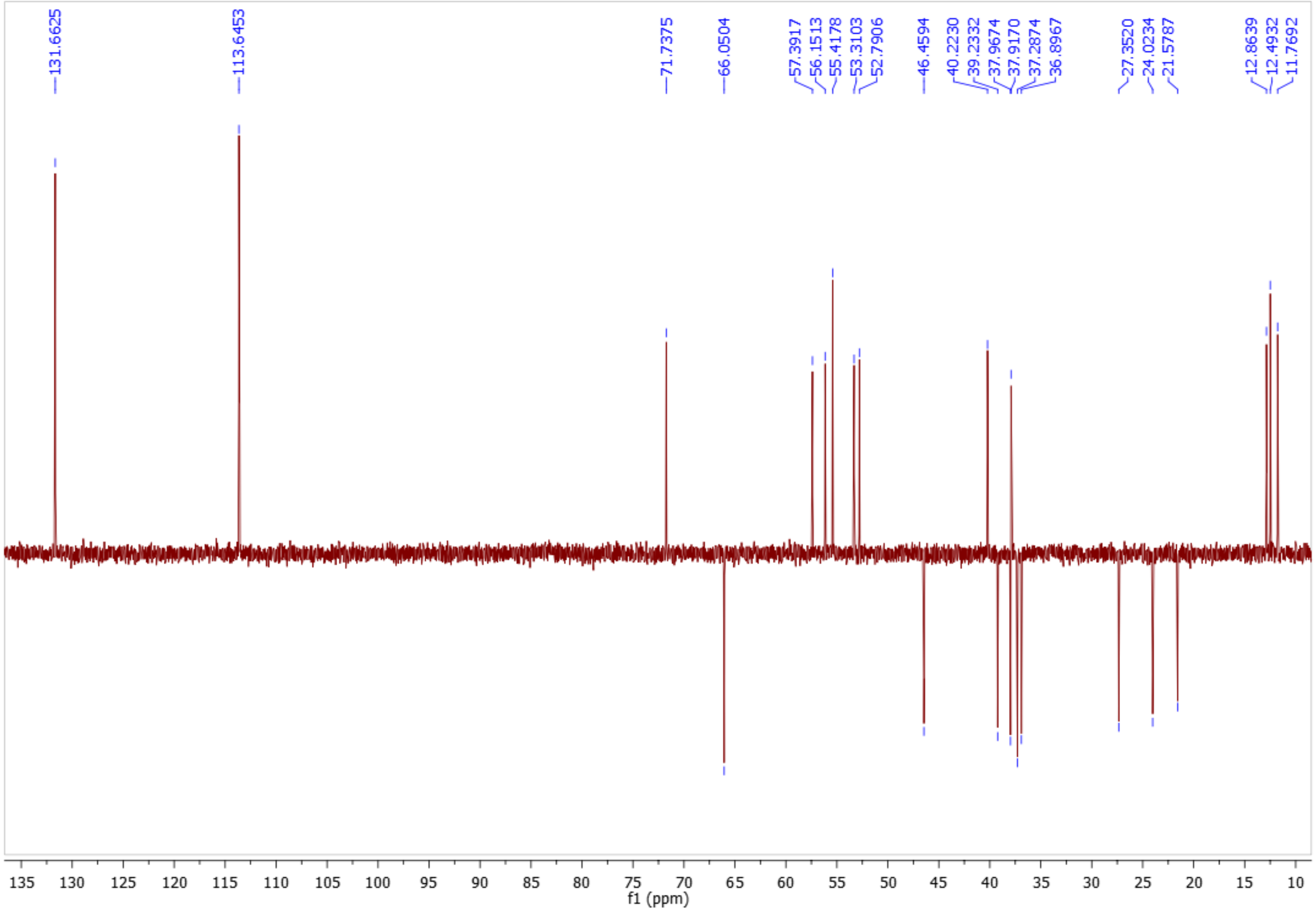

S39. ^{13}C DEPT-135 NMR spectrum of *22(S)-hydroxy-24-nor-5α-cholan-3,6-dioxo-(4-metoxy)-benzoate-23-yl* (***41c***)

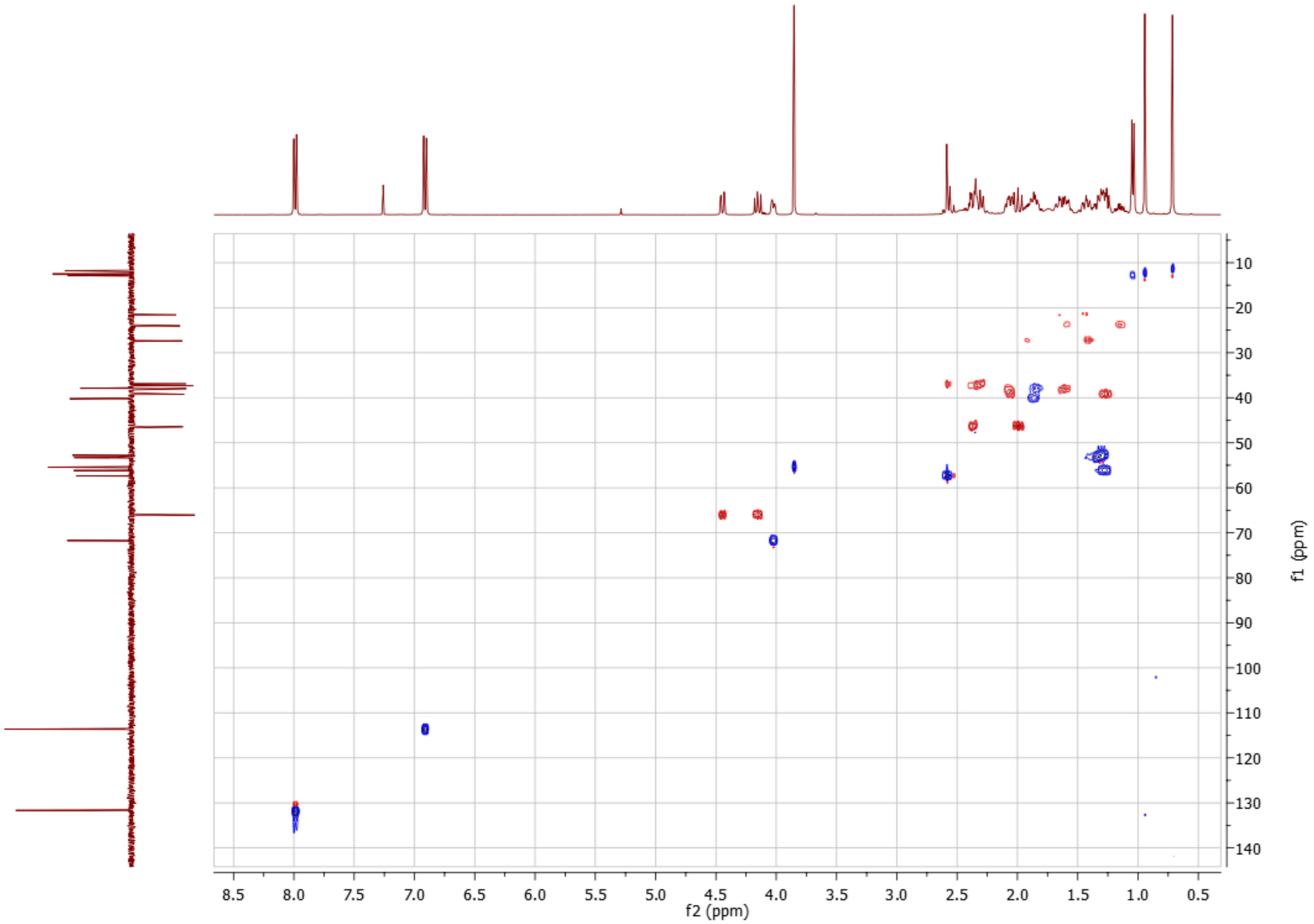

S40. 2D HSQC NMR spectrum of *22(S)-hydroxy-24-nor-5α-cholan-3,6-dioxo-(4-metoxy)-benzoate-23-yl* (***41c***)

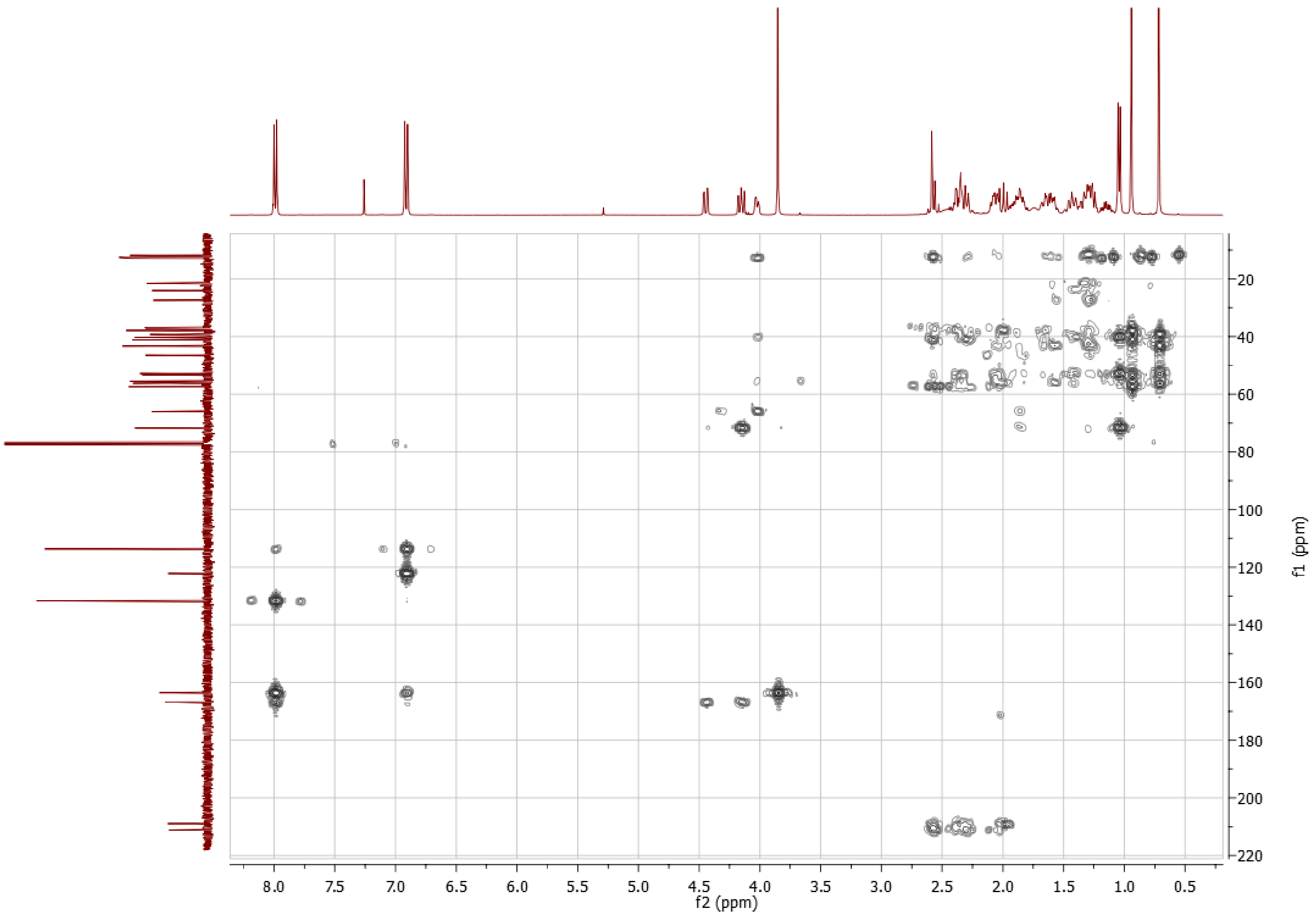

S41. 2D HMBC NMR spectrum of *22(S)-hydroxy-24-nor-5α-cholan-3,6-dioxo-(4-metoxy)-benzoate-23-yl* (***41c***)

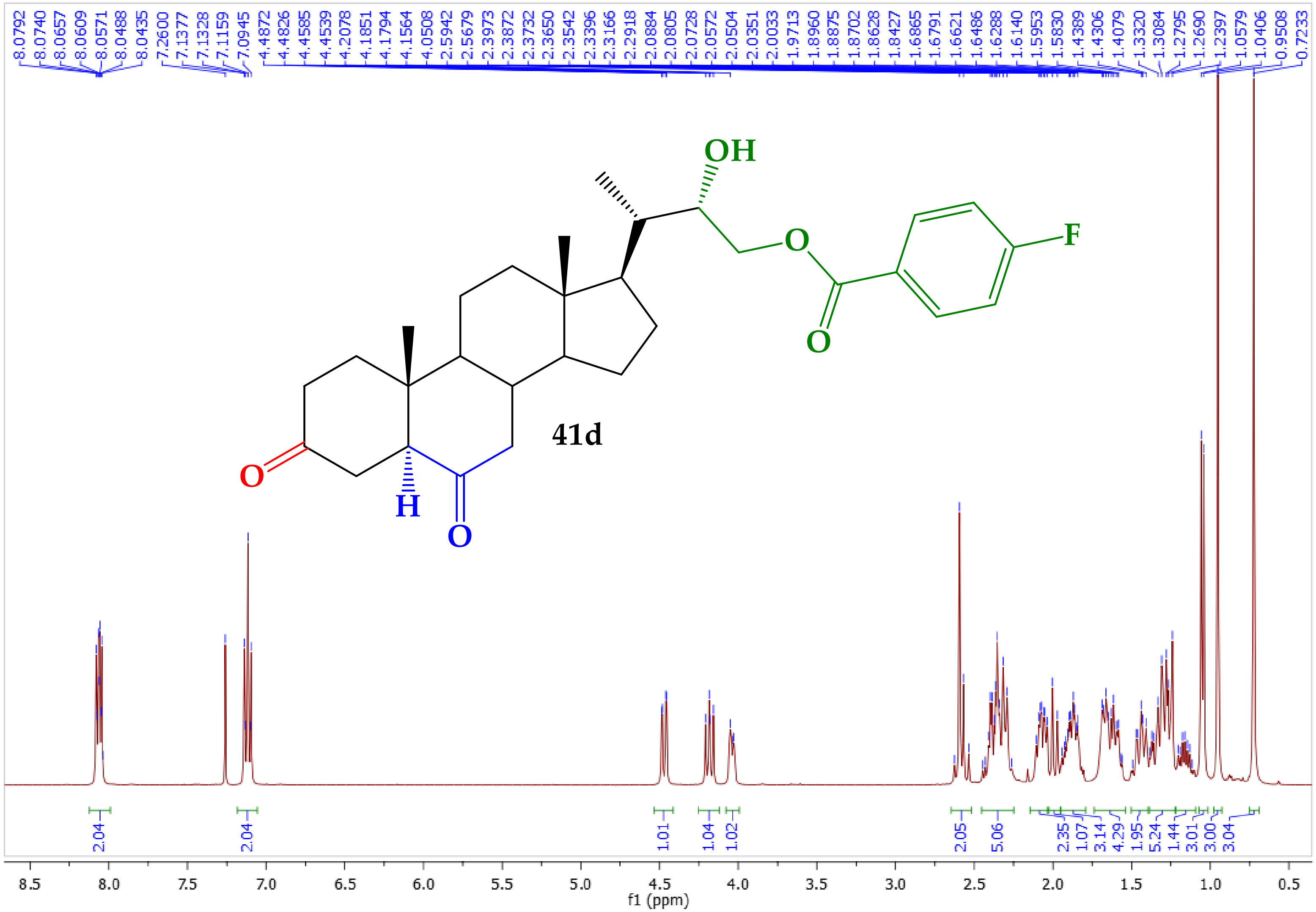

S42. ^{1}H NMR spectrum of *22(S)-hydroxy-24-nor-5α-cholan-3,6-dioxo-(4-fluoro)-benzoate-23-yl* (***41d***)

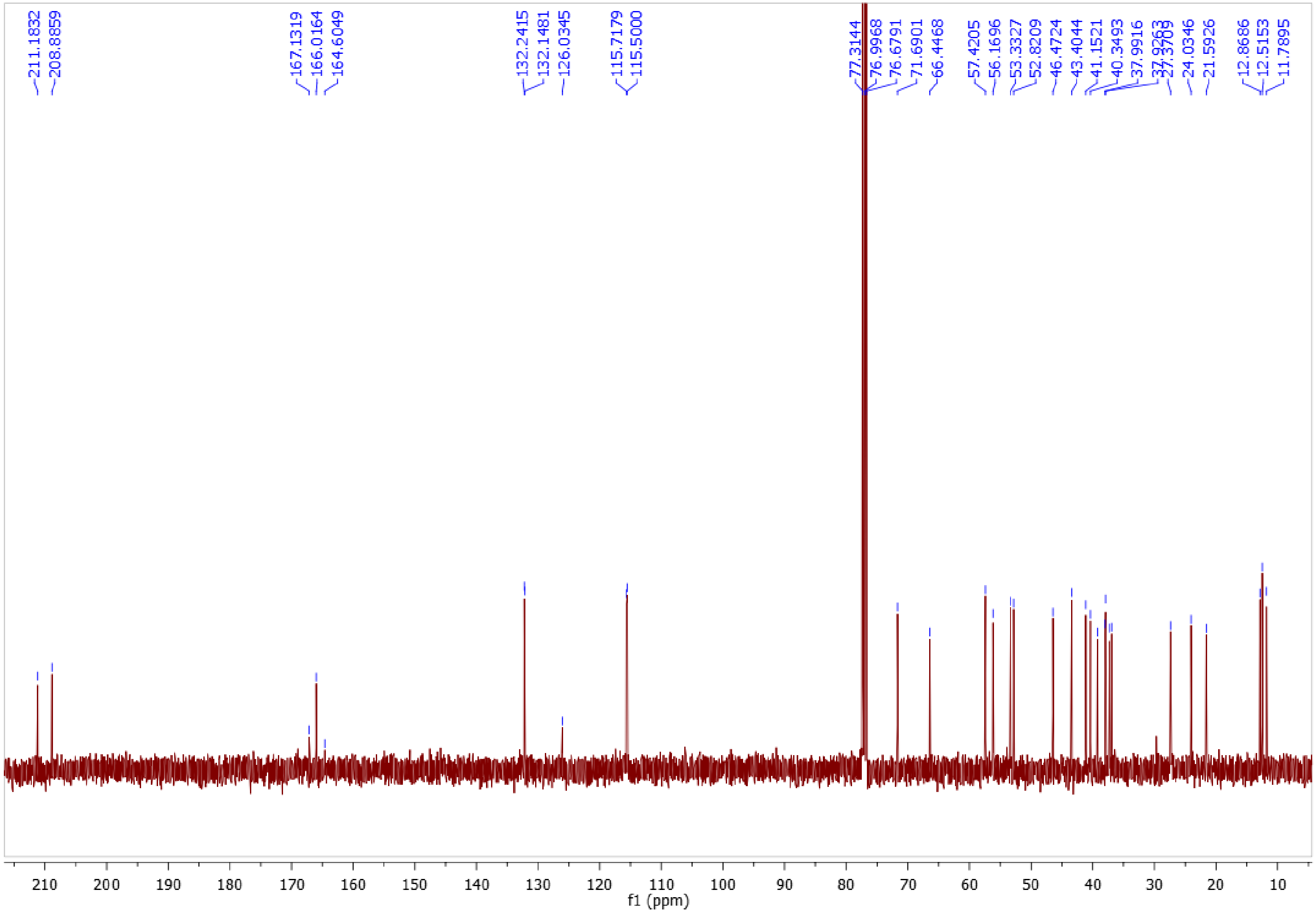

S43. ^{13}C NMR spectrum of *22(S)-hydroxy-24-nor-5α-cholan-3,6-dioxo-(4-fluoro)-benzoate-23-yl* (***41d***)

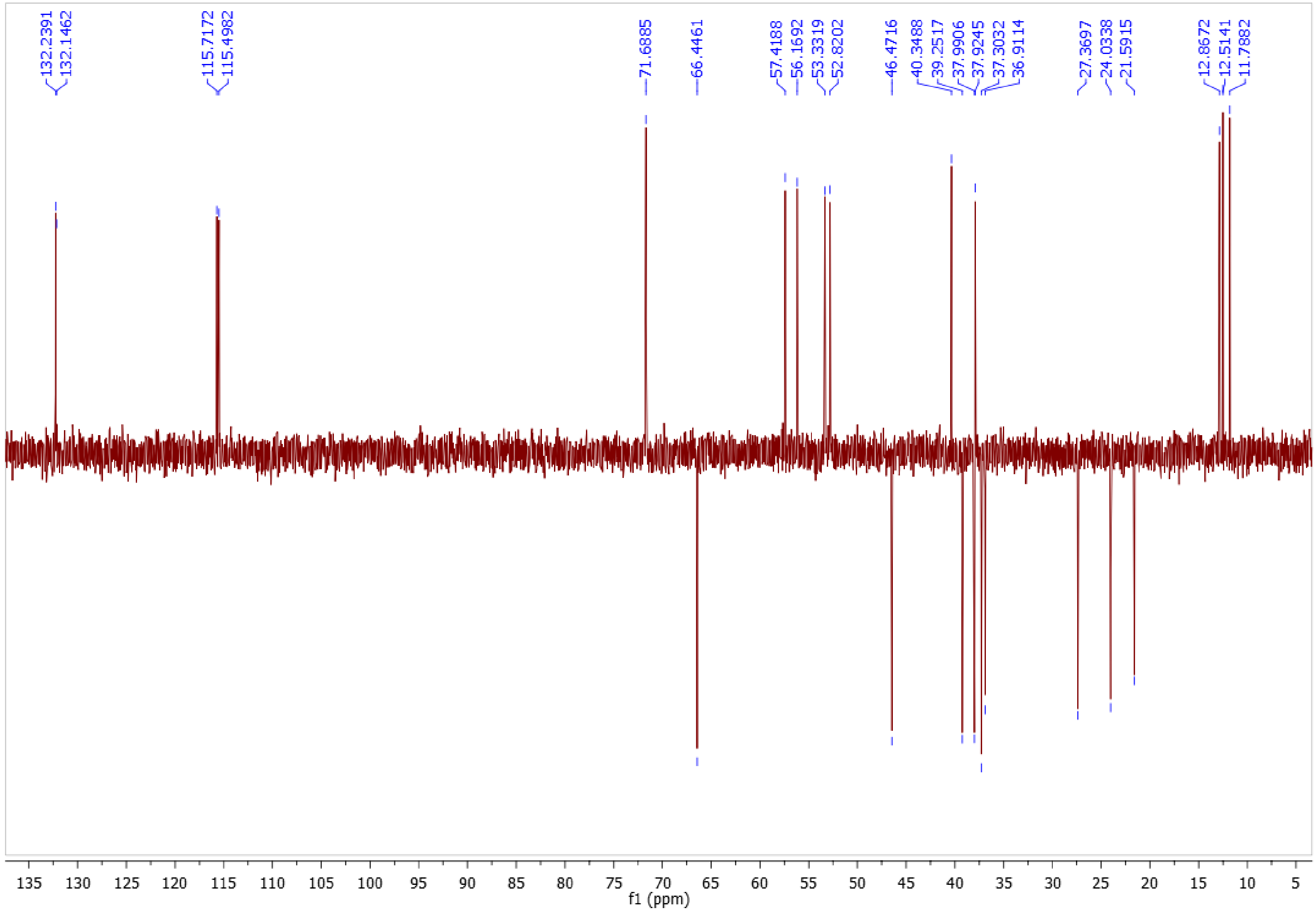

S44. ^{13}C DEPT-135 NMR spectrum of *22(S)-hydroxy-24-nor-5α-cholan-3,6-dioxo-(4-fluoro)-benzoate-23-yl* (***41d***)

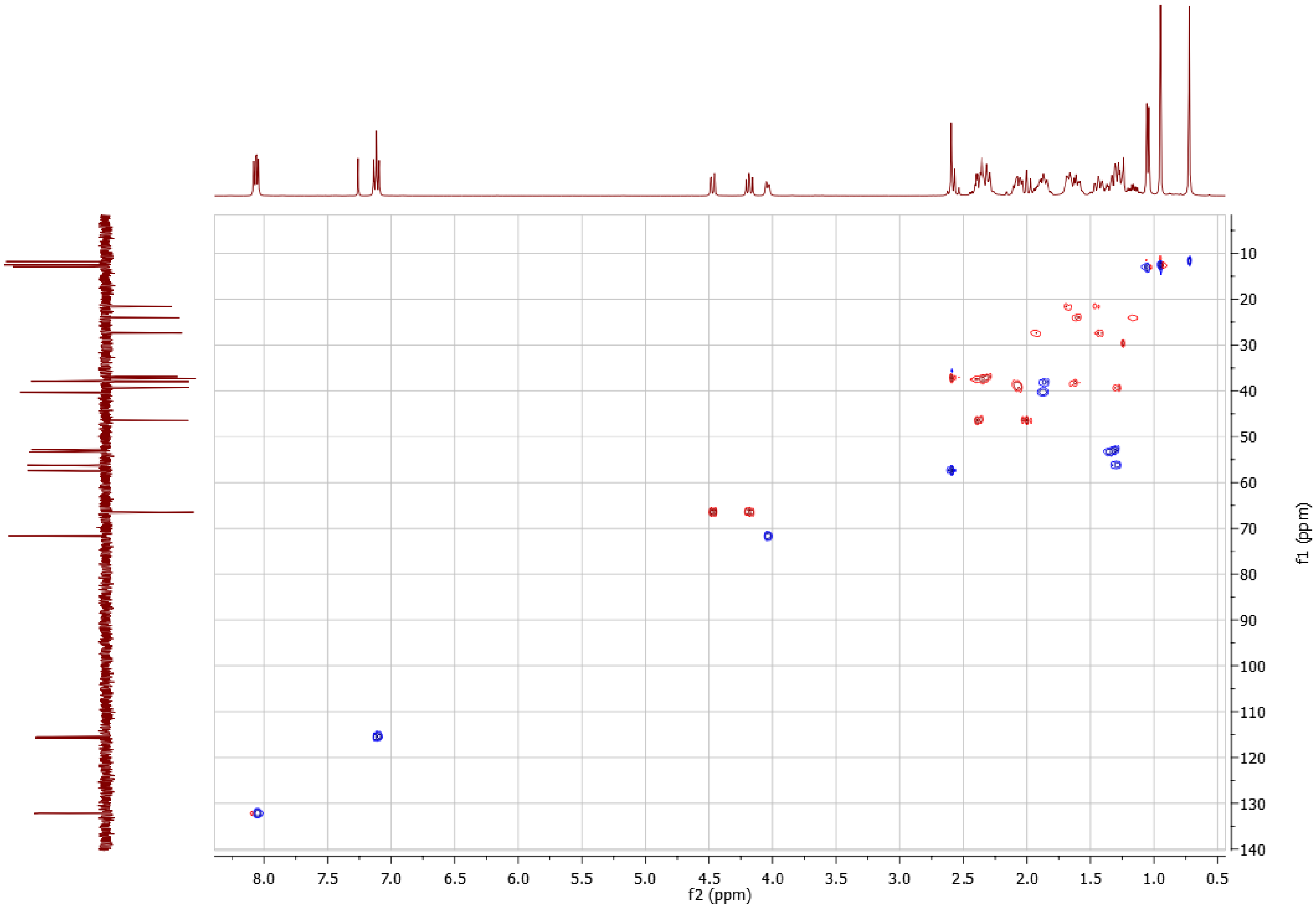

S45. 2D HSQC NMR spectrum of *22(S)-hydroxy-24-nor-5α-cholan-3,6-dioxo-(4-fluoro)-benzoate-23-yl* (***41d***)

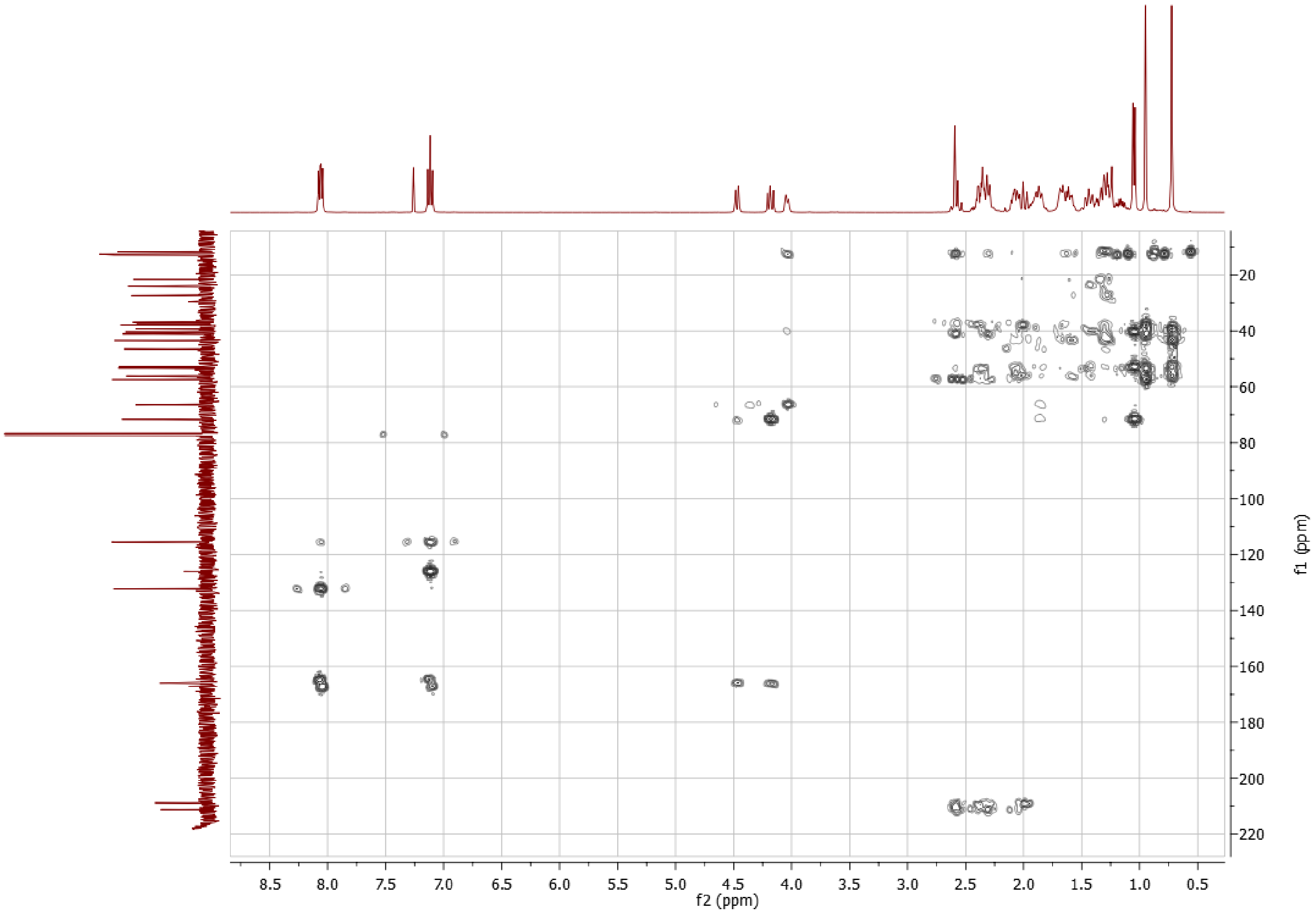

S46. 2D HMBC NMR spectrum of *22(S)-hydroxy-24-nor-5α-cholan-3,6-dioxo-(4-fluoro)-benzoate-23-yl* (***41d***)

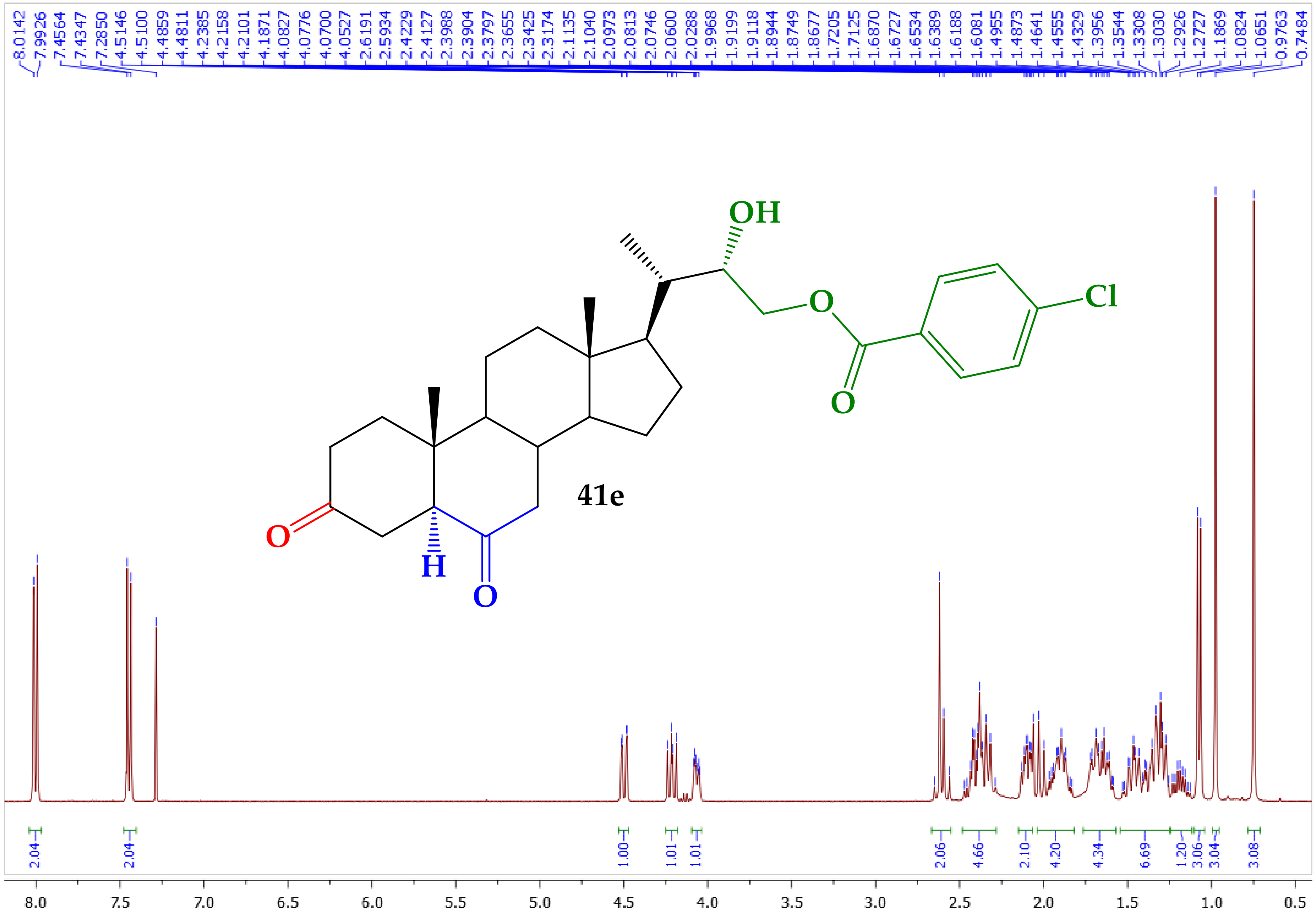

S47. ^{1}H NMR spectrum of *22(S)-hydroxy-24-nor-5α-cholan-3,6-dioxo-(4-chloro)-benzoate-23-yl* (***41e***)

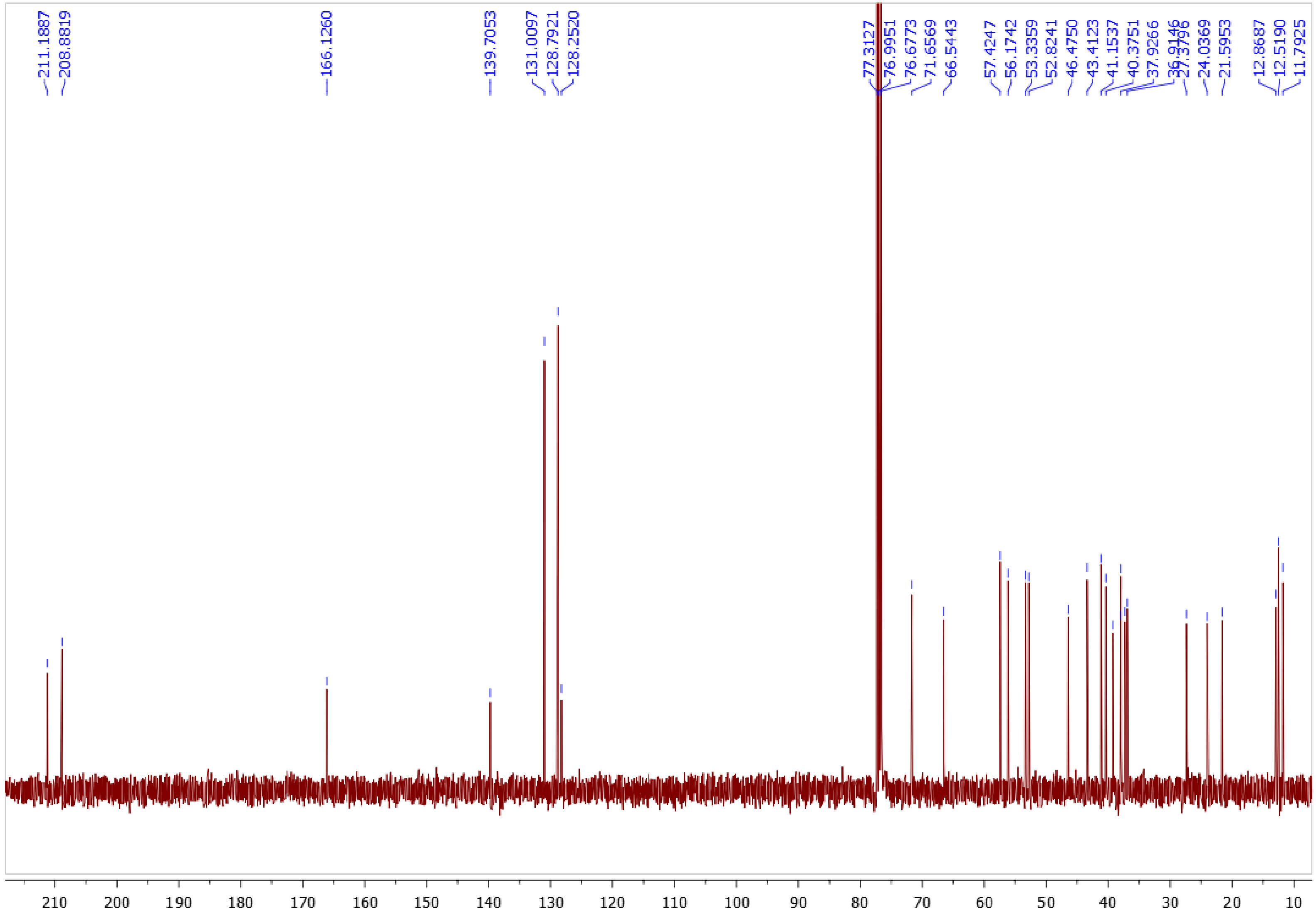

S48. ^{13}C NMR spectrum of *22(S)-hydroxy-24-nor-5α-cholan-3,6-dioxo-(4-chloro)-benzoate-23-yl* (***41e***)

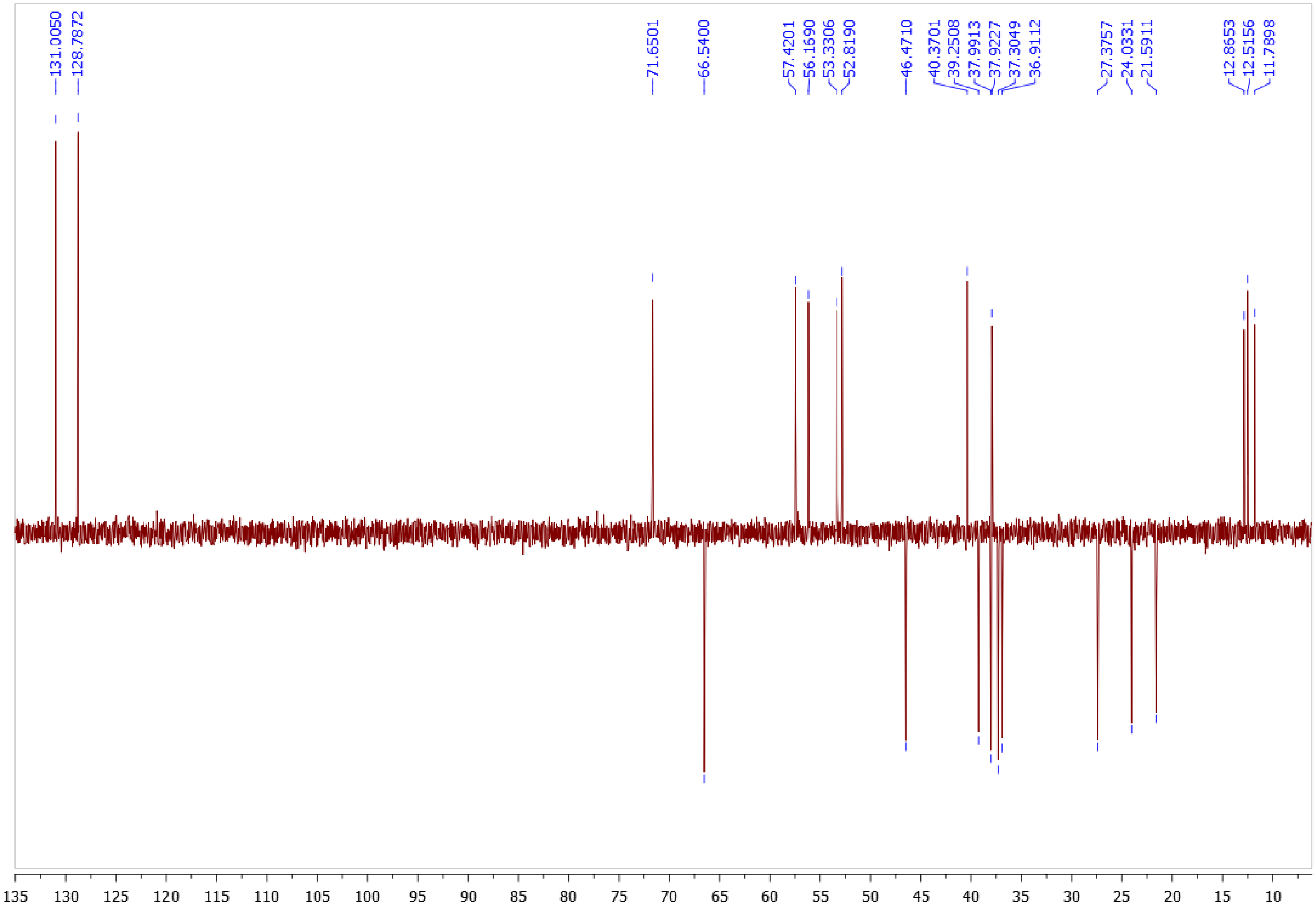

S49. ^{13}C DEPT-135 NMR spectrum of *22(S)-hydroxy-24-nor-5α-cholan-3,6-dioxo-(4-chloro)-benzoate-23-yl* (***41e***)

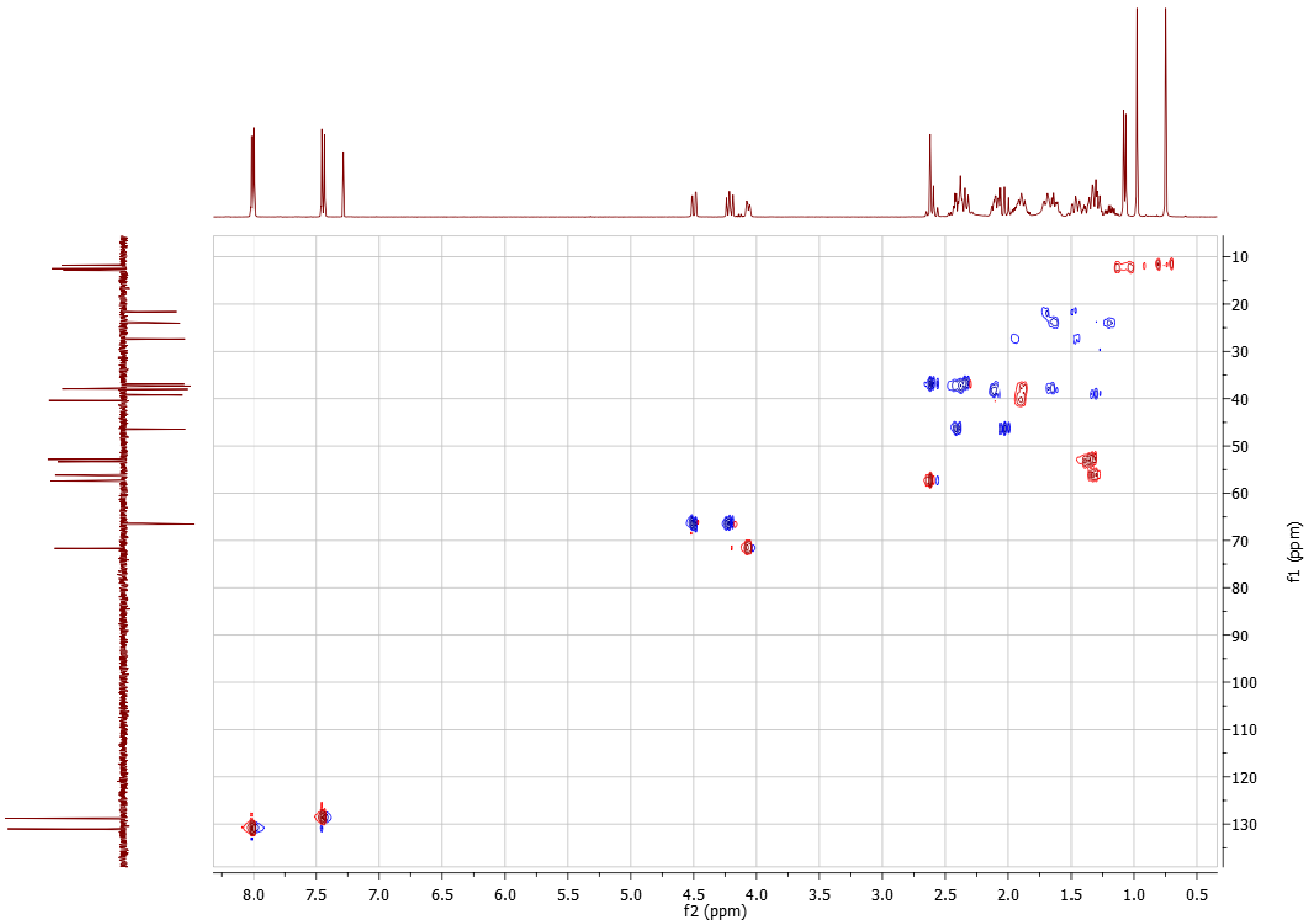

S50. 2D HSQC NMR spectrum of *22(S)-hydroxy-24-nor-5α-cholan-3,6-dioxo-(4-chloro)-benzoate-23-yl* (***41e***)

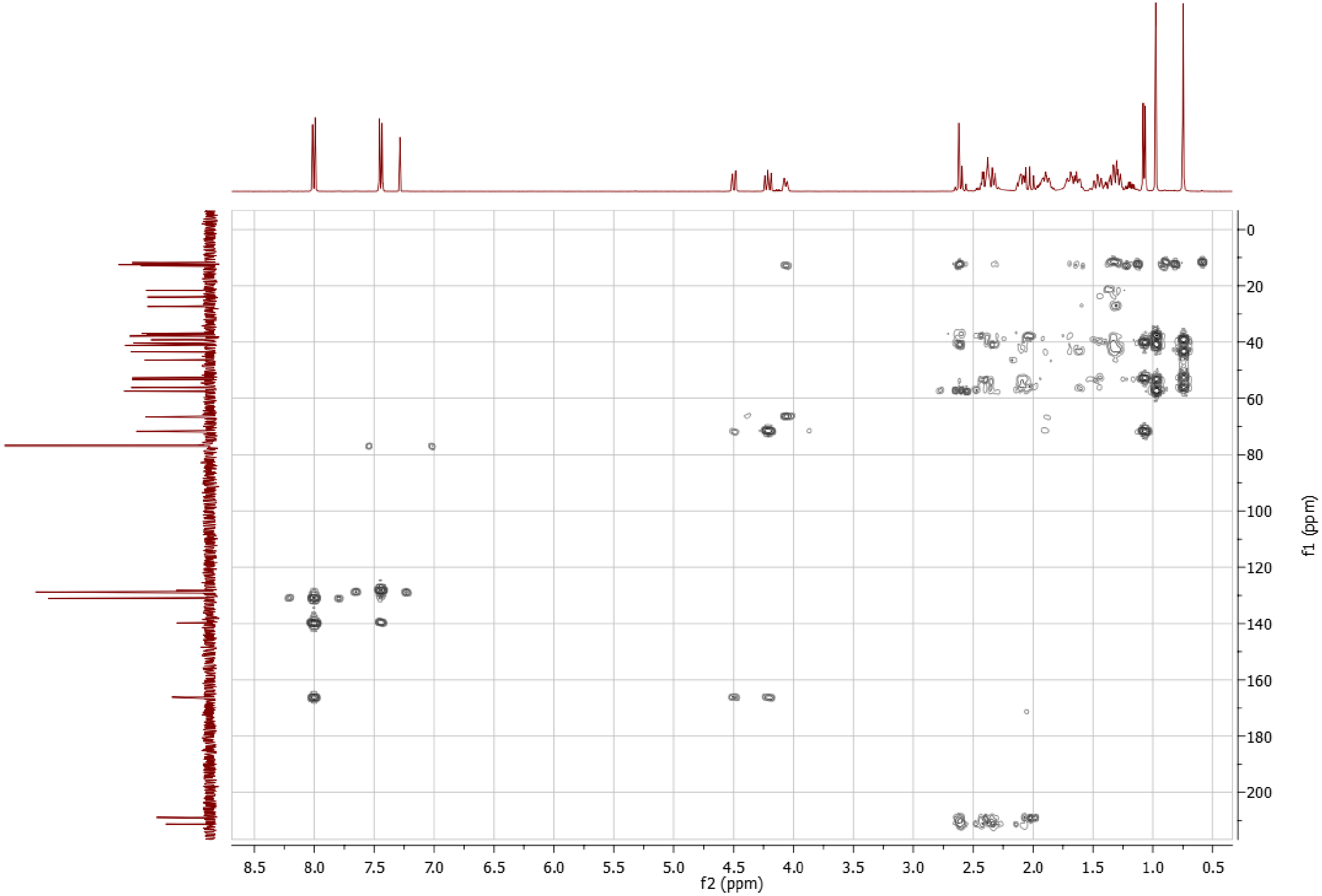

S51. 2D HMBC NMR spectrum of *22(S)-hydroxy-24-nor-5α-cholan-3,6-dioxo-(4-chloro)-benzoate-23-yl* (***41e***)

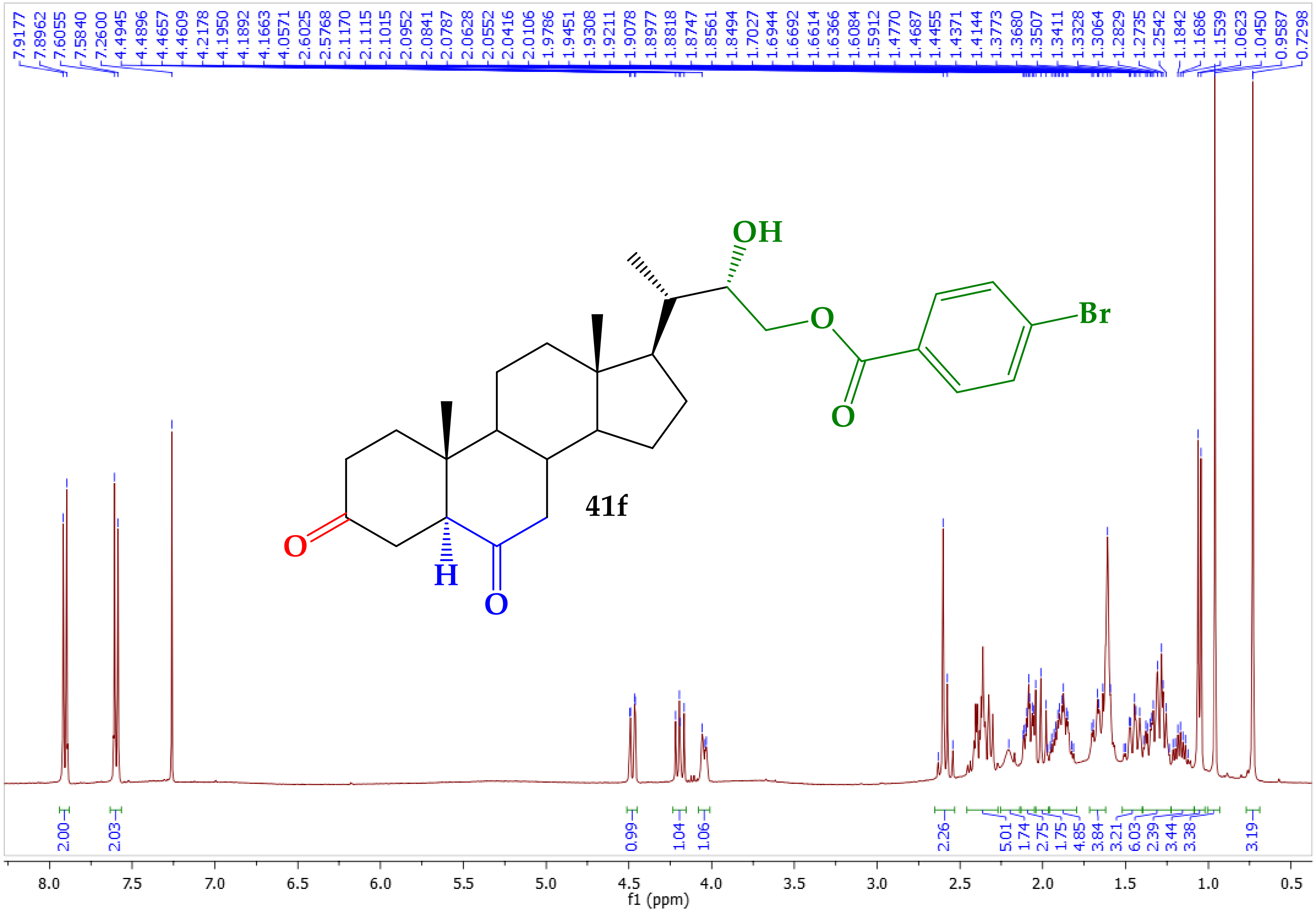

S52. ^{1}H NMR spectrum of *22(S)-hydroxy-24-nor-5α-cholan-3,6-dioxo-(4-bromo)-benzoate-23-yl* (***41f***)

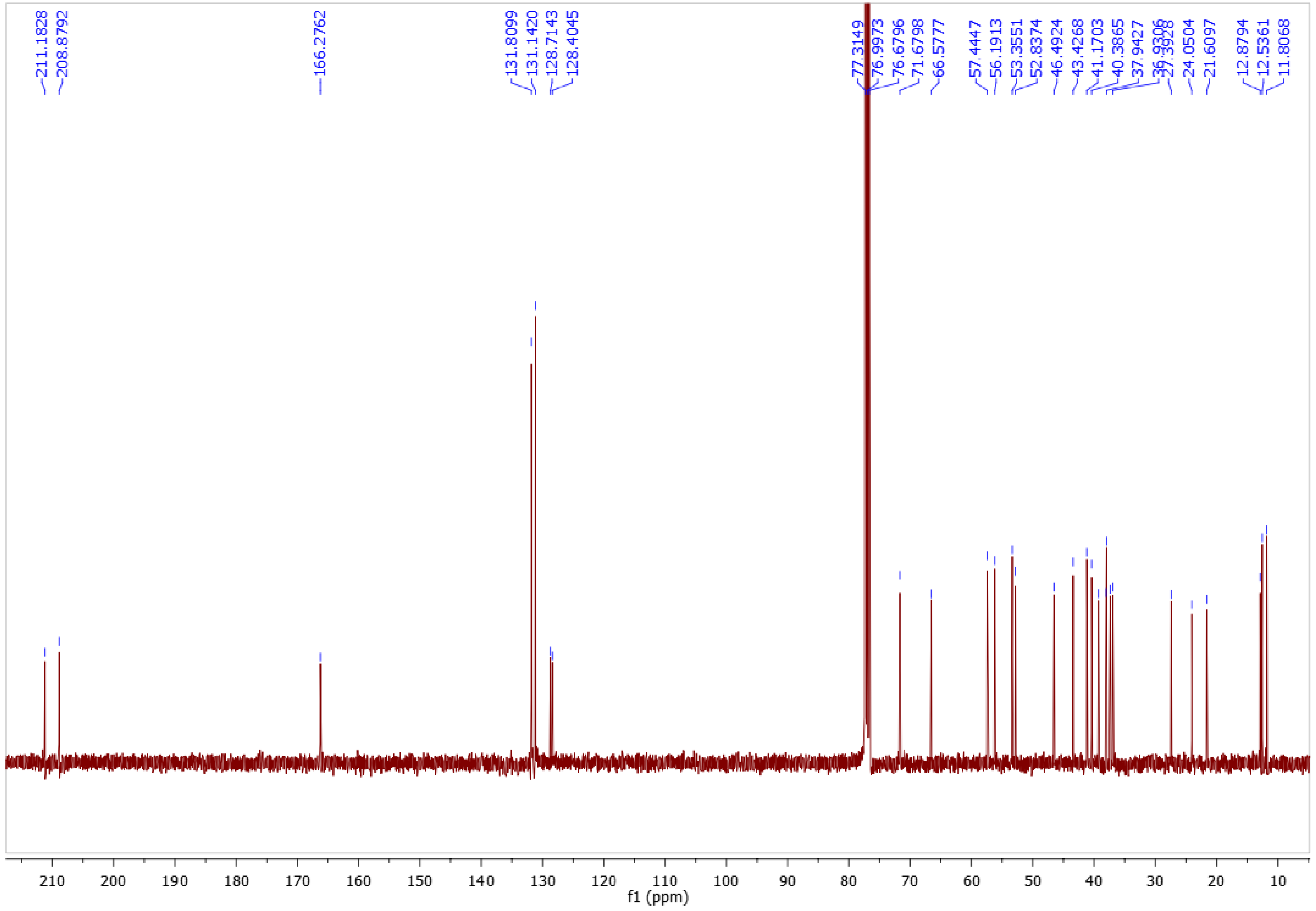

S53. ^{13}C NMR spectrum of *22(S)-hydroxy-24-nor-5α-cholan-3,6-dioxo-(4-bromo)-benzoate-23-yl* (***41f***)

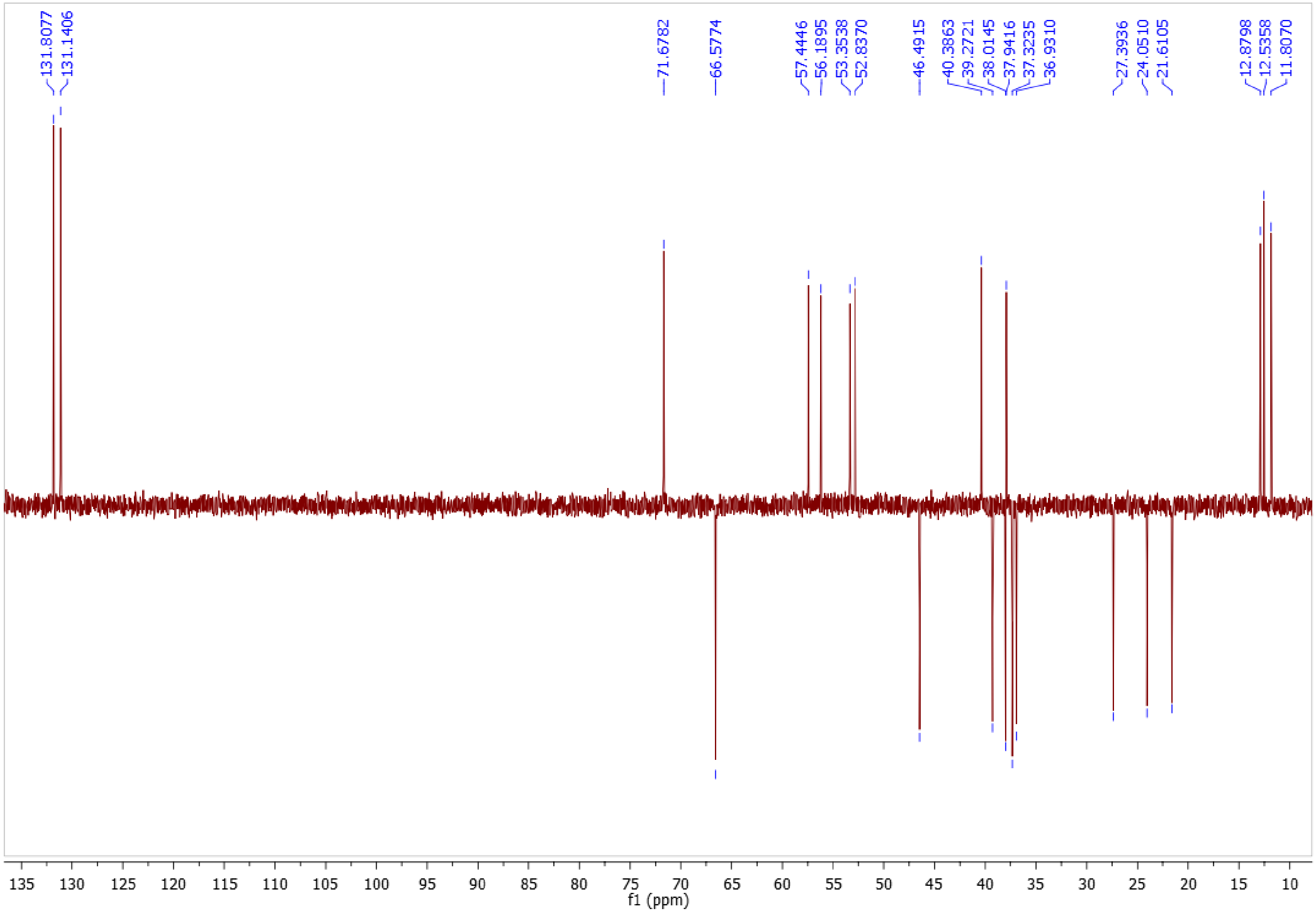

S54. ^{13}C DEPT-135 NMR spectrum of *22(S)-hydroxy-24-nor-5α-cholan-3,6-dioxo-(4-bromo)-benzoate-23-yl* (***41f***)

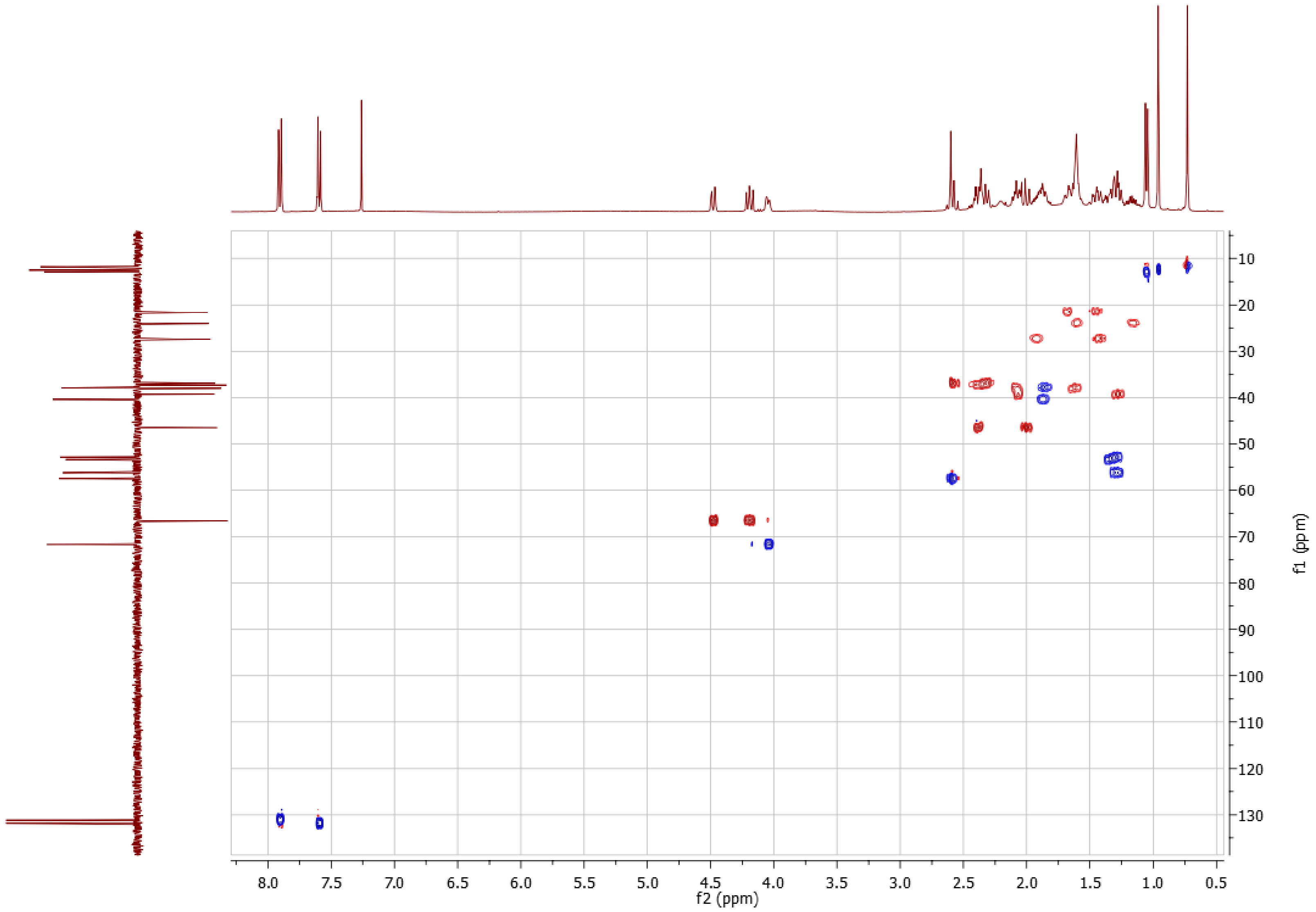

S55. 2D HSQC NMR spectrum of *22(S)-hydroxy-24-nor-5α-cholan-3,6-dioxo-(4-bromo)-benzoate-23-yl* (***41f***)

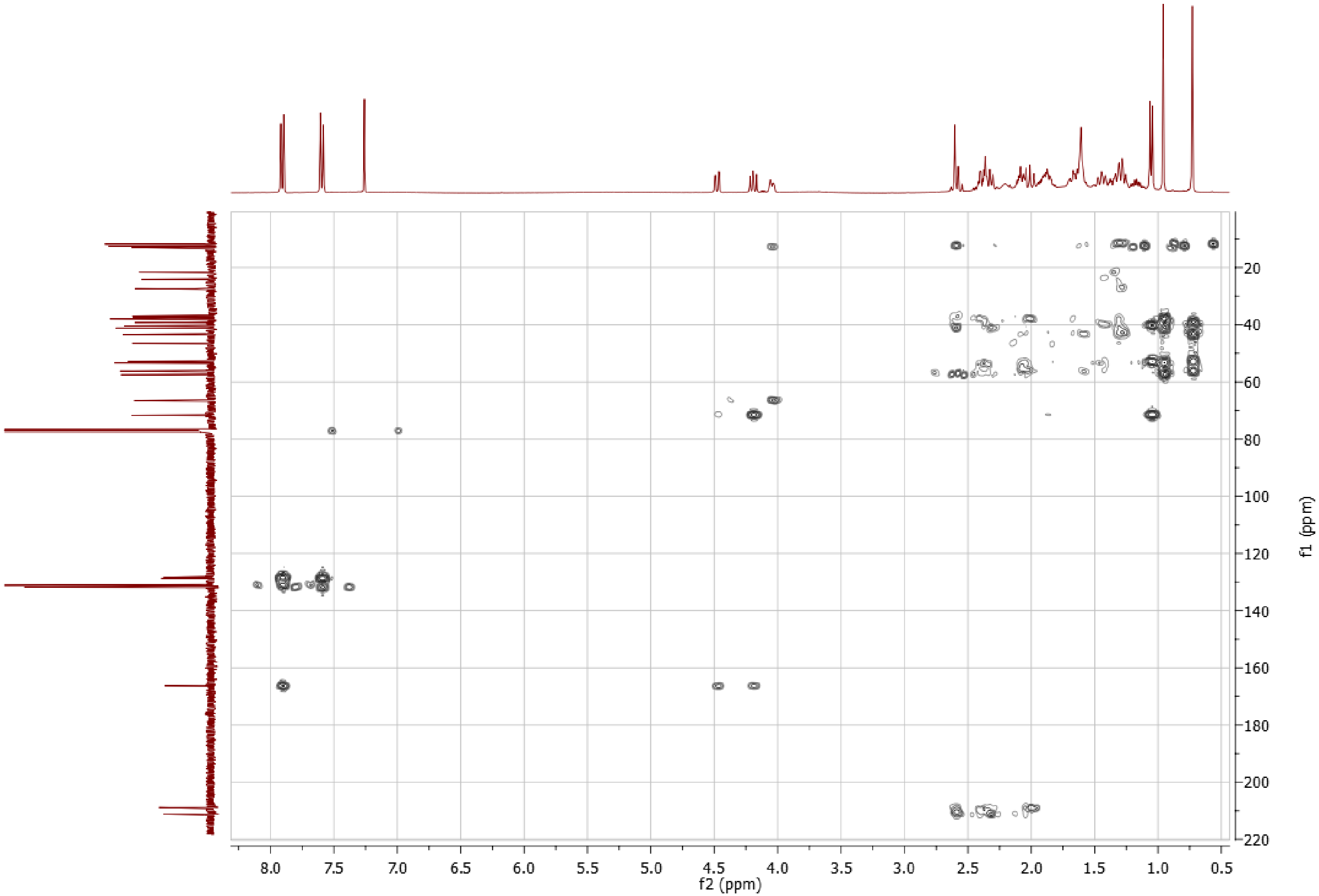

S56. 2D HMBC NMR spectrum of *22(S)-hydroxy-24-nor-5α-cholan-3,6-dioxo-(4-bromo)-benzoate-23-yl* (***41f***)

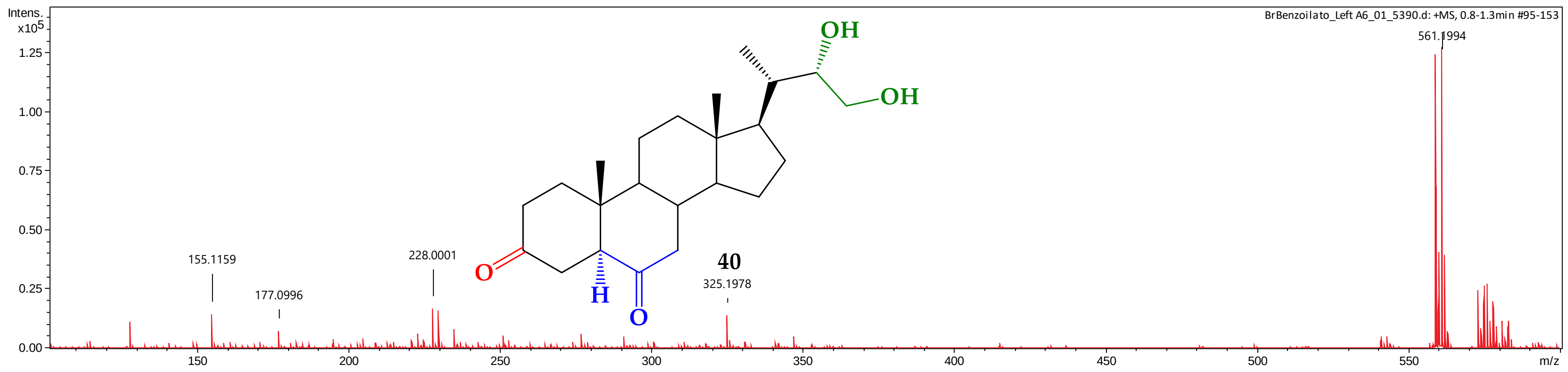

S57. HRSM-ESI spectrum of *22(S), 23-dihidroxy-24-nor-5α-cholan-3,6-dione* (**40**)

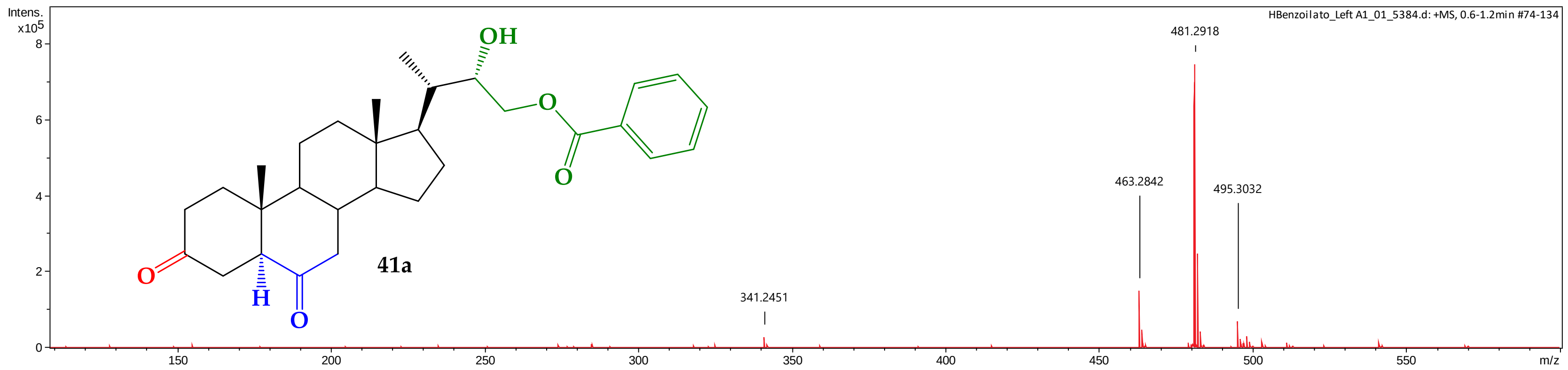

S58. HRSM-ESI spectrum of *22(S)-hydroxy-24-nor-5α-cholan-3,6-dioxobenzoate-23-yl* (***41a***)

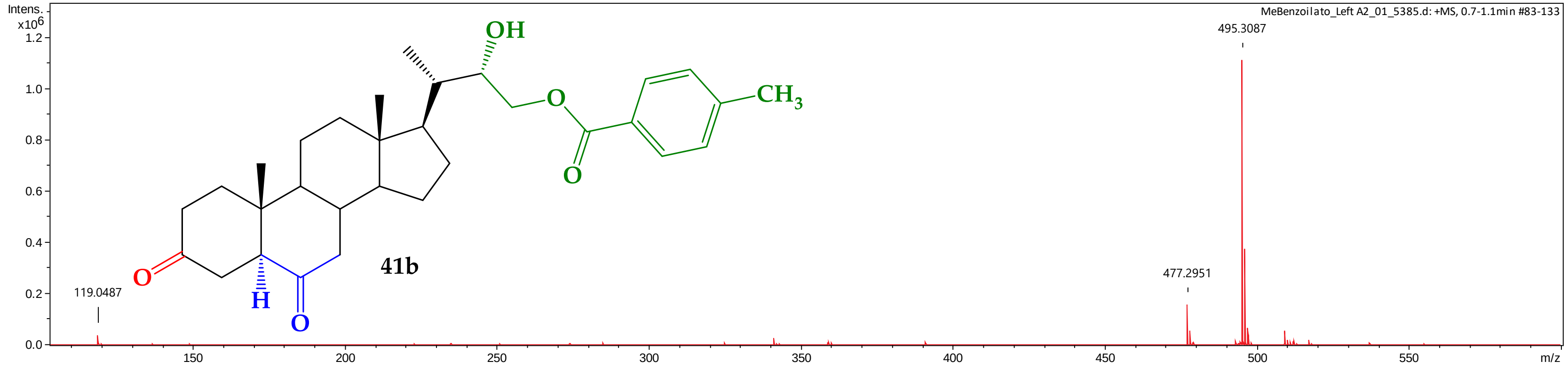

S59. HRSM-ESI spectrum of *22(S)-hydroxy-24-nor-5α-cholan-3,6-dioxo-(4-methyl)-benzoate-23-yl* (***41b***)

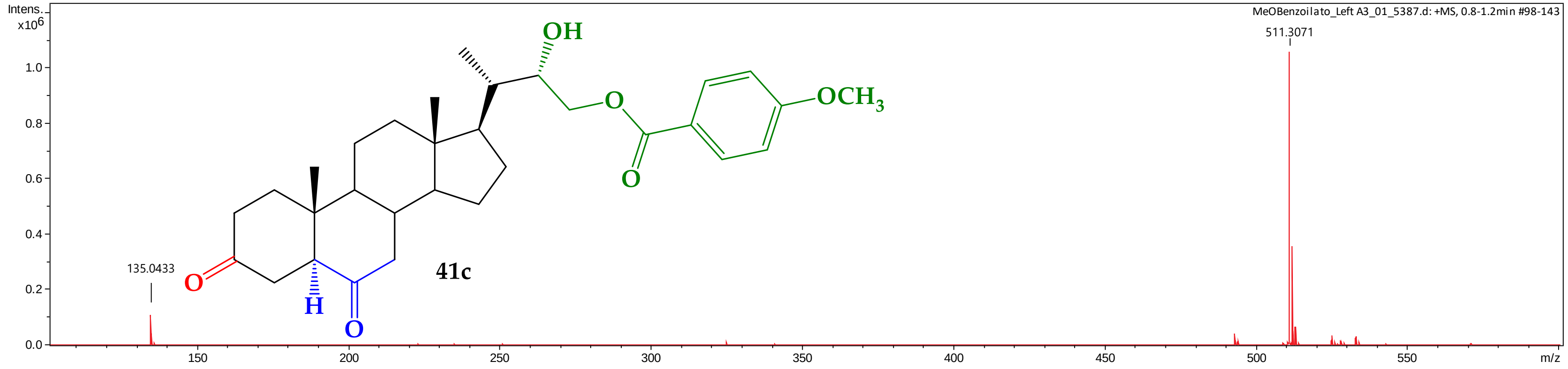

S60. HRSM-ESI spectrum of *22(S)-hydroxy-24-nor-5α-cholan-3,6-dioxo-(4-metoxy)-benzoate-23-yl* (***41c***)

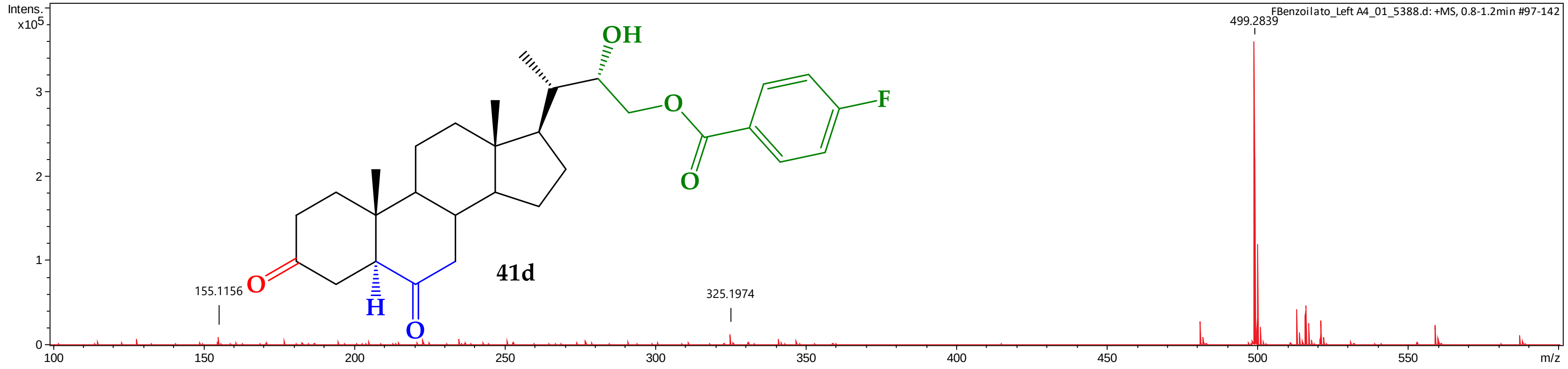

S61. HRSM-ESI spectrum of *22(S)-hydroxy-24-nor-5α-cholan-3,6-dioxo-(4-fluoro)-benzoate-23-yl* (***41d***)

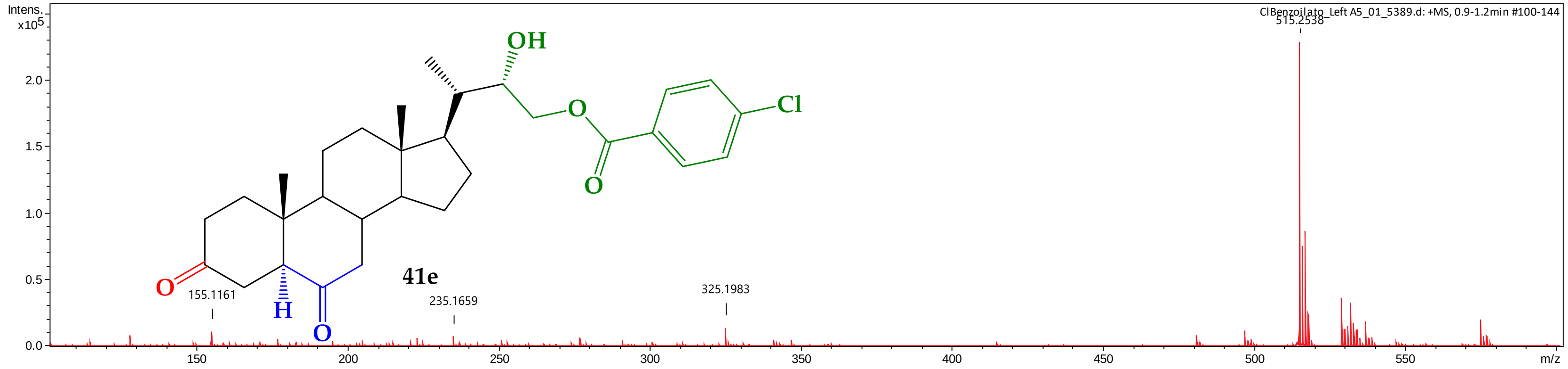

S62. HRSM-ESI spectrum of *22(S)-hydroxy-24-nor-5α-cholan-3,6-dioxo-(4-chloro)-benzoate-23-yl* (***41e***)

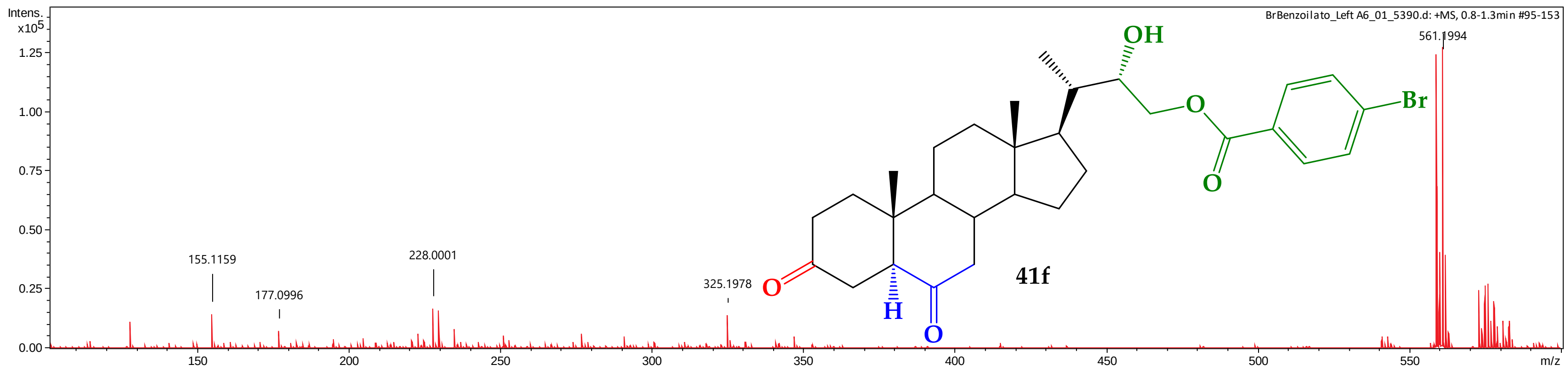

S63. HRSM-ESI spectrum of *22(S)-hydroxy-24-nor-5α-cholan-3,6-dioxo-(4-bromo)-benzoate-23-yl* (***41f***)

S64. Rice-lamina assays using the second leaf lamina joints (Angle Opening, Degrees) of excised leaf segments treated with BRs analogs (**40**, and **41a-41f**) at different concentrations: 1 x 10^{-8}, 1 x 10^{-7}, and 1 x 10^{-6} M. Brassinolide (**1**) was used as positive control at the same concentrations.

	Rice Lamina Inclination (Angle Opening, Degrees)		
**Compound**	**1x10^{-8} M**	**1x10^{-7} M**	**1x10^{-6} M**
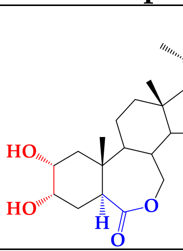 Brassinolide (**1**)	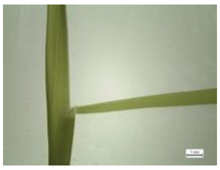 81±7.1	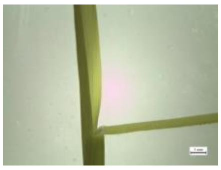 88±4.8	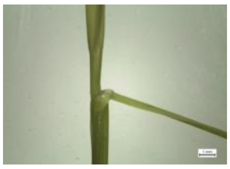 90±7.5
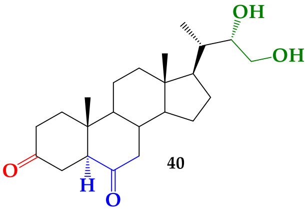 **40**	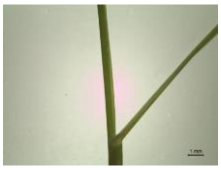 26±4.1	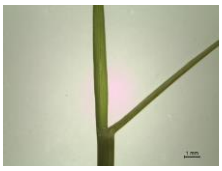 38±4.9	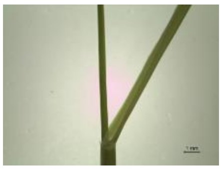 13±4.1
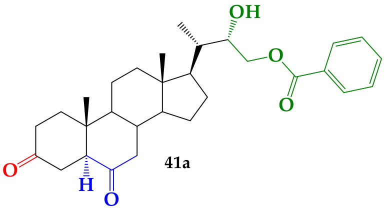 **41a**	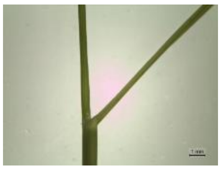 19±4.8	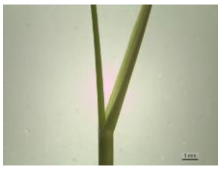 -	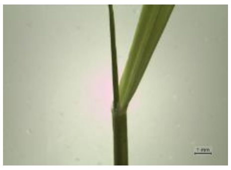 -
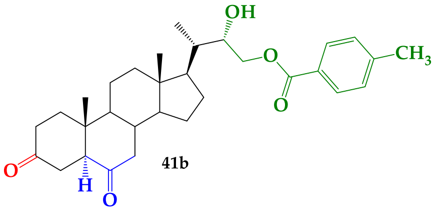 **41b**	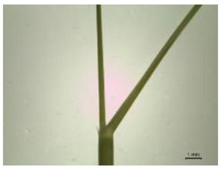 19±3.2	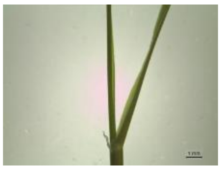 32±4.2	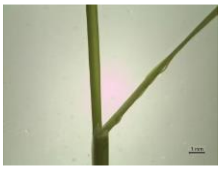 32±4.2
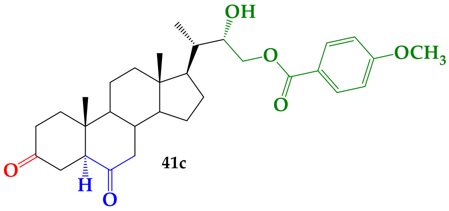 **41c**	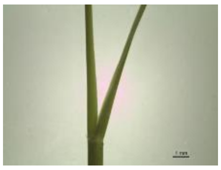 -	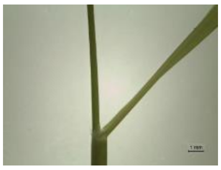 28±2.6	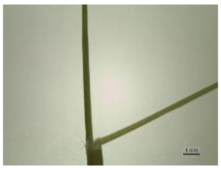 54±8.4
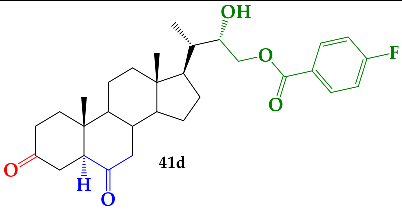 **41d**	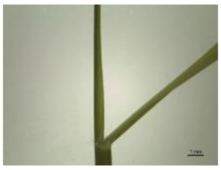 38±2.6	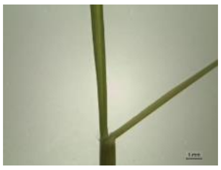 34±2.7	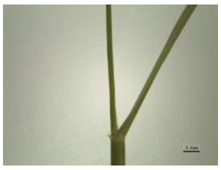 -

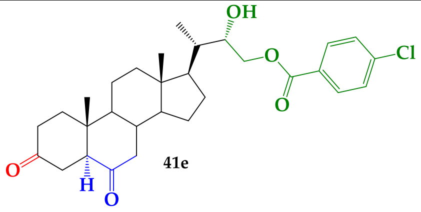	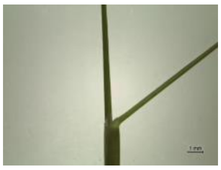 32±4.2	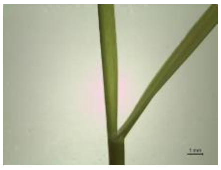 31±8.2	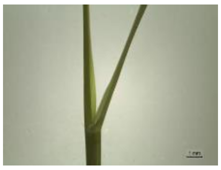 -
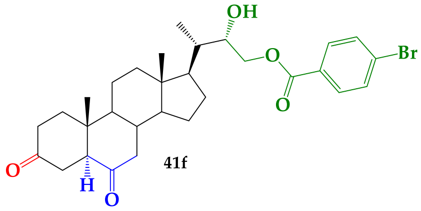	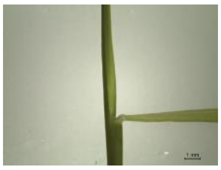 75±2.0	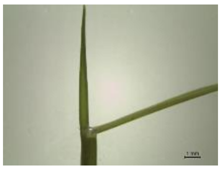 49±4.5	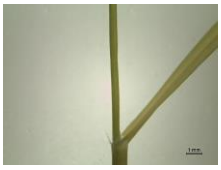 33±2.6
**Negative control (H_2O)**		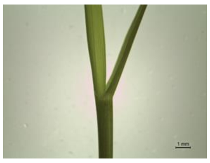 16±2.0	

S65. Effect of BRs analogues **40, 41a-41f**, and positive control (brassinolide (**1**)) on the elongation Bean Second-Internode Bioassay at a concentration of 1x10^{-8} M. Red line indicates the difference in elongation of the treatments.

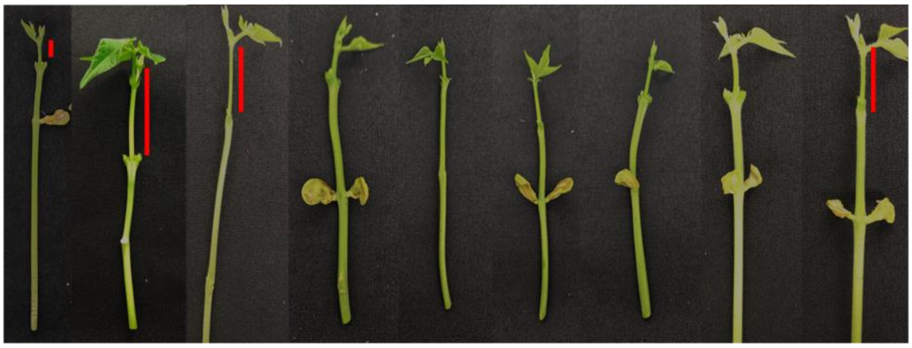

Negative Control **1** **40** **41a** **41b** **41c** **41d** **41e** **41f**

S66. *Protein-ligand interactions with a) brassinolide (**1**) and b) Compound **41f**. Hydrogen bonds are represented in green segmented lines. π-π stacking are represented in dark pink segmented lines. Hydrophobic interactions are represented in pink segmented lines. Visualization of the docked poses was performed using Discovery Studio Visualizer (BIOVIA, San Diego, CA, USA).*

a) Brassinolide (**1**)

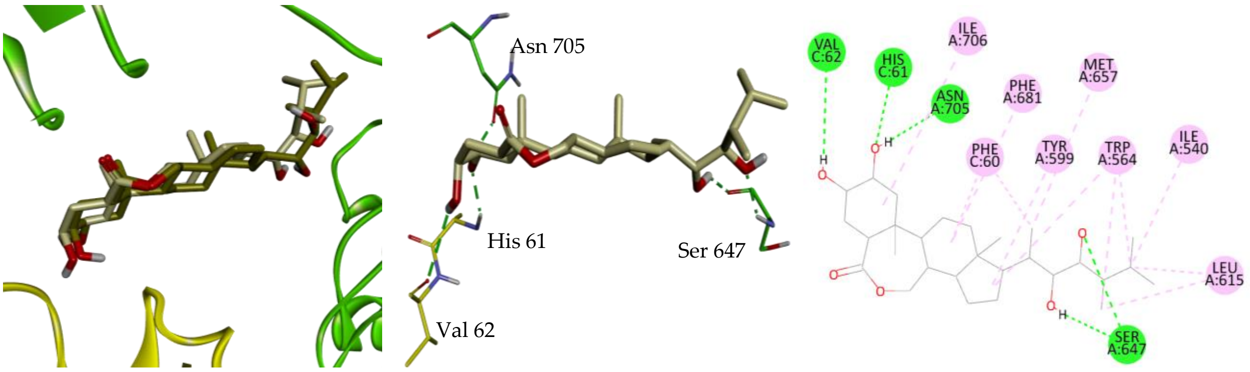

b) Compound **41f**

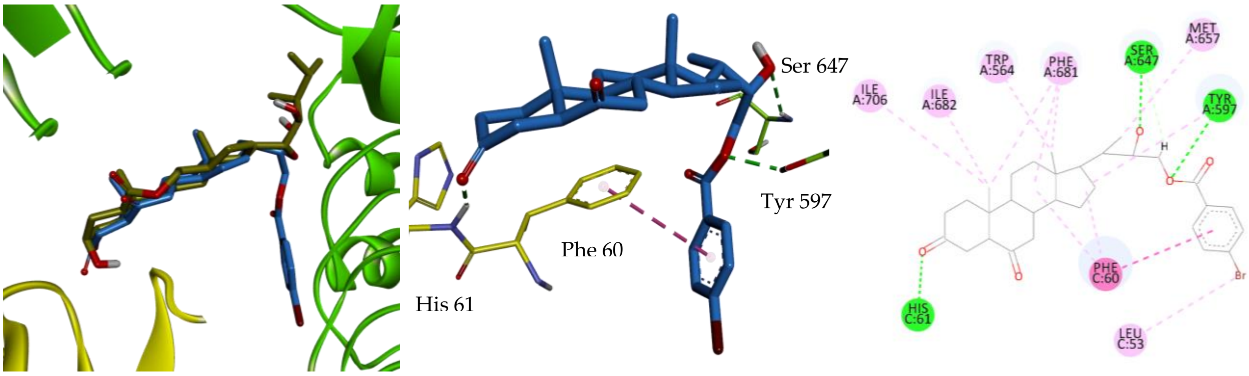

S67. Table 1. Pose analysis of docked brassinolide (**1**) and synthetic analog **41f**. ΔE_b: Binding Energy in kcal/mol.

Code	ΔE_b	Structure
**1**	-12.6	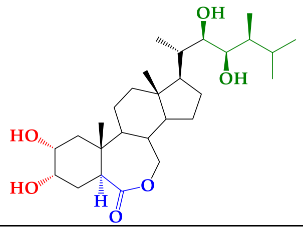

Code	ΔE_b	Structure
**41f**	-12.6	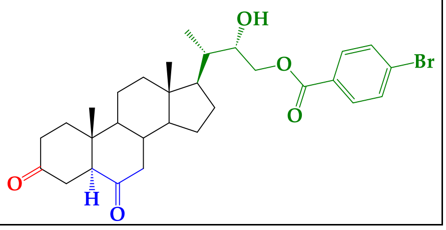

S68. Table 2. Docked compounds-heterodimer protein contacts of brassinolide (**1**) and synthetic analog **41f**.

Compound	**Protein Contacts**	
	Hydrogen bonds	Non-polar interactions
**1**	Asn705<br>His61<br>Val62<br>Ser647	Ile706, Tyr599, Phe681, Met657, Trp564, Ile540, Phe60, Leu 615
**41f**	Ser647<br>Tyr597<br>His61	Ile706, Ile682, Tyr597, Trp564, Met657, Phe60, Phe681, Leu53
